# Generation and characterization of a collection of knock-down lines for the chloroplast Clp protease complex in tobacco

**DOI:** 10.1093/jxb/erx066

**Published:** 2017-03-28

**Authors:** Juan C. Moreno, Nadine Tiller, Mercedes Diez, Daniel Karcher, Michael Tillich, Mark A. Schöttler, Ralph Bock

**Affiliations:** 1 Max-Planck-Institut für Molekulare Pflanzenphysiologie, Am Mühlenberg 1, D-14476 Potsdam-Golm, Germany

**Keywords:** Chloroplast, Clp protease, leaf development, *Nicotiana*, *tabacum*, photosynthesis, plastid, protease, protein degradation, protein stability.

## Abstract

Protein degradation in chloroplasts is carried out by a set of proteases that eliminate misfolded, damaged, or superfluous proteins. The ATP-dependent caseinolytic protease (Clp) is the most complex protease in plastids and has been implicated mainly in stromal protein degradation. In contrast, FtsH, a thylakoid membrane-associated metalloprotease, is believed to participate mainly in the degradation of thylakoidal proteins. To determine the role of specific Clp and FtsH subunits in plant growth and development, RNAi lines targeting at least one subunit of each Clp ring and FtsH were generated in tobacco. In addition, mutation of the translation initiation codon was employed to down-regulate expression of the plastid-encoded ClpP1 subunit. These protease lines cover a broad range of reductions at the transcript and protein levels of the targeted genes. A wide spectrum of phenotypes was obtained, including pigment deficiency, alterations in leaf development, leaf variegations, and impaired photosynthesis. When knock-down lines for the different protease subunits were compared, both common and specific phenotypes were observed, suggesting distinct functions of at least some subunits. Our work provides a well-characterized collection of knock-down lines for plastid proteases in tobacco and reveals the importance of the Clp protease in physiology and plant development.

## Highlight

By generating a large set of transgenic and transplastomic knock-down lines in tobacco, the phenotypic effects of impaired chloroplast protease function on plant growth and development, leaf morphology, pigmentation, and photosynthesis were characterized.

## Introduction

Most proteins have a limited lifetime and undergo turnover (Sakamoto, 2006). In addition, protein quality control is an essential process to detect and eliminate damaged proteins, thus maintaining cellular homeostasis (Gottesman *et al.*, 1997; Wickner *et al.*, 1999). Protein degradation (proteolysis) is also important for the regulation of metabolic and signaling pathways, and, therefore, disturbed protein homeostasis can have serious consequences.

In prokaryotes, protein degradation is carried out by a complex proteolytic machinery forming an intricate network of proteases. The Clp protease (caseinolytic protease) complex was discovered in *Escherichia coli* (Hwang *et al.*, 1987; Katayama-Fujimura *et al.*, 1987) and its structure was subsequently characterized in detail. Two main substructures were identified: a proteolytic core complex comprising the serine-type peptidase ClpP, and the chaperone complex consisting of the ATP-dependent chaperones ClpA and ClpX, both members of the AAA^+^ (ATPase Associated with various cellular Activities) superfamily. While Clp proteolytic activity is not essential in *E. coli* and many other bacteria, it is in cyanobacteria and plants (Maurizi *et al.*, 1990; Gottesman, 1996; Majeran *et al.*, 2000; Shikanai *et al.*, 2001; Kuroda and Maliga, 2003).

**Fig. 1.**
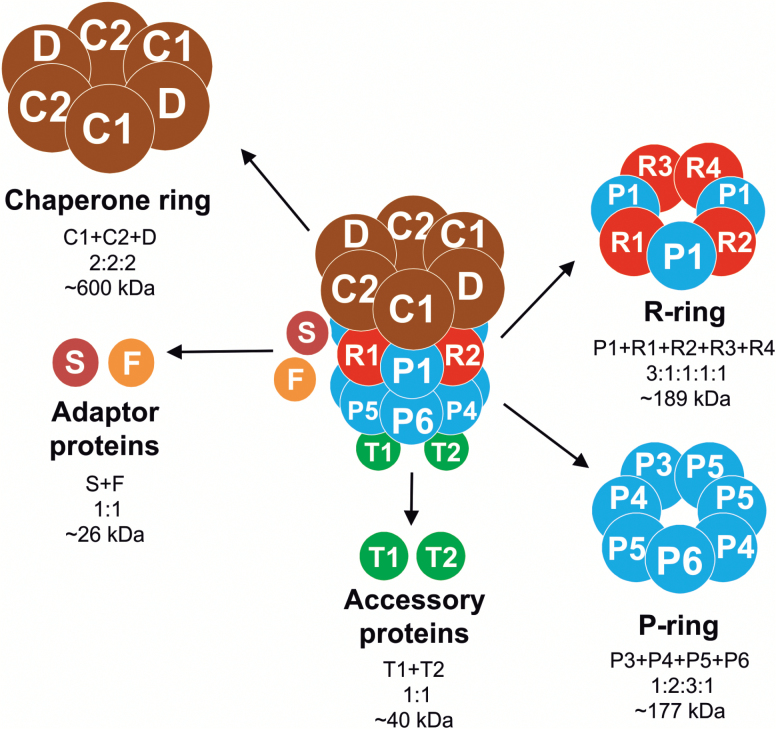
Schematic representation of the Clp protease complex in Arabidopsis. The complex composition with all subunits identified to date is shown. CLPP proteolytic subunits (P subunits) are indicated in blue and Clp-related subunits (R subunits) lacking proteolytic activity are in red. The chaperone ring subunits are in brown. Accessory proteins (CLPT1 and CLPT2) are indicated in green, and adaptor proteins of the complex are shown in light brown (CLPS) and orange (CLPF). The subunit stoichiometry and size (in kDa) of the three rings and the other constituents of the complex are also given.

Consistent with their cyanobacterial origin, plastids (chloroplasts) harbor a prokaryote-related proteolytic machinery. Plastids change their morphology in response to environmental stimuli and developmental transitions. Therefore, dynamically adjusting the plastid proteome by controlling the abundance of both nucleus-encoded and plastid-encoded proteins is essential. Proteases play an important role in the interconversion of plastid types and the maintenance of plastid homeostasis (Kato and Sakamoto, 2010). Biochemical and genetic studies have unraveled several proteolytic activities in plastids (Sakamoto, 2006). The plastid-localized proteases identified so far are homologous to bacterial proteases, and include the major ATP-dependent proteases Clp, FtsH, and Lon, the major ATP-independent protease Deg (Shanklin *et al.*, 1995; Lindahl *et al.*, 1996; Itzhaki *et al.*, 1998), and several minor proteases (Timmis *et al.*, 2004). Interestingly, many of these proteases and/or their subunits are encoded by multigene families, and the emerging specific functions of individual family members suggest an intricate network of regulated proteolysis (Rudella *et al.*, 2006; Kapri-Pardes *et al.*, 2007; Kim *et al.*, 2009). The major proteases Clp and Lon are believed to be chiefly responsible for the degradation of stromal proteins, whereas FtsH and Deg may degrade mainly thylakoid proteins (Adam, 2000; Adam *et al.*, 2006; Sakamoto, 2006). The Clp protease represents the most abundant and complex stromal protease (Olinares *et al.*, 2011*a*). Clp is believed to play a central role in protein quality control as a housekeeping protease (Clarke *et al.*, 2005). The structure of the Clp machinery has been largely conserved throughout evolution and consists of a barrel-like protease core and the AAA^+^ chaperone ring (Nishimura and van Wijk, 2015) (Fig. 1). In Arabidopsis, the Clp core complex is composed of two heptameric rings with a mol. wt of ~350 kDa (Fig. 1). ClpP subunits have proteolytic activity, while ClpR (Clp-related) subunits appear to lack catalytic activity. The heptameric P-ring is formed by CLPP3, CLPP4, CLPP5, and CLPP6 subunits (in 1:2:3:1 stoichiometry), and each of these subunits possesses catalytic activity (Fig. 1). The R-ring is composed of CLPR1–CLPR4 subunits and ClpP1, the only subunit encoded in the plastid genome (Olinares *et al.*, 2011*b*) and the only catalytic subunit within the R-ring (Fig. 1). The main function of the R-ring may lie in the stabilization of the core complex (Peltier *et al.*, 2004; Sjögren et al., 2006; Olinares *et al.*, 2011*b*). The chaperone ring is composed of two copies of CLPC1, CLPC2, and CLPD, respectively, which all belong to the AAA^+^ family (Fig. 1). This hexameric ring is attached to the R-ring, and its chaperone subunits are capable of triggering conformational changes in substrate proteins (Hanson and Whiteheart, 2005; Diemand and Lupas, 2006). Two adaptor proteins believed to mediate substrate recognition have been identified so far in plastids: CLPS and CLPF (Nishimura *et al.*, 2013, 2015). The CLPT1 and CLPT2 subunits are unique in land plants. They show high homology to the N-terminal domain of ClpC chaperones (Kim *et al.*, 2015) and may be involved in stabilization of the core complex (Kim *et al.*, 2015).

The membrane-associated protease FtsH forms heterocomplexes in the inner membrane of mitochondria and in the thylakoid and envelope membranes of plastids (Yu *et al.*, 2004; Urantowka *et al.*, 2005; Wagner *et al.*, 2012). In Arabidopsis chloroplasts, the FtsH heterocomplex is formed by two isoforms, designated A and B, encoded by *FTSH1/FTSH5* and *FTSH2/FTSH8*, respectively (Sakamoto *et al.*, 2003; Yu *et al.*, 2004). By participating in the degradation of the PSII reaction center protein D1, FtsH complexes contribute to the PSII repair cycle (Lindahl *et al.*, 2000; Bailey *et al.*, 2002; Kato and Sakamoto 2009; Kato *et al.*, 2009).

So far, the functions of the Clp and FtsH subunits have mainly been addressed by mutant analysis in Arabidopsis (e.g. Sakamoto *et al.*, 2003; Zaltsman *et al.*, 2005*a*; Sjögren *et al.*, 2006; Kim *et al.*, 2013). Mutations in any of the nuclear-encoded subunits of the core complex (CLPP3–CLPP6 and CLPR1–CLPR4) result in pigment-deficient phenotypes and defects in plastid biogenesis (Rudella *et al.*, 2006; Sjögren *et al.*, 2006; Zheng *et al.*, 2006; Koussevitzky *et al.*, 2007). Lack of both CLPC1 and CLPC2 prevents the formation of viable embryos (Kovacheva *et al.*, 2007). *clpT1-clpT2* double mutants show a pale phenotype (Kim *et al.*, 2015), while single mutants for the adaptor proteins CLPS and CLPF as well as the *clps-clpf* double mutant display wild-type-like phenotypes. In contrast to the *clp* mutants, *ftsh* mutants show a variegated phenotype accompanied by impaired plant growth and development (Sakamoto *et al.*, 2003; Zaltsman *et al.*, 2005*a*).

In this work, we characterized the Clp protease complex in tobacco by generating a set of knock-down lines with reduced abundance of individual subunits. In contrast to Arabidopsis, tobacco offers the possibility also to address the function of the plastid-encoded subunit ClpP1 by reverse genetics. Moreover, tobacco is the preferred host for recombinant protein expression in chloroplasts (Maliga, 2004; Bock and Warzecha, 2010; Bock, 2015), and protein stability has emerged as the key factor that limits recombinant protein accumulation (Birch-Machin *et al.*, 2004; Elghabi *et al.*, 2011; De Marchis *et al.*, 2012). Thus, in addition to obtaining insights into the functions of the Clp protease complex, manipulation of the activity of the protease also offers the possibility to improve the stability of recombinant proteins produced in plastids for biotechnological purposes (e.g. pharmaceutical proteins and industrial enzymes). Here, we used site-directed mutagenesis and RNAi strategies to decrease the expression of plastid-encoded and nucleus-encoded subunits of the Clp complex in tobacco. We also silenced the major FtsH protease in the thylakoid membrane to assess comparatively the roles of the Clp and FtsH proteases.

## Materials and methods

### Identification of gene sequences for Clp and FtsH subunits from tobacco and selection of regions for silencing by RNAi

For identification of *CLP* and *FTSH* coding sequences from tobacco, the amino acid sequences of the annotated Arabidopsis and tomato orthologs (see Supplementary Table S1 at *JXB* online) were collected from TAIR10 (The Arabidopsis Information Resource, www.arabidopsis.org) and ITAG2.3 (International Tomato Annotation Group, www.solgenomics.net), respectively, and used as queries in tBLASTn (Camacho *et al.*, 2009) searches with default parameters against three local BLAST databases. The databases comprised the available tobacco nucleotide sequences from the NCBI EST, GSS, and SRR archives (www.ncbi.nlm.nih.gov; downloaded on 17 December 2011). The SRR database was extended by in-house available next-generation sequencing (NGS) reads from tobacco (Stegemann *et al.*, 2012). Based on partially degenerate alignments (Supplementary Table S1; for details see Supplementary Protocols), sequence segments, 200–300 bp in length, with the lowest possible number of single nucleotide polymorphisms (SNPs) between the parental alleles were selected as target regions for gene silencing by RNAi (Supplementary Table S3) and amplified by PCR. In order to silence *CLPT1* and *CLPT2* simultaneously, a 200 bp region containing both parental sequence versions for *CLPT1* and a 200 bp region specific for *CLPT2* were separately amplified and fused by a third PCR. With the resulting 400 bp *CLPT1–CLPT2* region, it was possible to trigger the silencing of both genes. The sequence of all amplicons was determined by Sanger sequencing (Eurofins MWG operon) which confirmed the predicted coding sequences.

### Phylogenetic analyses

**Fig. 2.**
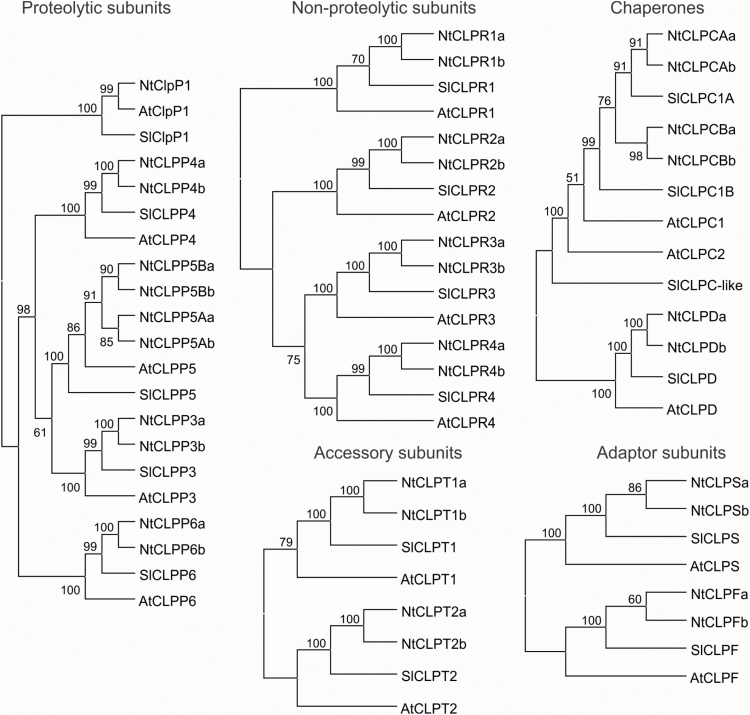
Phylogenetic trees of selected Clp protease subunits from Arabidopsis (At), tomato (Sl), and tobacco (Nt). Arabidopsis and tomato identifiers refer to single proteins, whereas the Nt sequences represent two distinct but highly homologous tobacco proteins (designated a and b), in line with the allotetraploid origin of the nuclear genome of *Nicotiana tabacum* (comprising the diploid genomes of *N. sylvestris* and *N. tomentosiformis*).

The final contigs based on ESTs and short reads were extended by transcripts from *Nicotiana tabacum* (BioProject: PRJNA319578), *N. sylvestris* (PRJNA257217), and *N. tomentosiformis* (PRJNA257218) upon their availability at NCBI (www.ncbi.nlm.nih.gov). In most cases, this confirmed the sequences of the final contigs and allowed completion of the few partial coding sequences and separation of the parental gene variants (designated a and b) (Fig. 2). The consensuses were inspected, *in silico* translated with BioEdit (Hall, 1999), and used for phylogenetic analysis. In order to study the relatedness of the sequences, phylogenic trees (ML and UPGMA) for the protein sequences belonging to each subset (CLPP/CLPR/ClpP, CLPC/D, CLPS/CLPF, CLPT, and FTSH) were generated with MEGA7 (Kumar *et al.*, 2016) based on alignments by MUSCLE (Edgar, 2004) with default parameters. The UPGMA consensus trees (*n*=500; nodes with <50% support were collapsed) are shown.

### Plant material and growth conditions

Tobacco (*N. tabacum*) wild type, transgenic, and transplastomic lines were raised from seeds germinated in Petri dishes containing Murashige and Skoog (MS) medium supplemented with 30 g l^–1^ sucrose (Murashige and Skoog, 1962). Kanamycin (100 μg ml^–1^) was used for selection of nuclear-transgenic plants and 500 μg ml^–1^ spectinomycin was used for selection of transplastomic plants. For photosynthetic measurements, seedlings were transferred 14 d after germination to a soil–vermiculite mixture (2:1) and grown in a controlled-environment chamber at 350 μmol photons m^–2^ s^–1^ light intensity (16 h day, 22 °C, 75% relative humidity). For growth-related measurements (plant height, leaf surface, and flower and leaf numbers), sampling, and seed production, plants were grown under standard greenhouse conditions.

### *Construction of plastid transformation vectors for knock-down of* clpP

**Fig. 3.**
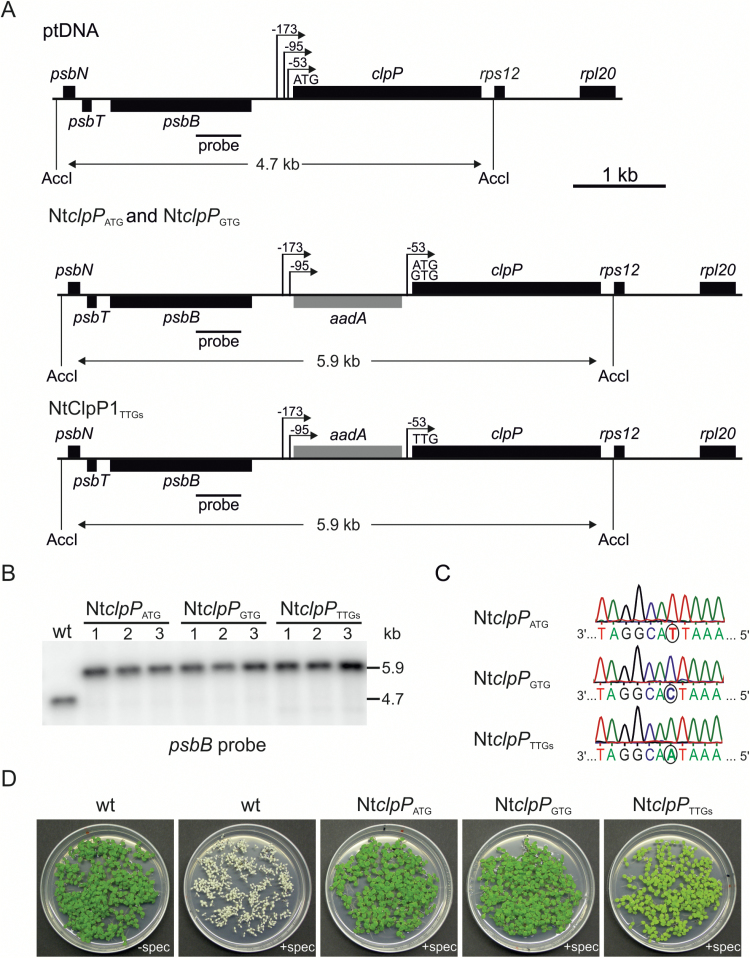
Generation of transplastomic tobacco plants with reduced expression of ClpP1. (A) Physical maps of the targeting region in the plastid genome of wild-type tobacco (ptDNA) and the plastid transformants Nt*clpP*_ATG_, Nt*clpP*_GTG_, and Nt*clpP*_TTGs_. To lower the translational efficiency of the *clpP* mRNA, its start codon was mutated from AUG to GUG (Nt*clpP*_GTG_) or from AUG to UUG (Nt*clpP*_TTGs_). The chimeric selectable marker gene *aadA* conferring resistance to spectinomycin and streptomycin is driven by the rRNA operon promoter (*Prrn*) and a synthetic Shine–Dalgarno sequence (Svab and Maliga, 1993). The 3'-untranslated region of the *psbA* gene (3'*psbA*) stabilizes the mRNA. The *aadA* cassette was introduced either in antisense (Nt*clpP*_GTG_ and Nt*clpP*_ATG_) or in sense (Nt*clpP*_TTGs_) direction relative to *clpP*. The expected sizes of the DNA fragments in RFLP analyses with the restriction enzyme *Acc*I are indicated below each map in kilobases. The location of the RFLP probe is indicated as a black bar. (B) RFLP analysis of transplastomic *clpP* mutants. Genomic DNA extracted from ~100 seedlings of the T_1_ generation was digested with *Acc*I and hybridized to a radiolabeled probe detecting the plastid genome region flanking the transgene insertion site. (C) Sequence analysis of the *clpP* start codon. The same genomic DNA samples used for RFLP analysis were used to amplify the region surrounding the *clpP* start codon and sequence the resulting PCR products. The chromatograms of the sequencing reactions show the homoplasmic state of the mutated version of the *clpP* start codon in the Nt*clpP*_GTG_ and Nt*clpP*_TTGs_ mutants. (D) Inheritance assay of transplastomic *clpP* mutant plants. Germination of the T_1_ generation of plastid transformants in the presence of spectinomycin (+spec) revealed the homoplasmic stage for the integrated selectable marker gene *aadA*. wt: wild type.

A region of the plastid genome containing 365 bp of the *psbB* gene and the intergenic region of *clpP* and *psbB* was isolated as a 733 bp *Ssp*I restriction fragment corresponding to plastome positions 74 588–75 320 (Fig. 3A). The fragment was cloned into a pBS SK vector linearized with *Ecl*136II. The resulting plasmid was cut with *Eco*RI and *Sma*I, and a 1338 bp *Ssp*I/*Eco*RI fragment of the plastid genome region containing 1261 bp of the *clpP* gene and part of the intergenic region between *clpP* and *psbB* (corresponding to plastome positions 73 250–74 588) was inserted.

A chimeric *aadA* gene fused to chloroplast-specific expression signals and conferring resistance to spectinomycin (Svab and Maliga, 1993) was cloned into a unique *Not*I site to enable selection of transplastomic lines. The resulting constructs contained the *aadA* selectable marker gene in the opposite (Nt*clpP*_ATG_) (Fig. 3A) or in the same orientation relative to the *clpP* gene. They served directly as transformation vectors (*aadA* control line; Nt*clpP*_ATG_) and were additionally used to produce translation initiation codon mutations using the QuickChange^®^II Site-Directed Mutagenesis Kit (Agilent) in combination with oligonucleotides clpP_GTG_for and clpP_GTG_rev, yielding transformation vectors *clpP*_GTG_ (*aadA* in antisense orientation) (Fig. 2A) and *clpP*_GTGs_ (*aadA* in sense orientation to the *clpP* gene, not shown), and oligonucleotides clpP_TTG_for and clpP_TTG_rev (Supplementary Table S4), yielding transformation vectors *clpP*_TTGs_ (*aadA* in sense orientation to *clpP*) (Fig. 2) and *clpP*_TTG_ (not shown).

### Plastid transformation and selection of transplastomic tobacco plants

Plastid transformation was performed using the biolistic protocol (Svab and Maliga, 1993). Young leaves of aseptically grown *N. tabacum* (cv. Petit Havana) plants were bombarded with plasmid DNA-coated 0.6 μm gold microcarriers (BioRad) using the PDS-100/He device (BioRad). Primary spectinomycin-resistant shoots were selected on regeneration medium with 500 μg ml^–1^ spectinomycin. Chloroplast transformation was confirmed by testing for double resistance on medium containing spectinomycin and streptomycin (500 μg ml^–1^ each; Svab and Maliga, 1993; Bock, 2001). Transplastomic plants were subjected to 2–3 additional rounds of regeneration in the presence of spectinomycin to enrich the transgenic plastome and select against residual wild-type genome copies. Homoplasmy was confirmed by Southern blot analyses, seed tests, and resequencing of the *clpP* start codon. Homoplasmic transplastomic lines were transferred to the greenhouse for seed production.

### RFLP analyses and hybridization procedures

For restriction fragment length polymorphism (RFLP) analysis, DNA samples (3 μg of total cellular DNA) were treated with the restriction enzyme *Acc*I, separated in 1% agarose gels, and blotted onto Hybond XL membranes (GE Healthcare). For northern blot analysis, RNA samples were electrophoretically separated in formaldehyde-containing 1.5% agarose gels and transferred onto Hybond XL membranes (GE Healthcare) by capillary blotting using standard protocols. A 411 bp *Mfe*I/*Spe*I restriction fragment covering the first exon of *clpP* was purified by agarose gel electrophoresis, and used as a hybridization probe. Hybridization probes for *psbB* and *psaB* were generated by PCR using gene-specific primers (PpsbB_for and PpsbB_rev; PpsaB_for and PpsaB_rev; Supplementary Table S4). The obtained amplification products of 490 bp and 550 bp, respectively, were purified by agarose gel electrophoresis. Probes were labeled with [α-^32^P]dCTP by random priming (GE Healthcare). Hybridizations were performed at 65 °C in Rapid-Hyb buffer (GE Healthcare) according to the manufacturer’s protocol.

### Generation of RNAi constructs and nuclear transformation

Using the pENTR™ directional TOPO^®^ Cloning Kit, PCR-derived DNA fragments were cloned into pENTR/SD/D-TOPO^®^ (Invitrogen) according to the manufacturer’s protocol. The PCR products (used for Gateway cloning) were amplified with a forward primer containing CACC at its 5' end (Supplementary Table S4) to match the overhang in the cloning vector (GTGG). This entry vector was then used to perform a Gateway recombination reaction generating the final expression vector pK7GWIWG2 (I) with the RNAi regions in sense and antisense orientation for the CLPP6, CLPR2, CLPC, CLPS, CLPT1-T2, and FTSH1-5 subunits. Gateway^®^ LR cloning (Invitrogen) reactions were carried out following the manufacturer’s instructions. Transformation of the CLPP6, CLPR2, CLPC, CLPS, CLPT1-T2, and FTSH1-5 RNAi constructs into tobacco (*N. tabacum* cv. Petit Havana) was done by *Agrobacterium tumefaciens*-mediated gene transfer using bacterial strain C58C1:pGV2260 (Rosahl *et al.*, 1987).

### cDNA synthesis

Prior to reverse transcription, isolated RNAs were tested for the presence of contaminating DNA by a standard PCR using 1 ng of RNA as template. If no DNA amplification was observed, cDNA was synthesized using SuperScript III reverse transcriptase (Invitrogen, Carlsbad, CA, USA) according to the manufacturer’s instructions.

### Isolation of nucleic acids

Total plant DNA was extracted by a cetyltrimethylammonium bromide (CTAB)-based method (Doyle and Doyle 1990). Total cellular RNA was isolated with the NucleoSpin RNA Plant kit (Macherey-Nagel) following the manufacturer’s protocol.

### PCR, DNA sequencing, and quantitative real-time PCR (qRT-PCR)

PCR amplifications were carried out using the GoTaq^®^ Flexi DNA polymerase (Promega) following the manufacturer’s protocol. The standard PCR program was 35 cycles of 15 s at 94 °C, 30 s at 55 °C, and 60 s at 72°C, with a 2 min extension of the first cycle at 94 °C and a 5 min final extension at 72 °C. PCR products were analyzed by electrophoresis in 1% (w/v) agarose gels. For genotyping of transplastomic lines by DNA sequencing of the *clpP* start codon, the corresponding region in the plastid genome was amplified by PCR using the following oligonucleotides: Pseq_clpP_start in combination with PaadA136 for mutants with the *aadA* gene in antisense orientation, and Pseq_clpP_start in combination with PaadA25a (Supplementary Table S4) for mutants with the *aadA* in sense orientation. Amplification products were separated by agarose gel electrophoresis, purified from excised gel slices using the NucleoSpin Extract II kit (Macherey-Nagel), and sequenced using primer Pseq_clpP_start (MWG Biotech).

qRT-PCR was performed in a LightCycler 480 (Roche, Mannheim, Germany) using SYBR green I Master mix according to the manufacturer’s instructions. Three independent lines and three technical replicates per line were analyzed. The relative transcript levels were determined using the formula (1+E)^–ΔΔCt^ where E is the binding efficiency of the primers (Pfaffl, 2001). To ensure correct normalization of the investigated genes, the expression levels of several reference genes previously described for tobacco (Schmidt and Delaney, 2010) were tested, including the genes encoding clathrin adaptor protein (homologous to At5g46630), SAND family protein (homologous to At2g28390), and ubiquitin-conjugating enzyme E2 (homologous to At2g02760). Results were normalized to the mRNA levels of ubiquitin/clathrin as housekeeping genes. Primers used to measure the relative expression of Clp and FtsH subunits are listed in Supplementary Table S4.

### Protein isolation and immunoblotting

Total soluble protein was extracted and quantified according to Oey *et al.* (2009). Samples were electrophoretically separated in 10% Tricine/SDS–polyacrylamide gels (Schägger and Jagow, 1987), and the proteins were either directly visualized by Coomassie blue staining or transferred to Hybond-P PVDF membranes (GE Healthcare) using a Trans-Blot cell (Bio-Rad) and a standard transfer buffer (192 mM glycine, 25 mM Tris, pH 8.3). Immunoblot detection was performed with a ClpP1-specific antibody (Uniplastomic, Gieres, France) using an enhanced chemiluminescence system (ECL^®^ PLUS; GE Healthcare).

### Photosynthesis measurements

Measurements of chlorophyll content and the Chl *a*/*b* ratio were done with a Jasco V-630 photometer (Jasco GmbH, Groß-Umstadt, Germany) in 80% (v/v) acetone (Porra *et al.*, 1989). *In vivo* Chl *a* fluorescence of intact plants was measured using a DUAL-PAM-100 instrument (Heinz Walz GmbH, Effeltrich, Germany) after 20 min of dark adaptation. Measurements were performed and analyzed according to published procedures (Baker *et al.*, 2007).

Light–response curves of leaf gas exchange were measured with a GFS-3000 open gas exchange system (Heinz Walz GmbH) equipped with an LED array unit 3055-FL as actinic light source. Measurements were performed at 22 °C cuvette temperature with 17500 ppm humidity and a saturating CO_2_ concentration of 2000 ppm, to repress photorespiration fully. After respiration was determined in darkness, the actinic light intensity was stepwise increased to 100, 200, 350 (growth light intensity), 500, 1000, and finally 1500 µE m^−2^ s^−1^, when all plants were light saturated. At each actinic light intensity, gas exchange was recorded until the steady state of transpiration and leaf assimilation had been reached.

## Results

### Identification of putative Clp and FtsH sequences and selection of RNAi regions for gene silencing in tobacco

Using available databases (see the Materials and methods) and the draft tobacco genome sequence (Sierro *et al.*, 2014), 14 Clp sequences and 2 FtsH sequences were assembled (Supplementary Table S2). To support the identity of these newly assembled sequences, we used available online tools (WOLF-PSORT, Y-loc, and plant-mPLoc) to confirm the presence of chloroplast transit peptides in the N-termini (Supplementary Table S5). In addition, motif and domain analyses were conducted to confirm the presence of conserved motifs in protease subunits. Phylogenetic analyses ultimately confirmed the identity of the assembled sequences and revealed that, for most subunits, two isoforms are encoded in the tobacco nuclear genome. This is due to the allotetraploid origin of *N. tabacum* (from two diploid progenitor species: *N. sylvestris* and *N. tomentosiformis*). As expected, the tobacco genes and proteins are more closely related to the orthologs from tomato than those from Arabidopsis. Interestingly, the only exception is the chloroplast-encoded ClpP1 protein which is more similar to the Arabidopsis ClpP1 (Fig. 2; Supplementary Fig. S1). Six Clp subunits, nucleus and plastid encoded, including representative subunits from each ring of the protease, were selected to be targeted by reverse genetics. The nucleus-encoded genes were down-regulated by an RNAi approach, whereas for the plastid-encoded ClpP1, site-directed mutagenesis of the translation initiation codon was attempted. In addition, the thylakoidal FtsH protease was selected as another major protease that is likely to have different substrate proteins. Gene fragments of 250–400 bp in size that harbor a low number of SNPs between the two tobacco alleles (to ensure silencing of both gene versions) were chosen as target regions for RNAi (Supplementary Table S3).

### *Site-directed mutagenesis of the plastid* clpP *gene*

Gene disruption studies of the plastid genome-encoded subunit ClpP1 had revealed the essentiality of the *clpP* gene for chloroplast function and plant development (Shikanai *et al.*, 2001; Kuroda and Maliga, 2003). To study ClpP1 function, we reduced *clpP* gene expression by mutating the AUG translation initiation codon. In contrast to transcriptionally regulated nuclear genes, plastid gene expression is predominantly regulated at the translational level (Sugiura *et al.*, 1998; Eberhard *et al.*, 2002; Kahlau and Bock, 2008; Valkov *et al.*, 2009). Mutation of the translation initiation codon into the alternative (and usually less efficient) start codons GUG or UUG has proven a successful strategy to reduce the expression of plastid genes by down-regulating their translation (Hirose *et al.*, 1999; Hirose and Sugiura, 2004, Rott *et al.*, 2011), including that of the *clpP1* gene in the green alga *Chlamydomonas reinhardtii* (Majeran *et al.*, 2000).

To generate plastid transformation vectors with mutated *clpP* start codons, we inserted the selectable marker gene *aadA* upstream of *clpP* in sense (Nt*clpP*_ATGs_) and antisense (Nt*clpP*_ATG_) orientation, respectively (Fig. 3A). The *aadA* cassette (conferring spectinomycin resistance) was placed between the two *clpP* promoters P*clpP-53* and P*clpP-95* (Hajdukiewicz *et al.*, 1997) (Fig. 3A). The resulting constructs (with the native start codon AUG) served as controls and were also used to mutagenize the initiation codon from A to G (Nt*clpP*_GTGs_ and Nt*clpP*_GTG_) or A to T (Nt*clpP*_TTG_ and Nt*clpP*_TTGs_). All constructs were introduced into the tobacco plastid genome by biolistic transformation (Svab and Maliga, 1993).

Several independently generated transplastomic lines were obtained and characterized in detail. The lines were purified to homoplasmy under continuous monitoring of the point mutation by DNA resequencing in each regeneration round (to prevent loss of the mutation by gene conversion; Khakhlova and Bock, 2006). Homoplasmy (i.e. absence of residual wild-type plastid genomes from the transplastomic plants) was ultimately confirmed by inheritance assays, RFLPs, and resequencing of the *clpP* start codon in the T_1_ generation (Fig. 3B–D). Whereas wild-type seedlings grown on spectinomycin-containing medium bleached, the T_1_ generation of all homoplasmic transplastomic lines was uniformly green. Based on plant phenotypes and preliminary assessment of ClpP1 protein levels, the control line Nt*clpP*_ATG_ and the two ClpP1 down-regulation lines Nt*clpP*_GTG_ and Nt*clpP*_TTGs_ were selected for in-depth characterization. Nt*clpP*_TTGs_ plants displayed the strongest visual phenotype, and all seedlings from these transplastomic lines were strikingly pale green in seed assays (Fig. 3D). When genomic DNA extracted from a pool of ~100 T_1_ seedlings was subjected to RFLP analysis, the expected fragment of 4.7 kb was detected in *Acc*I-restricted wild-type DNA, whereas a fragment of 5.9 kb was exclusively present in the transplastomic plants (Fig. 3B), confirming the absence of residual wild-type copies of the plastid genome. Likewise, sequencing of the region around the *clpP* start codon revealed the exclusive presence of the altered codons in both mutants (Nt*clpP*_GTG_ and Nt*clpP*_TTGs_) (Fig. 3C).

### *Analysis of* clpP *expression in transplastomic mutants*

**Fig. 4.**
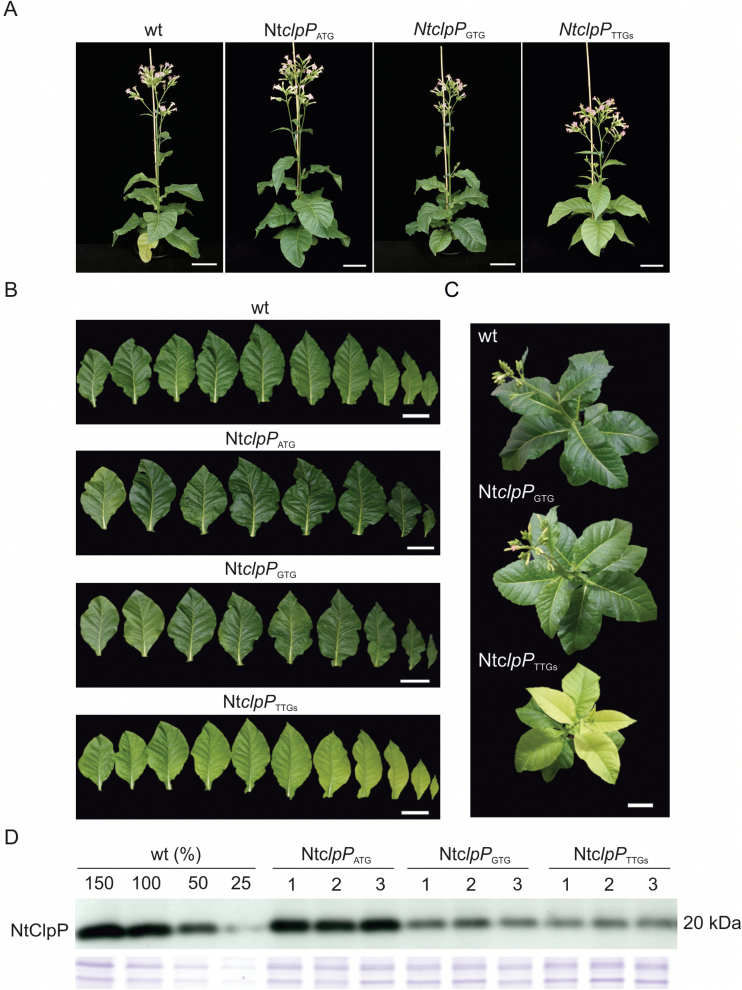
Down-regulation of the chloroplast-encoded ClpP1 subunit in tobacco. (A) Growth of the wild type (wt) and homoplasmic Nt*clpP* mutant lines. Plants were raised from seeds and photographed after 11 weeks of growth in the greenhouse. (B) Detached leaves from 10-week-old Nt*clpP* plants. Shown is a series of all true leaves from the bottom (left) to the top (right) of an individual plant. (C) Nine-week-old wild-type and Nt*clpP* plants photographed from the top. Note the yellow young leaves and the greener mature leaves of the Nt*clpP*_TTGs_ mutant. Scale bars=10 cm. (D) Accumulation of the ClpP1 protein in wild-type and transplastomic plants. Total soluble protein extracts of the wild type and three independently generated transplastomic lines per construct were subjected to western blotting with a ClpP1-specific antibody. The Coomassie-stained high molecular weight region of the blotted gel is shown as a control for equal loading below the blot.

To investigate the consequences of the mutated start codons, the expression of *clpP* was analyzed at the mRNA and protein levels. As *clpP* is part of an operon and, moreover, contains two introns, its transcript pattern is rather complex (Sugita and Sugiura, 1996; Kuroda and Maliga, 2002). Integration of the *aadA* cassette between the promoters P*clpP-53* and P*clpP-95* (Fig. 3A) had no effect on the pattern of the major *clpP* transcripts (Supplementary Fig. S2A). The unspliced transcript of 2.2 kb, the partially spliced transcripts of 1.5 kb (containing exon 1, intron 1, exon 2, and exon 3) and 1.35 kb (containing exon 1, exon 2, intron 2, and exon 3), and the mature 0.8 kb mRNA were present in all transplastomic plants (Supplementary Fig. S2A). Whereas the *clpP* mRNA accumulated to wild-type levels in Nt*clpP*_ATG_ and Nt*clpP*_GTG_ plants, enhanced accumulation of *clpP* transcripts and additional read-through transcripts were seen in Nt*clpP*_TTGs_ lines. The read-through transcripts are the result of inefficient transcription termination downstream of the P*rrn*-driven *aadA* marker gene (Supplementary Fig. S2A) (Zhou *et al.*, 2008; Apel and Bock, 2009; Oey *et al.*, 2009). To examine possible effects of the insertion of the *aadA* cassette on the neighboring *psbB* gene, northern blots with a *psbB*-specific probe were conducted. This probe detected the complex transcript pattern of the *psbB* operon comprising the genes *psbB*, *psbT*, *psbH*, *petB*, and *petD* (summarized in Krech *et al.*, 2013) in both the wild-type and the Nt*clpP*_TTGs_ plants (Supplementary Fig. S2B). In contrast, Nt*clpP*_ATG_ and Nt*clpP*_GTG_ plants showed enhanced accumulation of the 5.6 kb and 1.9 kb transcripts and three additional transcripts, most probably due to read-through transcription from the *aadA* cassette (Supplementary Fig. S2B). Since Nt*clpP*_ATG_ plants had no mutant phenotype (see below) (Fig. 4), we conclude that the changed transcript abundance does not influence accumulation of the PsbB protein. As a control, northern blots were also hybridized to a *psaB*-specific probe. The *psaB* gene (encoding a reaction center protein of PSI) is localized in a different region of the plastid genome. As expected, its large 5.2 kb transcript accumulates to wild-type levels in all transplastomic lines (Supplementary Fig. S2C).

Finally, the level of ClpP1 protein accumulation in the transplastomic plants was analyzed with an anti-ClpP1 antibody (Ostersetzer *et al.*, 1996). As expected, ClpP1 accumulated to wild-type levels in the *aadA* control line. In contrast, introduction of GUG or UUG as the translation initiation codon led to substantially reduced ClpP1 accumulation that was estimated to be ~50% (Nt*clpP*_GTG_) and ~30% (Nt*clpP*_TTGs_), respectively, of the wild-type level (Fig. 4D).

### *Phenotypes of transplastomic* clpP *mutants*

Next, we compared the phenotypes of transplastomic *clpP* mutants with wild-type plants under autotrophic growth conditions. The *aadA* control line Nt*clpP*_ATG_ (containing wild-type levels of ClpP1) (Fig. 4D) appeared wild-type-like throughout its entire life cycle (Fig. 4A, B). In contrast, the Nt*clpP*_GTG_ (~50% ClpP1 protein) and Nt*clpP*_TTGs_ (~30% ClpP1) lines displayed distinct phenotypes correlating with the level of ClpP1 accumulation (Fig. 4). Both were retarded in growth and showed pigment deficiencies. While the Nt*clpP*_GTG_ mutant displayed only subtle growth retardation and slightly reduced leaf pigmentation (and was nearly indistinguishable from the wild type later in development) (Fig. 4A), young leaves of Nt*clpP*_TTGs_ plants were yellowish and, even when mature, did not reach the pigmentation level of wild-type plants. Interestingly, the increased greening with time was not uniform across the entire leaf area, in that the plants developed slightly variegated leaves with yellowish and light green sectors. In addition, growth of Nt*clpP*_TTGs_ plants was substantially retarded and the mutants needed an additional 3 weeks to complete their life cycle (Fig. 4A, B).

### Down-regulation of nucleus-encoded subunits of the Clp protease core complex

**Fig. 5.**
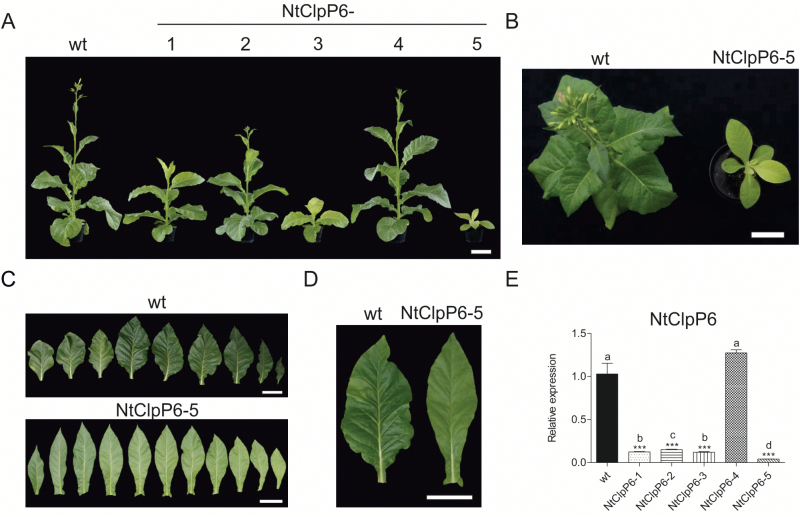
Effects of down-regulation of the CLPP6 subunit in tobacco. (A) Phenotypic comparison of a wild-type plant (wt) and five NtClpP6 RNAi lines from the T_2_ generation. Plants were raised from seeds and photographed after 9 weeks of growth in the greenhouse. (B) A wild-type plant and an NtClpP6-5 plant 9 weeks after sowing photographed from the top. (C) Detached leaves from a 10-week-old wild type and a 13-week-old NtClpP6-5 RNAi line. Shown is a series of all true leaves from the bottom (left) to the top (right) of the plant. (D) Comparison of leaf morphology between the wild type and the NtClpP6-5 RNAi line. Scale bars=10 cm. (E) *CLPP6* expression levels as quantified by qRT-PCR analysis. Columns and bars represent the means and SE (*n*=3), respectively. *UBC2* gene expression was used for normalization. Asterisks and letters indicate significant differences between transgenic and wild-type plants. ANOVA (*P*<0.05) and Tukey’s post-test were performed for all lines (***P<0.0001).

**Fig. 6.**
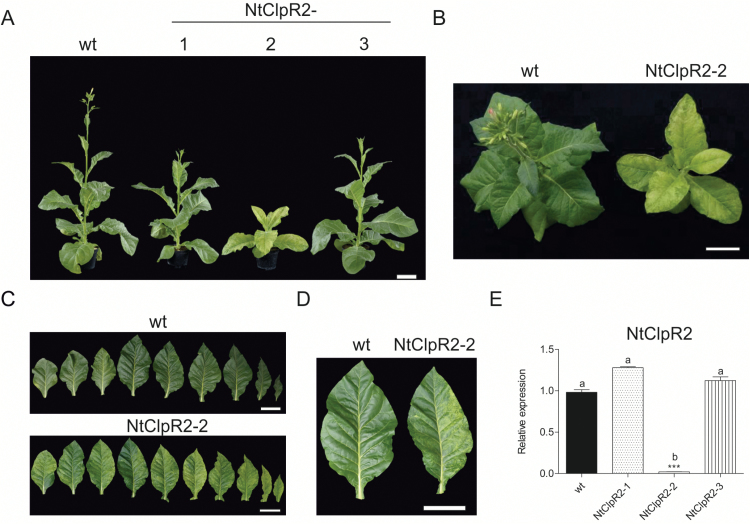
Effects of down-regulation of the CLPR2 protease subunit in tobacco. (A) Phenotypic comparison of a wild-type plant (wt) and three NtClpR2 RNAi lines from the T_2_ generation. Plants were raised from seeds and photographed after 9 weeks of growth in the greenhouse. (B) Wild-type and NtClpR2-2 plants 9 weeks after sowing. (C) Detached leaves from a 10-week-old wild type and an 11-week-old NtClpR2-2 RNAi line. Shown is a series of all true leaves from the bottom (left) to the top (right) of the plant. (D) Comparison of leaf morphology between the wild type and the NtClpR2-2 RNAi line. Scale bars=10 cm. (E) *CLPR2* expression levels quantified by qRT-PCR analysis. Columns and bars represent the means and SE (*n*=3), respectively. *UBC2* gene expression was used for normalization. Asterisks and letters indicate significant differences between transgenic and wild-type plants. ANOVA (*P*<0.05) and Tukey’s post-test were performed for all lines (***P<0.0001).

To test the effects of down-regulated expression of subunits forming the two core rings of the Clp protease, the *CLPP6* and *CLPR2* genes were targeted by RNAi. The CLPP6 protein represents a catalytic subunit and is specific to the P-ring, whereas CLPR2 is a non-catalytic subunit specific to the R-ring. Using *Agrobacterium*-mediated transformation, 20 independent nuclear-transgenic lines were generated for each subunit. From these, 3–5 lines covering strong, mild, and wild-type-like phenotypes were selected for further analysis (Figs 5, 6).

The majority of the NtClpP6 RNAi lines displayed pale and narrow leaves. The remaining lines showed either variegated leaves or a wild-type-like phenotype. These phenotypes were observed upon both heterotrophic growth on sucrose-containing medium and autotrophic growth in soil. Also, plant growth was retarded compared with the wild type (Fig. 5). Nonetheless, all RNAi lines were able to complete their life cycle and produce seeds. Analysis of the T_1_ and T_2_ generations confirmed the stability of the RNAi effect as evidenced by persistence of the phenotypes. From the five independent lines characterized in detail, one represented a mild RNAi line that showed a weak variegated phenotype (NtClpP6-2) and three were strong RNAi lines (NtClpP6-1, NtClpP6-3, and NtClpP6-5) showing a variegated phenotype or a pale phenotype and narrow leaves. One line showed a wild-type-like phenotype (NtClpP6-4), suggesting little if any gene silencing (Fig. 5A, B). Quantification of *CLPP6* mRNA levels by qRT-PCR revealed the expected correlation between the severity of the phenotype and the reduction in *CLPP6* expression. The strongest RNAi line (NtClpP6-5) showed the strongest reduction in *CLPP6* transcript levels (4% of the wild type), followed by the NtClpP6-3 (11%), NtClpP6-1 (13%), and NtClpP6-2 (15%) RNAi lines with milder phenotypes and the NtClpP6-4 RNAi line that showed no reduction in transcript levels (Fig. 5E) and, accordingly, a wild-type-like phenotype. Especially in the strongest RNAi line NtClpP6-5, plant development and growth were severely affected (Fig. 5A, B; Supplementary Fig. S3). Interestingly, the narrow leaf phenotype that was observed in the strongest RNAi line (NtClpP6-5) (Fig. 5C, D) had not been described for Arabidopsis *CLPP6* antisense plants (with ~90% reduction in transcript and protein levels) (Sjögren *et al.*, 2006).

The majority (~75%) of the NtClpR2 RNAi lines displayed a variegated leaf phenotype, whereas the rest showed a wild-type-like phenotype. From the three lines transferred to soil, one retained the variegated leaf phenotype (NtClpR2-2), whereas the other two showed a wild-type-like appearance (Fig. 6A). The severe phenotype of line NtClpR2-2 correlates with a very strong reduction in *CLPR2* transcript levels (to 3% of the wild-type level) (Fig. 6E). All RNAi lines produced seeds, and their phenotypes were stable in the T_1_ and T_2_ generations. The variegated phenotype was clearly different from the pale leaf phenotype previously reported in an Arabidopsis T-DNA mutant for *CLPR2* (Kim *et al.*, 2013). The exact reason for this phenotypic difference remains to be determined, but, since variegation phenotypes are commonly associated with threshold effects and stochasticity in gene expression, it seems possible that the level of residual *CLPR2* expression in our tobacco knock-down lines is very close to the critical level required for normal chloroplast biogenesis. The NtClpR2-2 RNAi line also showed a reduced size and slow growth rate compared with wild-type plants (Fig. 6A–D; Supplementary Fig. S3). Together, the observed severe phenotypes of strong NtClpP6 and NtClpR2 RNAi lines suggest that the Clp protease core complex plays important roles in photosynthesis, plant growth, and development.

### *Down-regulation of the* CLPC *subunit of the chaperone ring*

**Fig. 7.**
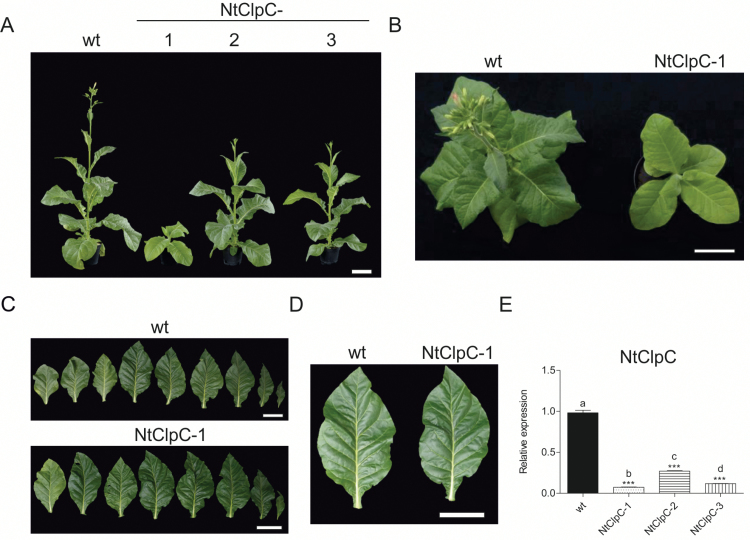
Effects of down-regulation of the CLPC subunit. (A) Phenotypic comparison of a wild-type plant (wt) and three independent NtClpC RNAi lines from the T_2_ generation. Plants were raised from seeds and photographed after 9 weeks of growth in the greenhouse. (B) Wild-type and NtClpC-1 plants 9 weeks after sowing. (C) Detached leaves from a 10-week-old wild type and a 12-week-old NtClpC-1 RNAi line. (D) Leaf comparison between the wild type and the NtClpC-1 RNAi line. Scale bars=10 cm. (E) *CLPC* expression levels quantified by qRT-PCR analysis. Columns and bars represent the means and SE (*n*=3), respectively. *UBC2* gene expression was used for normalization. Asterisks and letters indicate significant differences between transgenic and wild-type plants. ANOVA (*P*<0.05) and Tukey’s post-test were performed for all lines (***P<0.0001).

A *clpc1* null mutant in Arabidopsis was reported to display a pale phenotype, and *clpc1/clpc2* double mutants are blocked in embryogenesis (Kovacheva *et al.*, 2007). Tobacco NtClpC RNAi lines (where both parental alleles were targeted) growing in tissue culture did not display any conspicuous phenotype. When transferred to soil, plants developed pale green phenotypes within a few weeks which, however, became less severe as the plants grew older. When raised from seeds, NtClpC-2 and NtClpC-3 plants showed a normal phenotype and differed from the wild type only by their slightly reduced height (Fig. 7A). In contrast, the NtClpC-1 line showed a pale green phenotype and a strong delay in growth and development (Fig. 7A–D; Table 1; Supplementary Figs S3, 4). This phenotype resembles that described for a T-DNA insertion mutant in Arabidopsis (Kovacheva *et al.*, 2007). In line with the observed phenotypes, *CLPC* transcript levels in the tobacco RNAi lines were strongly decreased in NtClpC-1 (to 7% of the wild-type level), while mRNA levels in the NtClpC-2 (27%) and NtClpC-3 (13%) RNAi lines were less severely reduced (Fig. 7E). Interestingly, after 12 weeks of growth in soil, leaves of the NtClpC-1 line regreened (Fig. 7C, D) and the plants became more similar to the wild type. This observation suggests that, in contrast to the other subunits of the complex analyzed here, CLPC is particularly important during the early stages of plant development. The limited phenotypic impact later in development could reflect a reduced need for Hsp100-like chaperone activity in the chloroplast and/or partial compensation by related chaperones (e.g. ClpD).

**Table 1.** Effects of Clp and FtsH subunit silencing on plant growth, development, and morphology See also Supplementary Fig. S4.

**Knock-down line**	**Leaf shape**	**Leaf pigmentation**	**Leaf size**	**Plant height**	**Plant growth**	**Plant development**
Nt*clpP*_ATG_	Normal	Normal	Normal	Normal	Normal	Normal
Nt*clpP*_GTG_	Normal	Pale^*a*^	Normal	Normal	Normal	Normal
Nt*clpP*_TTGs_	Normal	Pale^*b*^	Reduced^*c*^	Reduced^*c*^	Affected	Retarded^*d*^
NtClpP6	Narrow	Pale^*e*^	Reduced	Reduced	Affected	Retarded
NtClpR2	Normal	Variegated	Reduced^*f*^	Reduced	Affected	Retarded
NtClpC	Normal	Pale^*g*^	Reduced^*g*^	Normal	Affected^*g*^	Retarded^*g*^
NtClpS	Normal	Normal	Normal	Normal	Normal	Normal
NtClpT1-T2	Normal	Normal	Normal^*h*^	Normal	Normal^*i*^	Retarded^*j*^
NtFtsH	Normal	Variegated	Reduced	Reduced	Affected	Retarded

^*a*^ Until plants reached the age of 5 weeks.

^*b*^ Pale yellow young leaves.

^*c*^ Slightly reduced.

^*d*^ Until plants reached the age of 10 weeks.

^*e*^ Pale yellow leaves.

^*f*^ Until plants reached the age of 12 weeks.

^*g*^ Until plants reached the age of 12 weeks.

^*h*^ Normal after 3 weeks of growth.

^*i*^ Normal after 5 weeks of growth.

^*j*^ Until plants reached the age of 9 weeks.

### Knock-down of the adaptor protein CLPS and the accessory proteins CLPT1 and CLPT2

**Fig. 8.**
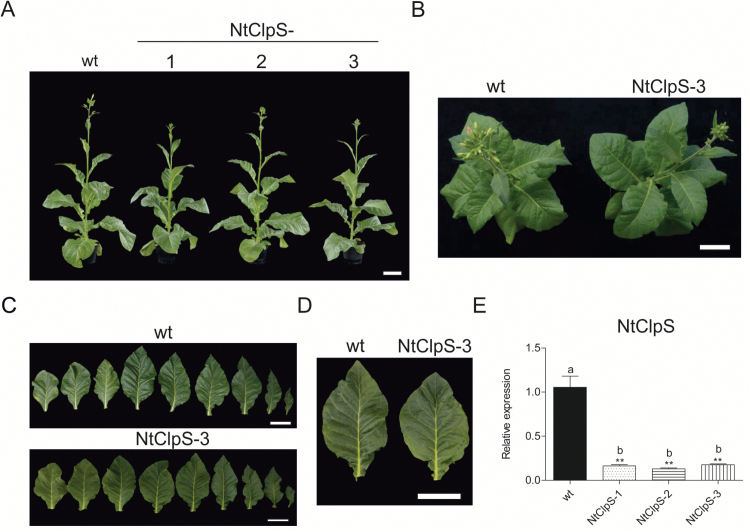
Effect of down-regulation of the CLPS subunit. (A) Phenotypic comparison of a wild-type plant (wt) and three independent NtClpS RNAi lines from the T_2_ generation. Plants were raised from seeds and photographed after 9 weeks of growth in the greenhouse. (B) Wild-type and NtClpS-3 plants 9 weeks after sowing. (C) Detached leaves from a 10-week-old wild-type and an NtClpS-3 RNAi plant of the same age. (D) Leaf comparison between the wild type and the NtClpS-3 RNAi line. Scale bars=10 cm. (E) *CLPS* expression levels quantified by qRT-PCR analysis. Columns and bars represent the means and SE (*n*=3), respectively. *UBC2* gene expression was used for normalization. Asterisks and letters indicate significant differences between transgenic and wild-type plants. ANOVA (*P<*0.05) and Tukey’s post-test were performed for all lines (**P<0.005).

RNAi lines generated against the *CLPS* gene were indistinguishable from the wild type, even though they showed a strong reduction in Nt*CLPS* transcript levels (Fig. 8). Under a variety of growth conditions tested, no aberrant phenotype was observed, with the exception of a very subtle growth retardation that was occasionally seen (Fig. 8A; Table 1; Supplementary Figs S3, S4). This finding is consistent with the wild-type-like phenotype of an Arabidopsis *clps* null mutant (Nishimura *et al.*, 2013).

**Fig. 9.**
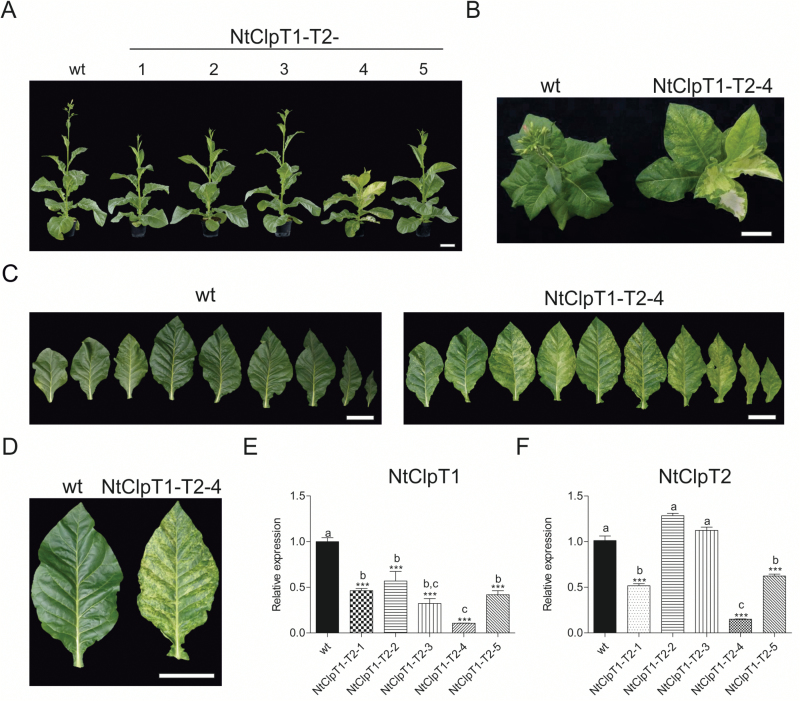
Effect of down-regulation of the CLPT1 and CLPT2 subunits. (A) Phenotypic comparison of a wild-type plant (wt) and five independent NtClpT1-T2 RNAi lines from the T_2_ generation. Plants were raised from seeds and photographed after 9 weeks of growth in the greenhouse. (B) Wild-type and NtClpT1-T2-4 plants 9 weeks after sowing. (C) Detached leaves from a 10-week-old wild type and a similarly old NtClpT1-T2-4 RNAi plant. (D) Comparison of leaf morphology between the wild type and the NtClpT1-T2-4 RNAi line. Scale bars=10 cm. (E) *CLPT1* and *CLPT2* expression levels quantified by qRT-PCR analysis. Columns and bars represent the means and SE (*n*=3), respectively. *UBC2* gene expression was used for normalization. Asterisks and letters indicate significant differences between transgenic and wild-type plants. ANOVA (*P*<0.05) and Tukey’s post-test were performed for all lines (***P<0.0001).

Expression of the *CLPT1* and *CLPT2* genes was reduced by a transgenic co-silencing strategy (see the Materials and methods). A large fraction of RNAi lines showed a variegated leaf phenotype (~60% of the plants) already upon growth in tissue culture. When grown in soil, RNAi line NtClpT1-T2-4 showed a strong variegated phenotype (Fig. 9C, D), growth retardation, and a delay in plant development (Fig. 9A; Table 1B; Supplementary Figs S3, S4). These severe phenotypes correlated with the strength of the RNAi silencing in that mRNA levels were reduced to 15% of the wild-type level in line NtClpT1-T2-4. Lines NtClpT1-T2-1 and NtClpT1-T2-5 showed milder phenotypes consistent with a less severe reduction in transcript levels to 52% and 62%, respectively. Lines NtClpT1-T2-2 and NtClpT1-T2-3 had no reduction in transcript levels, consistent with their wild-type-like phenotype (Fig. 9). These findings support an important function for the CLPT1 and CLPT2 proteins in growth and development, as recently also revealed by generation of a *clpt1/clpt2* double mutant in Arabidopsis (Kim *et al.*, 2015). However, the pale green phenotype observed in the Arabidopsis double mutants differs from the variegated leaf phenotype displayed by our strong RNAi lines in tobacco.

### Knock-down of the FTSH1-5 protease in the thylakoid membrane

**Fig. 10.**
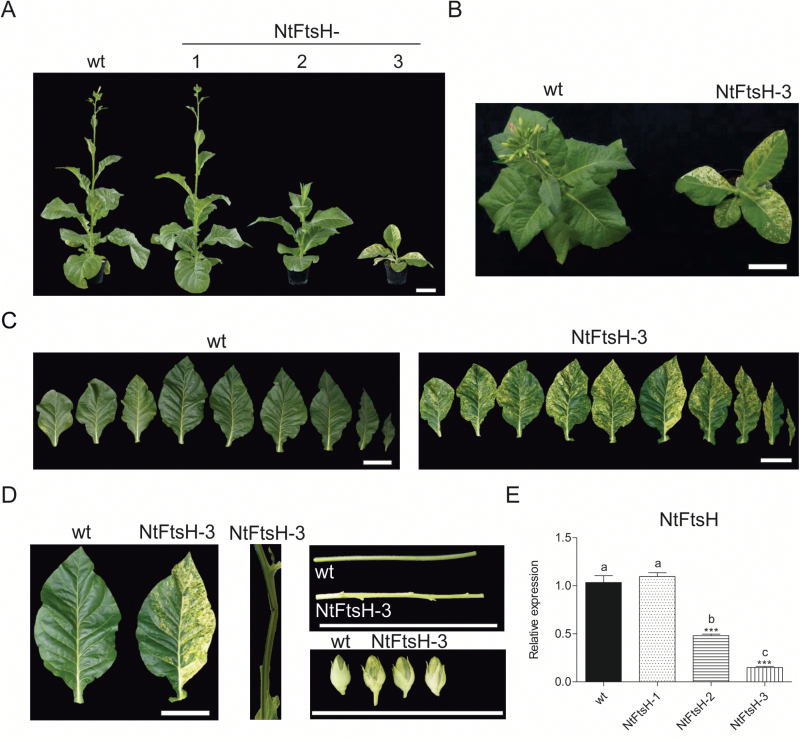
Effect of down-regulation of the FTSH1-5 subunit in tobacco. (A) Phenotypic comparison of a wild-type plant (wt) and three independent NtFtsH RNAi lines from the T_2_ generation. Plants were raised from seeds and photographed after 9 weeks of growth in the greenhouse. (B) Wild-type and NtFtsH plants 9 weeks after sowing. (C) Detached leaves from a 13-week-old wild type and a similarly old NtFtsH-3 RNAi plant. (D) Comparison of leaves, stems, and capsules between the wild type and the NtFtsH-3 RNAi line. Scale bars=10 cm. (E) *FTSH* expression levels quantified by qRT-PCR analysis. Columns and bars represent the means and SE (*n*=3), respectively. *UBC2* gene expression was used for normalization. Asterisks and letters indicate significant differences between transgenic and wild-type plants. ANOVA (*P*<0.05) and Tukey’s post-test were performed for all lines (***P<0.0001).

The FtsH protease subunit FTSH1/FTSH5 was selected as a control target for gene silencing by RNAi because of its well-studied role in the degradation of the D1 protein of PSII in the thylakoid membrane (Lindahl *et al.*, 2000; Sakamoto *et al.*, 2003; Zaltsman *et al.*, 2005*a*). Consistent with its membrane association, it is believed to be mainly responsible for the turnover of thylakoid membrane proteins. However, degradation of some stromal proteins by the FtsH protease cannot be excluded. While Arabidopsis has two closely related genes encoding this subunit, *FTSH1* and *FTSH5*, Solanaceous plants have only one gene for this FtsH family member (Supplementary Fig. S1; Supplementary Table S1) which we tentatively named F*TSH1-5* to indicate its relatedness to both Arabidopsis genes. Tobacco FtsH1-5 RNAi lines (subsequently referred to NtFtsH) displayed phenotypes ranging from pale-variegated and green-variegated plants to wild-type-like plants (Fig. 10; Supplementary Fig. S3). Similar variegated phenotypes were described for FtsH protease mutants in Arabidopsis and tobacco (Sakamoto *et al.*, 2002; Zhang *et al.*, 2010; Kato *et al.*, 2012). The severity of the phenotypes of our RNAi lines correlated well with the reduction in *FTSH* transcript levels, which was strongest in line NtFtsH-3 (15% of the wild-type level), followed by NtFtsH-2 (45%) and NtFtsH-1 that showed no reduction in transcript levels (Fig. 10). Similarly, growth retardation and delayed development in the knock-down lines correlated with the intensity of the RNAi effect (Fig. 10; Table 1; Supplementary Figs S3, S4). In addition to the strong leaf variegation seen in line NtFtsH-3, variegation was also observed in stems, lateral branches, and capsules (Fig. 10D).

### Photosynthetic activity in tobacco Clp and FtsH knock-down lines

**Fig. 11.**
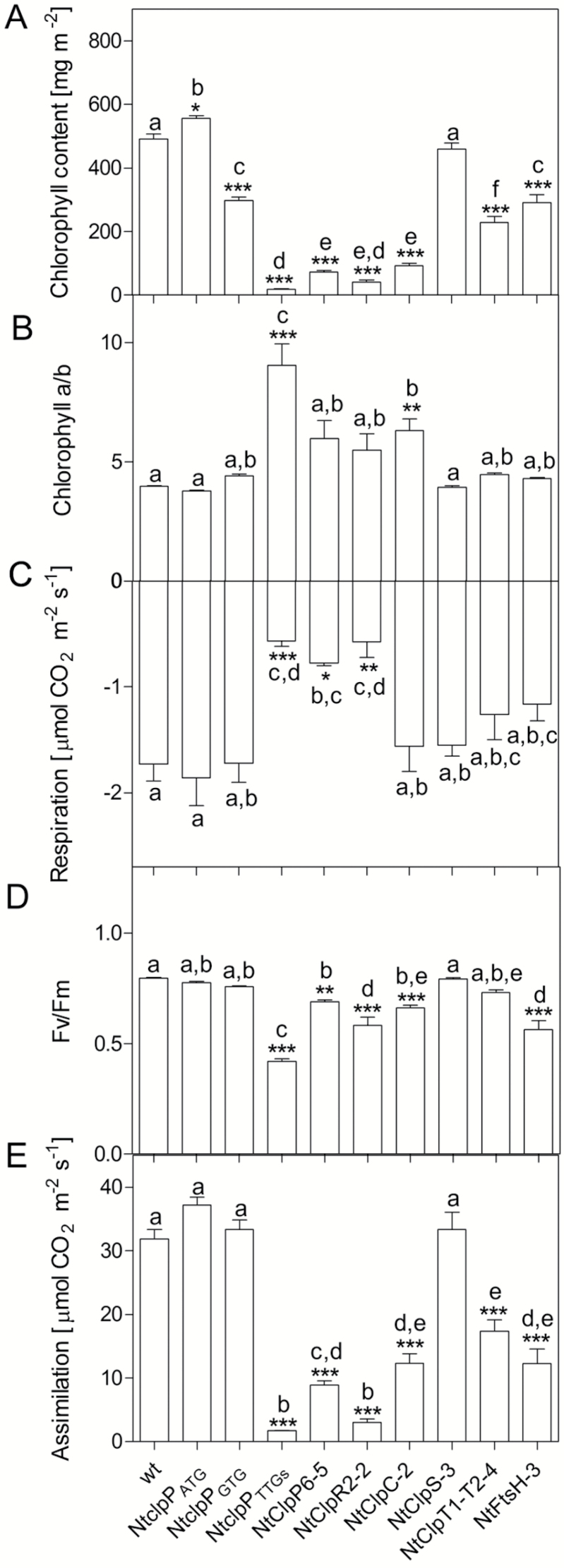
Physiological parameters measured in wild-type tobacco (wt) and the Clp and FtsH knock-down lines generated in this study. (A) Chlorophyll content. (B) Chl *a*/*b* ratio. (C) Respiration. (D) Maximum quantum efficiency of PSII (*F*_v_/*F*_m_). (E) Leaf assimilation. Columns and bars represent the means and SE of at least four biological replicates (*n*=4). Asterisks and letters indicate significant differences between transgenic and wild-type plants. ANOVA and Tukey’s post-test were performed for all lines (*P<0.05; **P<0.005; ***P<0.0001).

Having observed pigment-deficient phenotypes in knock-down lines of several of the studied protease subunits, we wanted to examine the effects of impaired protease function on photosynthesis in more detail. To this end, a number of parameters related to photosynthetic activity were determined in the strongest RNAi line for each protease subunit (Fig. 11). Leaf chlorophyll content was reduced in all lines, with the exception of NtClpS-3 and Nt*clpP*_ATG_ (Fig. 11A). Severely affected lines were NtClpP6-5, NtClpR2-2, NtClpC-2, and Nt*clpP*_TTGs_ with 15, 8, 19, and 4% of the wild-type levels, respectively (Fig. 11A). Interestingly, the three lines with the strongest reduction in chlorophyll content represent subunits of the Clp core complex (closely followed by the NtClpC-2 line). The Chl *a*/*b* ratio was significantly increased in the NtClpC-2 and Nt*clpP*_TTGs_ lines, indicating a strong reduction in light-harvesting complexes (LHCs; which bind Chl *a* and *b*), relative to the photosynthetic reaction centers (which only bind Chl *a*) (Fig. 11B). The increases in lines NtClpP6-5 and NtClpR2-2 were not statistically significant. Leaf respiration was reduced in NtClpP6-5, NtClpR2-2, and Nt*clpP*_TTGs_ to 45, 33, and 33%, respectively, while the other lines were not significantly affected (Fig. 11C). Leaf assimilation remained unaffected in NtClpS-3, Nt*clpP*_ATG_, and Nt*clpP*_GTG_ (Fig. 11E). The NtClpT1-T2-4, NtClpC-2, and NtFtsH-3 lines showed decreases to 54, 39, and 38%, respectively (Fig. 11E). Again, the most strongly affected lines were those in which subunits of the Clp core complex were knocked down (NtClpP6, NtClpR2-2, and Nt*clpP*_TTGs_, with decreases to 28, 9, and 5% of the wild-type levels) (Fig. 11E), and which had shown the most severe repression of dark respiration. The chlorophyll fluorescence parameter *F*_v_/*F*_m_, a measure of the maximum quantum efficiency of PSII photochemistry, remained unchanged in NtClpS-3, the transplastomic *aadA* control line Nt*clpP*_ATG_, and the Nt*clpP*_GTG_ line, while NtClpP6-5, NtClpR2-2, NtClpC-2, NtClpT1-T2-4, and NtFtsH-3 showed clear reductions (Fig. 11D). The transplastomic mutant Nt*clpP*_TTGs_ showed the strongest reduction in *F*_v_/*F*_m_ (to 52% of the wild-type levels) (Fig. 11D). Taken together, the physiological data obtained explain the observed visual phenotypes of the knock-down lines and their delay in growth and development. However, the data also reveal that the effects of the different gene knock-downs on the different physiological parameters measured can be distinct. For example, while Nt*clpP*_GTG_ and NtFtsH-3 plants have similar chlorophyll contents and Chl *a*/*b* ratios, the NtFtsH-3 plants are much more strongly affected in leaf assimilation than the Nt*clpP*_GTG_ plants. A possible reason could be that photosynthetic electron transport and assimilation are directly limited by PSII photoinhibition in the NtFtsH-3 plants, as evidenced by the strongly decreased *F*_v_/*F*_m_ of the knock-down line. Alternatively, impaired function of the FtsH protease might affect the accumulation and/or function of the cytochrome *b*_6_*f* complex or chloroplast ATP synthase, which together control photosynthetic electron transport in tobacco (Rott *et al.*, 2011; Schöttler *et al.*, 2015).

### Specific effects of protease gene knock-downs on the expression of nucleus-encoded Clp subunits

**Fig. 12.**
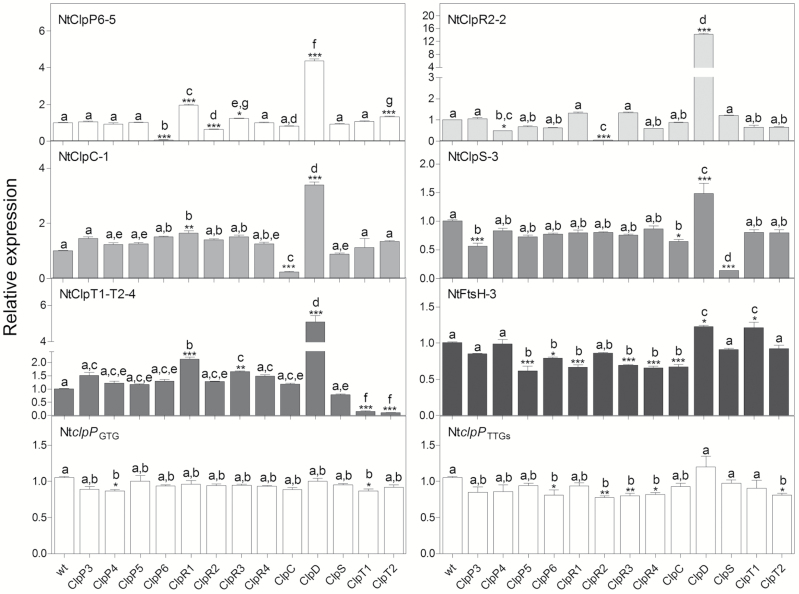
Changes in the expression levels of nuclear-encoded Clp subunits. The expression levels of all Clp subunits were quantified (by qRT-PCR) in the strongest NtClpP6-5, NtClpR2-2, NtClpC-1, NtClpS-3, NtClpT1-T2-4, and NtFtsH-3 RNAi lines and the transplastomic Nt*clpP*_GTG_ and Nt*clpP*_TTGs_ lines. Columns and bars represent the means and SE (*n*=3). *UBC2* gene expression was used for normalization. Asterisks and letters indicate significant differences between transgenic and wild-type plants (wt). ANOVA and Tukey’s post-test were performed for all lines (*P<0.05; **P<0.005; ***P<0.0001).

To gain more information about the regulation of the Clp complex and its assembly, the transcript levels of all nuclear genes encoding Clp subunits were measured in the two transplastomic mutants and in the strongest RNAi lines of each Clp and FtsH subunit targeted in this study (Fig. 12). In the strongest NtClpP6-5 line, an increase in expression levels of *CLPR1*, *CLPR3*, *CLPD*, and *CLPT2* was observed. Interestingly, the gene encoding the chaperone subunit CLPD responded most sensitively to the CLPP6 deficiency by showing a 400% increase in expression (Fig. 12). In addition, a reduction in *CLPR2* expression (to 64% of the wild-type level) was observed. In the NtClpR2-2 line, the *CLPD* genes showed an even stronger increase in expression (1300%), while a significant reduction was observed for *CLPP4* (to 49%) (Fig. 12). In the NtClpC-1 line, increased expression of *CLPR1* and again *CLPD* was observed. In the NtClpS-3 line, expression levels of *CLPP3* and *CLPC* were found to be reduced, and ClpD expression was increased, albeit much less strongly so than in the P-ring and R-ring knock-down lines. In the strongest RNAi line for the two CLPT accessory proteins, NtClpT1-T2-4, elevated expression levels of *CLPR1*, *CLPR3*, and again the chaperone subunit *CLPD* were measured. In the RNAi line for the thylakoid membrane protease, NtFtsH1-5, significant increases and decreases in expression levels of several Clp subunits were seen, but they were overall rather small (Fig. 12). In the weaker transplastomic line, Nt*clpP*_GTG_, most of the nuclear *CLP* genes remained unaffected by the reduced expression of *clpP*, and only in two of them (*CLPP4* and *CLPT1*) did a small but significant reduction in transcript levels occur. In the stronger transplastomic line, Nt*clpP*_TTGs_, we observed a similar pattern, with small reductions in the expression levels of the *CPP6*, *CLPR2*, *CLPR3*, *CLPR4*, and *CLPT2* genes (Fig. 12).

In overview, down-regulation of individual protease subunits causes specific signatures in the expression patterns of nuclear genes for chloroplast protease subunits, raising the interesting possibility that these signatures represent compensatory responses to the specific subunit deficiency in each knock-down line.

## Discussion

Thus far, the Clp protease has been mainly studied in *Arabidopsis thaliana*, and our current knowledge about its structure and functions in other plants is very limited. In tobacco, essentiality of the plastid-encoded ClpP1 subunit was reported (Shikanai *et al.*, 2001; Kuroda and Maliga, 2003), but only one nucleus-encoded subunit (CLPP4; Wei *et al.*, 2015) was partially characterized. The lack of a properly annotated tobacco genome has made it difficult to identify genes and gene families in this model plant. Here, a combination of bioinformatic tools was used to identify the genes for Clp and FtsH subunits encoded in the nuclear genome (Fig. 2; Supplementary Fig. S1; Supplementary Table S5). While the Clp protease is located in the stroma (Sjögren *et al.*, 2004, 2006; Kim *et al.*, 2013, 2015; Nishimura *et al.*, 2013), the FTSH1-5 subunit resides in the thylakoid membrane (Zhang *et al.*, 2010). In Arabidopsis, the FtsH family comprises four chloroplast-located subunits (isoforms): FTSH1, FTSH2, FTSH5, and FTSH8. FTSH1 and FTSH5 (also termed subunit type A) and FTSH2 and FTSH8 (subunit type B) are redundant and arose through gene duplication events (Yu *et al.*, 2004; Zaltsman *et al.*, 2005*a*). In contrast, the tobacco and tomato genomes contain only a single gene for FTSH1 and FTSH5 (here referred to as FTSH1-5) and a single gene for FtsH2 and FtsH8 (FTSH2_8) (Supplementary Fig. S1), lending support to the proposed functional redundancy of the duplicated Arabidopsis genes.

The core complex of the Clp protease consists of nine subunits, forming a two-ring structure (Fig. 1). The plastid-encoded ClpP1 is the only subunit in the R-ring that has proteolytic activity. Taking into account the functional data previously obtained in Arabidopsis, successful suppression of expression of one subunit of the P-ring (ClpP6) and two subunits of the R-ring (ClpR2 and ClpP1) allowed us to infer from the observed phenotypes the effects of impaired formation of the core complex. Interestingly, suppression of the three subunits had distinct phenotypic effects. While strong NtClpP6 RNAi lines and the strong transplastomic ClpP1 mutant Nt*clpP*_TTGs_ exhibited severe pale phenotypes (Figs 4, 5), the strongest NtClpR2 RNAi line showed a variegated phenotype (Fig. 6; Supplementary Fig. S3). Similar leaf variegations had not been seen in an Arabidopsis mutant with a 20% residual *CLPR2* transcript level (Rudella *et al.*, 2006; Kim *et al.*, 2009; Zybailov *et al.*, 2009). Also, Arabidopsis *clpr2* null mutants growing on sucrose-containing medium showed a strong pale leaf phenotype with serrated leaves (Kim *et al.*, 2009), but no leaf variegations. Tobacco NtClpP6 RNAi lines resembled the phenotypes observed in Arabidopsis and rice *clpp6* mutants (Sjögren *et al.*, 2006; Dong *et al.*, 2013). The pale young leaves of the rice and Arabidopsis mutants gradually became greener during development, a phenomenon also seen in our tobacco RNAi lines, although the older leaves did not recover to wild-type pigmentation levels (Fig. 5). Alleviation of the phenotype with progressing development suggests a greater importance of the Clp activity during early leaf growth (Sjögren *et al.*, 2006), or, alternatively, a low turnover of the Clp complex (resulting in increased accumulation over time in the knock-down lines).

NtClpP6 RNAi lines were strongly affected in development (Supplementary Fig. S4A), whereas NtClpR2 RNAi lines were much less affected. The most striking phenotypic difference between the two RNAi lines was that the strongest CLPP6 RNAi line (Fig. 5C, D) displayed striking alterations in leaf morphology. A similar narrow-leaf phenotype was described in transplastomic tobacco mutants with reduced activity of plastid translation (Fleischmann *et al.*, 2011; Ehrnthaler *et al.*, 2014; Tiller and Bock, 2014). Leaf shape especially at the leaf margin was also affected in Arabidopsis *CLPP6* antisense mutants (Sjögren *et al.*, 2006). Whether the effects of CLPP6 knock-down on leaf morphology are mediated by down-regulated chloroplast translational activity in our NtClpP6 RNAi lines will be interesting to investigate. Also, why leaf shape is more strongly affected in some Clp knock-down lines than in others (Table 1) needs to be examined in future studies. Since none of the ~130 plastid-encoded genes is directly involved in leaf development (Scharff and Bock, 2014), it can be assumed that the effects of plastid translation and protein homeostasis on leaf morphology are the result of retrograde signaling (Tiller and Bock, 2014).

ClpP1 is encoded by the plastid gene *clpP*, and earlier gene disruption studies in *N. tabacum* revealed its essentiality (evidenced by the failure to isolate homoplasmic transplastomic knock-out lines; Shikanai *et al.*, 2001; Kuroda and Maliga, 2003). Since chloroplast transformation technology is not available for Arabidopsis, tobacco is currently the most suitable model to study ClpP1 function. Here, we obtained the first homoplasmic transplastomic ClpP1 mutants in seed plants. By introducing the less efficient initiation codons GUG and UUG, we created tobacco plants with reduced ClpP1 expression. Using a similar approach in the unicellular green alga *C. reinhardtii* (Majeran *et al.*, 2000), ClpP1 accumulation could be reduced to ~15–45%. While our transplastomic tobacco mutants Nt*clpP*_GTG_ (~50% of ClpP1) and Nt*clpP*_TTGs_ (~30% of ClpP1) showed reduced growth rates and pigment deficiencies, the algal *clpP* mutant strain was unaffected under standard growth conditions. However, adaptation to elevated CO_2_ levels was impaired and nitrogen starvation affected accumulation of the cytochrome *b*_6_*f* complex (Majeran *et al.*, 2000). A recent study reported more efficient (conditional) repression of *clpP* in *Chlamydomonas* which led to arrested growth, disturbed plastid protein homeostasis, and, interestingly, elicited an autophagy-like response (Ramundo *et al.*, 2014).

Another interesting difference that becomes apparent upon comparison of the three core complex RNAi lines (CLPP6, CLPR2, and ClpP1) lies in the Chl *a*/*b* ratio. Although the strong RNAi lines of all three subunits show reduced chlorophyll contents per leaf area, only the ClpP1 knock-down line Nt*clpP*_TTGs_ has a strongly elevated Chl *a*/*b* ratio, indicating that accumulation of the (mainly Chl *b*-binding) light-harvesting antenna is more severely affected than that of the reaction centers (Fig. 11A, B). This is surprising, because most of the core subunits are encoded in the plastid genome, whereas all LHC proteins are encoded in the nucleus. Whether or not the down-regulated expression of the plastid ClpP1 protein elicits a retrograde signaling response that results in down-regulated LHC gene expression in the nucleus (Pogson *et al.* 2008) will be interesting to explore.

In Arabidopsis, the two CLPC proteins of the chaperone ring are 90% identical and have partially overlapping functions. A *clpc1* T-DNA knockout mutant showed a pale green phenotype, whereas a *clpc2* knockout was indistinguishable from the wild type (Kovacheva *et al.*, 2005, 2007). The *clpc1/clpc2* double mutant displayed embryo lethality, suggesting that expression of at least one *CLPC* gene is required (Sjögren et al., 2004; Kovacheva *et al.*, 2007). In the tobacco genome, we identified only one copy of *CLPC* (see above). Only the strongest NtClpC line was affected in leaf pigmentation and substantially delayed in growth and development (Fig. 7; Table 1; Supplementary Figs S3, S4). In addition to its function in protein degradation, CLPC also plays a role in protein import. Recently, association of CLPC1 with the import apparatus in the inner envelope membrane was demonstrated, and evidence for a role for the Clp protease during or immediately following the import of nucleus-encoded proteins into plastids was obtained (Constan *et al.*, 2004; Sjögren *et al.*, 2004; Kovacheva *et al.*, 2005, 2007; Flores-Perez *et al.*, 2016). The NtClpC tobacco RNAi lines generated here will be useful to dissect further the functions of the CLPC chaperone in post-import protein folding and protein degradation.

The adaptor protein CLPS is one of two proteins involved in substrate recognition by the Clp protease in Arabidopsis (Nishimura *et al.*, 2013, 2015). Although we achieved a strong knock-down in tobacco, all RNAi lines showed a wild-type-like phenotype (Figs 8, 11; Table 1; Supplementary Figs S3, S4). This is in agreement with previous reports that an Arabidopsis *clps* null mutant (Nishimura *et al.*, 2013) and a *Δclps* mutant in *E. coli* (Erbse *et al.*, 2006) did not show discernible growth phenotypes. In bacteria, the ClpA protein mediates recognition of N-end rule substrates by the Clp protease, raising the possibility that ClpS is not the only adaptor protein (Wang *et al.*, 2007). A new Clp adaptor, CLPF, was recently discovered in Arabidopsis chloroplasts (Nishimura *et al.*, 2015). ClpF and ClpS were proposed to play partially overlapping roles. However, the *clps*/*clpf* double mutant still showed no strong phenotype (Nishimura *et al.*, 2015), possibly suggesting an even greater redundancy in the Clp adaptor proteins.

The CLPT1 and CLPT2 proteins are unique in land plants and are believed to stabilize the Clp core complex (Kim *et al.*, 2015). A *clpt1* knock-down mutant and a *clpt2* knock-out in Arabidopsis showed wild-type-like phenotypes, while a recently generated *clpt1*/*clpt2* double mutant (Kim *et al.*, 2015) displayed a strong pale phenotype, delayed growth, and altered leaf shape, with the leaf margins being more serrated (Kim *et al.*, 2015), a phenotype that had been seen before in several other Arabidopsis mutants affected in plastid gene expression (Hricova *et al.*, 2006; Wang *et al.*, 2010; Lee *et al.*, 2013). In contrast, NtClpT1-T2 RNAi lines in tobacco displayed a variegated phenotype (Fig. 9), somewhat similar to the variegated phenotype observed in the NtClpR2 RNAi lines. Interestingly, in Arabidopsis, neither the *clpr2* nor the *clpt1/clpt2* mutants showed leaf variegations but rather a strong (but homogeneous) pale green phenotype. The reason for these differences between the two plant species and the molecular nature of the threshold events causing the variegations in tobacco should be further investigated.

FtsH, an ATP-dependent metalloprotease localized in thylakoid membranes, forms heteromeric complexes composed of FTSH1, FTSH5, FTSH2, and FTSH8 subunits. Lack of either *FTSH5* (*var1* mutant) or *FTSH2* (*var2*) causes variegated phenotypes in Arabidopsis (Chen *et al.*, 2000; Yu *et al.*, 2004; Zaltsman *et al.*, 2005*a*, *b*; Zhang *et al.*, 2010). Our tobacco RNAi lines also displayed variegated phenotypes (Fig. 10), probably reflecting the function of the FtsH protease in PSII repair by mediating degradation of photodamaged D1 protein (Zhang *et al.*, 2010; Malnoë *et al.*, 2014; Sacharz *et al.*, 2015). In Arabidopsis, several mutated genes were identified as suppressors of the variegation in *var1* and *var2* mutants (Liu *et al.*, 2010). All suppressor mutants appear to be impaired in chloroplast translation and, therefore, a model was proposed in which a disturbed equilibrium between synthesis and degradation of protein(s) required for chloroplast biogenesis causes the variegation phenotype (Albrecht *et al.*, 2006; Miura *et al.*, 2007; Yu *et al.*, 2008). Interestingly, one of the suppressors (*SVR2*) encodes the CLPR1 subunit of the Clp protease (Koussevitzky *et al.*, 2007). In addition, *clpc1*, *clpc2*, and *clpr4* mutants were reported as *var2* suppressors, suggesting a functional link between the Clp and FtsH proteases (Park and Rodermel, 2004; Yu *et al.*, 2008; Wu *et al.*, 2013). The unexpected variegation phenotype observed in our NtClpR2 and NtClpT1-T2 RNAi lines provides interesting material to elucidate the connection between plastid translation, protein degradation, and leaf variegations. Also, determination of protein levels, subunit turnover, and complex activity in the various knock-down lines would be useful to establish direct correlations between Clp function and the different phenotypes observed.

In summary, we have produced a set of Clp protease knock-down lines in tobacco that covers at least one component of each part of the complex (P-ring, R-ring, chaperone ring, adaptor, and accessory proteins). Moreover, it includes novel mutants for the plastid-encoded catalytically active subunit of the R-ring, ClpP1. The different knock-down lines show distinct phenotypic effects on plant growth and development, leaf morphology, pigmentation, and photosynthesis. The knock-down lines will provide a valuable resource to study chloroplast proteostasis and its regulation (De Marchis *et al.*, 2012) and to elucidate the role of the Clp protease in the various protein degradation pathways operating in chloroplasts, including the proposed N-end rule pathway (Apel *et al.*, 2010).

The knock-down lines in the collection proved to be phenotypically stable over (at least two) generations. All lines are available to the community upon request. The only requirement involved is a Material Transfer Agreement covering the selectable marker gene cassette used to generate the transplastomic *clpP1* mutants (to be concluded with Rutgers, The State University of New Jersey, USA).

In addition, the generated knock-down lines will allow us to determine if down-regulation of protease activities represents a suitable approach to increase the stability of recombinant proteins produced in the chloroplast. Tobacco is currently the only plant in which plastid transformation technology is routine, and tobacco is also the most preferred host plant in biotechnology (Maliga and Bock 2011; Tusé *et al.*, 2014; Bock, 2015). Protein stability has recently emerged as the factor that limits recombinant protein accumulation in most of the cases where plastid expression did not give high levels of foreign protein (Birch-Machin *et al.*, 2004; Elghabi *et al.*, 2011; De Marchis *et al.*, 2012). Thus, manipulation of the activity of stromal proteases offers the attractive possibility to stabilize unstable recombinant proteins expressed in plastids. Experiments are underway to test this idea by crossing our Clp protease RNAi lines to various transplastomic lines.

## Supplementary data

Supplementary data are available at *JXB* online.

Supplementary Protocols.

Fig. S1. Phylogenetic tree of FtsH protease sequences from Arabidopsis, tomato, and tobacco.

Fig. S2. Expression of the *clpP* gene in transplastomic start codon mutants.

Fig. S3. The Clp and FtsH proteases play important roles in plant growth and development.

Fig. S4. Morphological parameters measured in tobacco wild-type plants and the Clp and FtsH mutants.

Table S1. Clp and FtsH subunit names and protein identifiers from *Arabidopsis thaliana* and tomato (*Solanum lycopersicum*).

Table S2. Coding sequences of *CLP* and *FTSH* genes found in the genome of *Nicotiana tabacum*.

Table S3. Nucleotide sequences of the regions targeted by RNAi to trigger gene silencing for Clp and FtsH subunits.

Table S4. List of oligonucleotides used for construction of RNAi vectors and qRT-PCR experiments.

Table S5. Targeting prediction for tobacco Clp and FtsH subunits. The known location of the homologous proteins from Arabidopsis is also indicated.

## Supplementary Material

Supplementary_Figures_S1_S4_Tables_S1_S5

Supplementary_Protocols
