# Supplementary material for: Generation and characterization of a collection of knock-down lines for the chloroplast Clp protease complex in tobacco
Source: J Exp Bot. 2017 Mar 28;68(9):2199–218. doi: 10.1093/jxb/erx066 (PMC5447895; doi:10.1093/jxb/erx066)
Supplement: Supplementary_Figures_S1_S4_Tables_S1_S5 [file erx066_suppl_Supplementary_Figures_S1_S4_Tables_S1_S5.pdf]

Supplementary Figures and Tables

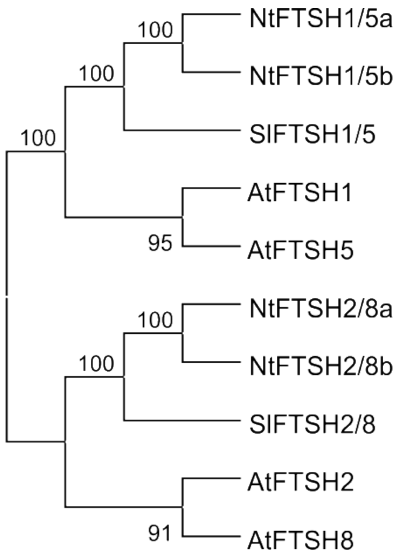

Supplementary Figure S1. Phylogenetic tree of FtsH protease sequences from *Arabidopsis* (At), tomato (Sl) and tobacco (Nt). For details of the analysis, see legend to Fig. 2.

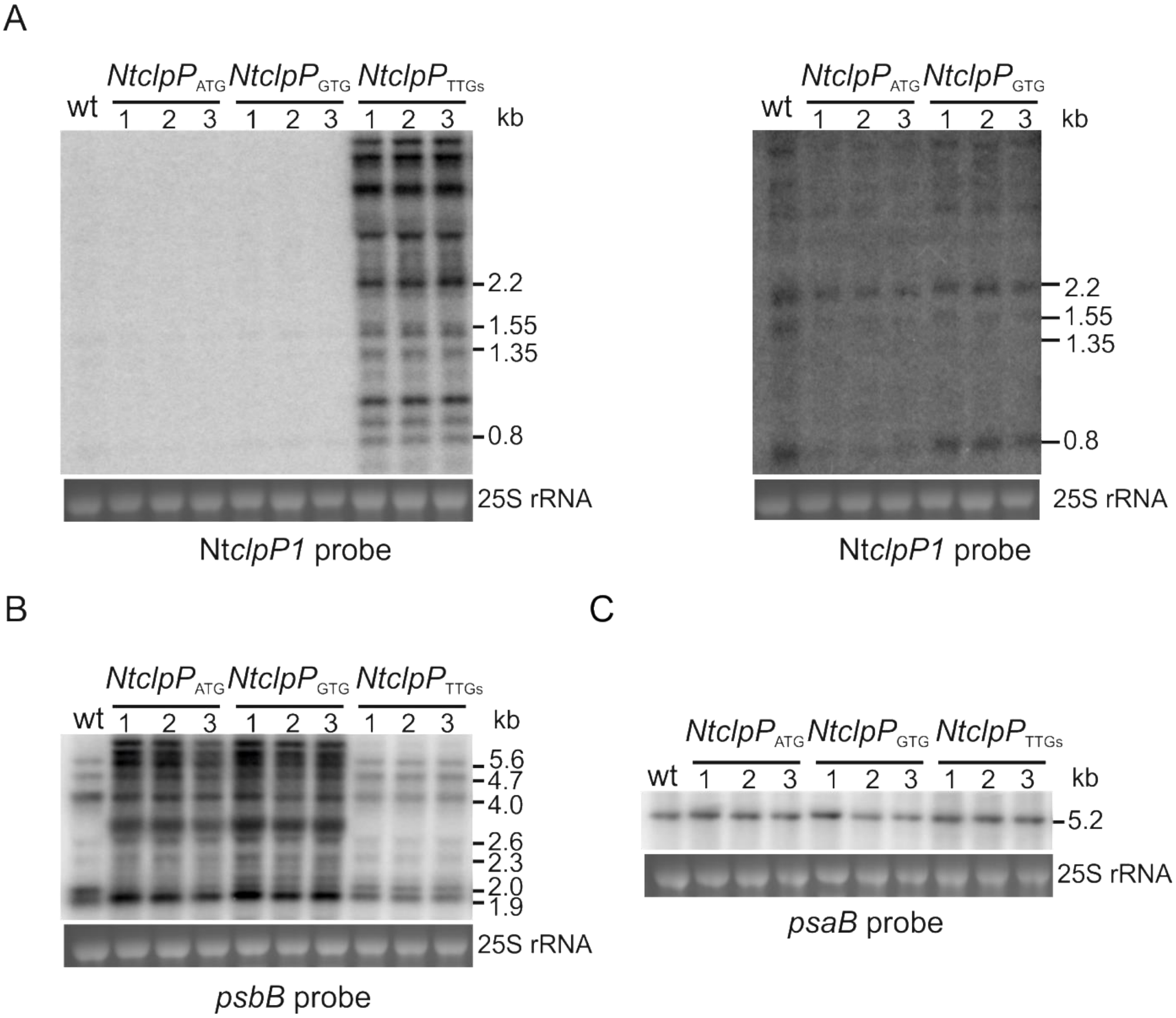

Supplementary Figure S2. Expression of the *clpP* gene in transplastomic start codon mutants. Tobacco wild type (wt), three independently generated lines of the *aadA* control (Nt*clpP*$_{\mathrm{ATG}}$) and the *clpP* start codon mutants Nt*clpP*$_{\mathrm{GTG}}$ and Nt*clpP*$_{\mathrm{TTGs}}$ were grown under standard conditions in soil until plants reached the stage of four true leaves. Total RNAs were isolated, electrophoretically separated (3 µg) in denaturing 1.5% agarose gels, blotted onto nylon membranes and hybridized with radiolabeled probes. The ethidium bromide-stained band representing the 25S rRNA is shown as a control for equal loading below each blot. (A) Accumulation of *clpP* mRNA. The *clpP*-specific probe detects the four major transcripts (2.2 kb, 1.55 kb, 1.35 kb, 0.8 kb) (Sugita and Sugiura, 1996; Kuroda and Maliga, 2002). Note that insertion of the *aadA* cassette in sense orientation (Nt*clpP*$_{\mathrm{TTGs}}$) results in enhanced accumulation of *clpP* transcripts. The right panel shows an overexposure of the wild type, the Nt*clpP*$_{\mathrm{ATG}}$ and the Nt*clpP*$_{\mathrm{GTG}}$ samples. See text for details. (B) Accumulation of *psbB* mRNA. The *psbB*-specific probe detects the previously described complex pattern of transcripts from the *psbB* operon, including polycistronic precursor RNAs and various processing intermediates (e.g., Krech *et al*., 2013) in the wild type and the Nt*clpP*$_{\mathrm{TTGs}}$ mutants (*aadA* cassette in antisense orientation to the *psbB* gene). Insertion of the *aadA* cassette in sense orientation to *psbB* (Nt*clpP*$_{\mathrm{ATG}}$ and Nt*clpP*$_{\mathrm{GTG}}$) results in a slightly enhanced accumulation of the 1.9 kb, 2.6 kb and 5.6 kb *psbB* transcripts and the accumulation of additional transcripts resulting from read-through transcription of the *aadA* cassette, as described previously (Zhou

et al., 2008; Oey *et al*., 2009; Apel and Bock, 2009). (C) Accumulation of the *psaB* mRNA served as a control. *psaB* encodes a reaction center subunit of PSI and is co-transcribed with *psaA* and *rps14* to yield a large 5.2 kb transcript.

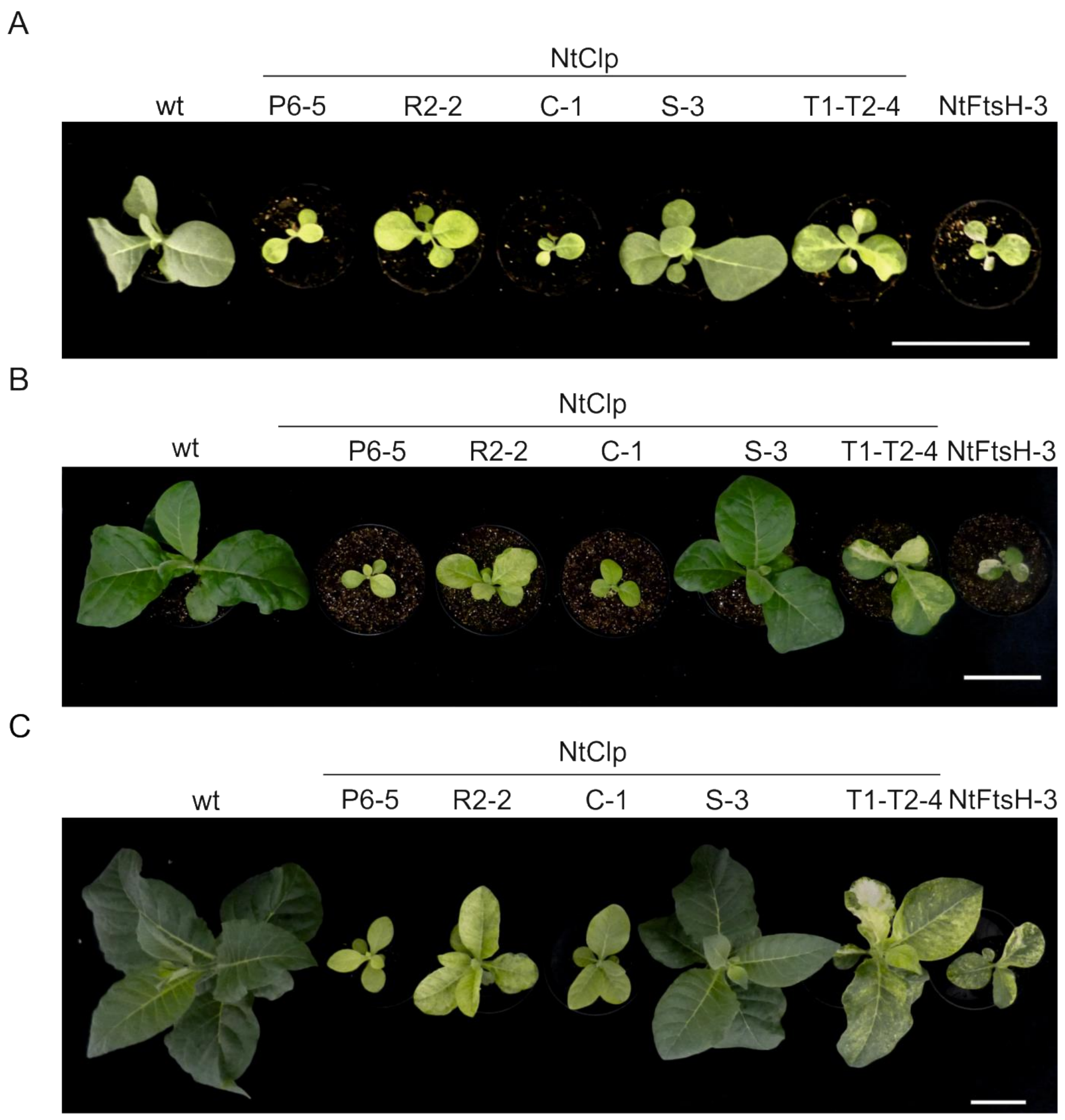

Supplementary Figure S3. The Clp and FtsH proteases play important roles in plant growth and development. (A) Clp and FtsH RNAi mutants (T_2 generation) in comparison to a wild-type plant (wt) six weeks after sowing. (B) The same plants photographed seven weeks after sowing. (C) The plants eight weeks after sowing. Scale bars: 10 cm.

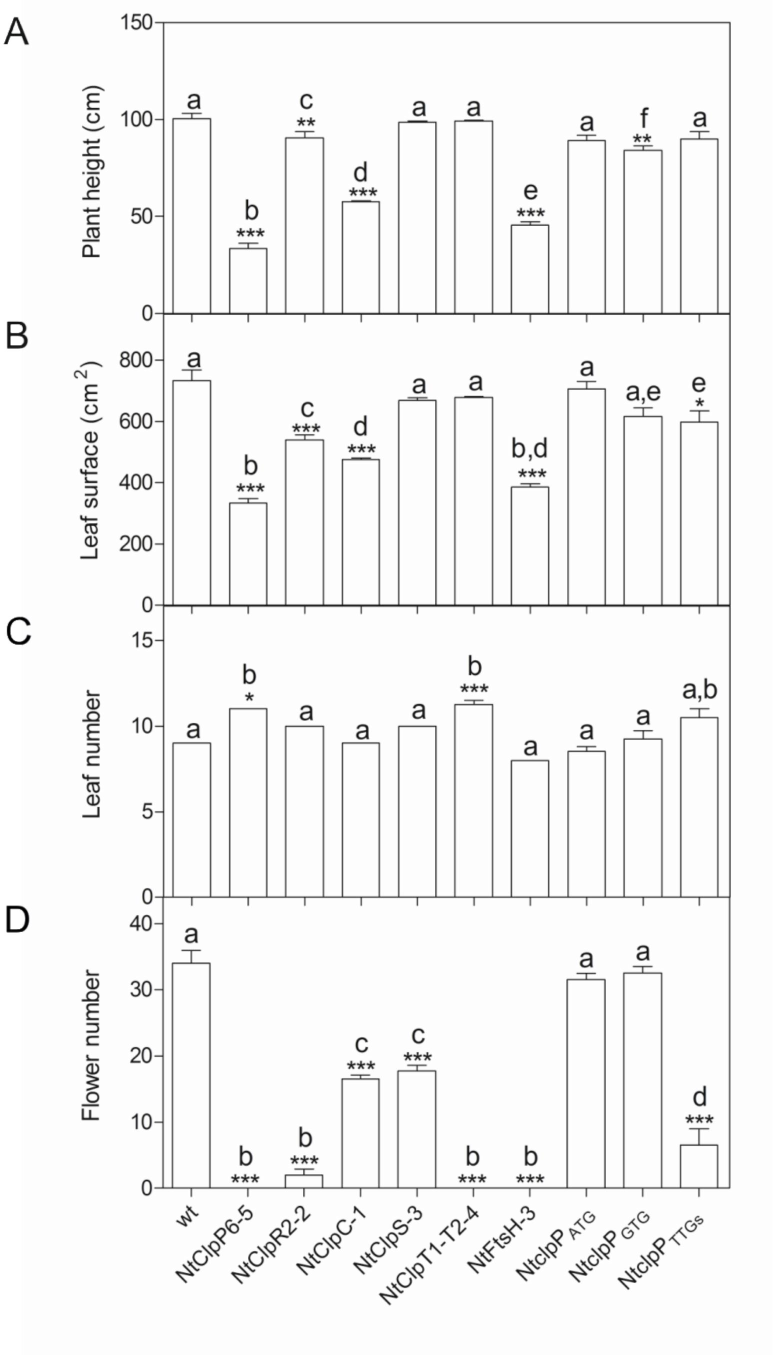

Supplementary Figure S4. Morphological parameters measured in tobacco wild-type plants (wt) and the Clp and FtsH mutants. (A) Plant height. (B) Total leaf surface. (C) Leaf number. (D) Flower number. Data were collected from 10 week-old plants raised in soil under standard greenhouse conditions. Columns and bars represent the means and SE of four biological replicates (n=4). Asterisks and letters indicate significant differences between transgenic and wild-type plants. One-way ANOVA analysis with Tuckey´s post-test (*, $P < 0.05$; **, $P < 0.005$; ***, $P < 0.0001$) was performed for all lines. Letters indicate significant differences between the lines. See also Table I.

Supplementary Table S1. Clp and FtsH subunit names and protein identifiers (ID) from *Arabidopsis thaliana* (AT) and tomato (*Solanum lycopersicum*; SL).

Protein name	Protein ID
CLPC1	AT5G50920
CLPC1B	SL03G118340
CLPC1A	SL03G118340
CLPC2	AT3G48870
CLPC-like	SL03G118360
CLPD	AT5G51070
CLPD	SL03G117950
ClpP1	ATCG00670
ClpP1	SLCG00680
CLPP3	AT1G66670
CLPP3	SL02G091280
CLPP4	AT5G45390
CLPP4	SL08G075750
CLPP5	AT1G02560
CLPP5	SL01G100520
CLPP6	AT1G11750
CLPP6	SL10G051310
CLPR1	AT1G49970
CLPR1	SL10G049710
CLPR2	AT1G12410
CLPR2	SL08G079620
CLPR3	AT1G09130
CLPR3	SL08G079620
CLPR4	AT4G17040
CLPR4	SL08G077890
CLPS	AT1G68660
CLPS	SL03G119700
CLPF	AT2G03390
CLPF	SL05G012620
CLPT1	AT4G25370
CLPT1	SL03G007110
CLPT2	AT4G12060
CLPT2	SL08G079660
FTSH1	AT1G50250
FTSH1/5	SL04G082250
FTSH2	AT2G30950
FTSH2/8	SL07G055320
FTSH5	AT5G42270
FTSH8	AT1G06430

Supplementary Table S2. Coding sequences of *CLP* and *FTSH* genes found in the genome of *Nicotiana tabacum*.

```
>NtCLPP3a (981 bp)
ATGGAGGGAGGTTGTCTAACATTCACCACAGCACCAACAAGACCTTCAACCTTATTCAAT
TACAATCCTACAATTACCAAACAATTTTACAGCAATTCACTACCAAATACTATTACACAT
GATAAACGAAGAAGAGGATTGTCAATAAAAGCATCGACCCACAACTTTTCTACAGCGAAA
CCGACGCTATCTGGGAACTGGGATGTTTCCAGTTACTCAAAAGCACCTGCTTGGTTGCCC
AGATTTGAAGAACTTGATACTACCAATATGCTTCTTCGTCAAAGGATTATCTTCTTGGGT
TCTCAGGTAGATGATATGACTGCAGATTTTGTTATAAGCCAACTATTATTTCTTGATGCC
GAAGATCAGAAAAAGGACATCAGATTGATCATTAATTCACCTGGTGGTTCAGTAACTGCT
GGGATGGGAATCTATGATGCTATGAAAATGTGCAAGGCTGATGTTTCTACAATCTGCATG
GGACTGGCTGCATCGATGGGTGCGTTTCTCCTCGCATCTGGCAGCAAGGGAAAGAGGTAC
TGCATGCCGAACGCAAGGGTGATGATTCATCAACCACTTGGAACTTCTGGTGGTAAAGCA
ACAGAGATGAGTATACGGATCAGAGAAATGGCATACCACAAGATTAAGCTTAACAAAATA
CTATCAAGGATTACTGGAAAGCCTGAAGAAAAGATTGAAGTGGATACAGATCGTGATAAT
TTCATGAATGCTTGGGAGGCTAAGGAATACGGATTGGTTGATGCTGTTATCGATGATGGC
AAACCAGGATTGGTCGCACCAACTGCAGATGCCACAGCTCCGCCAAAAACACGAGTTTGG
GATATGTGGAAGATTGAAGGAAGCAAGAAGGCAAAGAAAAATTTGCCTTCTGAAGAAAGA
ATGTTGCAAAATGGGTATGTGAGTAGTCAAGATGATGAGCAAAAGAGCAATGAACAGAAA
GATGAAGCACCTACTCTATAG
>NtCLPP3b (990 bp)
ATGGAGGGAGGTTGTCTAACATTCACTACTGCCTCGGCACCAACAAGACCTTCAACCTTA
TTCAATTACAATCCCACAGTTACCAAACAATTTTACAGAAATTCACTACAAAATACTATT
ACACATGATAAACGAAGAGGAGGATTATCAATAAAAGCATCGACCCACAATTTTTCTACA
ACAAAGCCGACACTATCTCGGAACTGGGATGTTTCGAGTTACTCAAAAGCACCTTCTTGG
TTGCCCAGATTTGAAGAACTTGATACTACCAATATGCTTCTTCGTCAAAGGATTATCTTC
TTGGGTTCTCAGGTAGATGATACGACTGCAGATTTTGTTATAAGCCAGCTATTATTTCTT
GATGCCGAAGATCAGAAAAAGGACATCAGATTGATCATTAATTCACCTGGTGGTTCAGTA
ACTGCTGGGATGGGAATCTATGATGCTATGAAATTGTGCAAGGCTGATGTTTCTACAATC
TGCATGGGACTGGCTGCATCGATGGGTGCGTTTCTCCTCGCATCTGGCAGCAAGGGAAAG
AGGTACTGCATGCCGAACGCAAGGGTGATGATTCATCAACCACTTGGAACTTCTGGTGGT
AAAGCAACGGAGATGAGTATACGGATCAGAGAAATGGCATACCACAAGATTAAGCTTAAC
AAAATACTATCAAGAATTACTGGCAAGCCTGAAGAAAAGATTGAAGTGGATACAGATCGT
GATAATTTCATGAATGCTTGGGAGGCTAAGGAATACGGATTGGTTGATGCTGTTATCGAT
GATGGCAAACCAGGATTGGTCGCACCAACTGCAGATGCCACAGCTCCGCCAAAAACACGA
GTTTGGGATATGTGGAAGATTGAAGGAAGCAAGAAGGCAAAGAAAAATTTGCCTTCTGAA
GAAAGGATGTTGCAAAATGGGTATGTGAGTAGTCAAGATGATGAGCAAAAGAGCAATGAA
CAGAAAGATGAAGCACCTACTCCTCTATAG
>NtCLPP4a (945 bp)
ATGGACTCTCTCACTCTCTCTACTTCCCTATCTCCTCACTGTTCCTCCCTCAATCTCCGA
CATGGAAGCTCTATTCCTAAGCTCCCGCCCACTTTTTGCCCTCACCCATCTCGGAAAAAC
CCCTTATCATCTCTCAAATCCTCACTTACTTCTTCTCACCAACAACCCGCTTCAAACCCC
CCCCCCTCAGATTCCCTAATTCTAAACCCGAATTTCGAAACCGGTGAGGATGATAAGTTG
TCGTTGTTACTTGCGTCGTCCCCACAAACGCCGGATACGGCCATGCGCGGCGCTGAAGCC
GACGCAATGGGGCTTTTGTTTAGGGAGAGGATTGTTTTCTTGGGTAGTCAAATTGATGAC
TTTATTGCTGATGCTATTATTAGCCAGCTTTTGTTGTTGGATGCTATGGATTCCTCTAAA
GATATTAGGCTCTTTATTAATTCCCCTGGTGGCTCCCTCAGCGCAACAATGGCTATCTTT
GACGTTGTGAAGTTGGTGAGGGCCGATGTATCCACAGTTGCACTTGGCATTGCAGCTTCC
ACAGCTTCAATAATCCTTGGTGGTGGTACCAAAGGAAAGCGCTTTGCAATGCCTAATGCT
CGAATTATGATACATCAACCACTTGGAGGGGCAAGCGGTCAAGCAATAGATGTGGAAATT
CAAGCCCGAGAAATAATGCATAATAAGGACAATGTCACCAAAATCATTTCCAATTGCACT
GGACGATCATATGAACAAGTTCAGAAAGATATTGATAGAGATCGTTACATGTCTCCTATT
GAAGCTGTAGAATATGGGCTAATTGATGGTGTTATTGATAGAGATAGCATCATTCCACTT
GAACCAGTTCCTGAAAGGGTTAAGTCTACATTGAATTATCAGGAAATCAGCAAAGATCCT
AAGAAATTTTTGAACCCAGAAATCCCTGATGATGAAATATATTAA
>NtCLPP4b (945 bp)
ATGGACTCTCTCACTCTCTCTACTTCCCTATCTCCTCACTGTTCCTCCCTCAATCTCCGA
CATGGAAGCTCTATTCCTAAGCTCTCGCCAACTTTTTGCCCTTACCCATCTCGCAAAACC
CCCTTAACATCTCTCAAGTCCTCACTTACTTCTTCTCACCAACAACCCGCTTCAAAAACC
```

CCCTTCTCAGATTCCCTAATTCTAAACCCCAATTTTGAAACCGGTGAGGATGATAAGTTG
TCGTTGTTACTTGCGTCGGCCCCACAAACGCCGGATACGGCTATGCGCGGCGCTGAAGCC
GACGCAATGGGGCTTTTGTTTAGGGAGAGGATTGTTTTCTTGGGTAGTCAAATTGATGAC
TTTATTGCTGATGCTATTATTAGCCAGCTTTTGTTGTTGGATGCTATGGATTCCTCTAAA
GATATTAGGCTCTTTATTAATTCCCCTGGTGGCTCCCTCAGCGCAACAATGGCTATCTTT
GACGTTGTGAAGTTGGTGAGGGCCGATGTATCCACAGTTGCACTTGGCATTGCAGCTTCC
ACAGCTTCAATAATCCTTGGTGGTGGTACCAAAGGAAAGCGCTTTGCAATGCCTAATGCT
CGAATTATGATACATCAACCACTTGGGGGTGCAAGCGGTCAAGCAATAGATGTGGAAATT
CAAGCCCGAGAAATAATGCATAACAAGGACAATGTCACCAAAATCATTTCCAATTGCACT
GGTCGATCATATGAACAAGTTCAGAAAGATATTGATAGAGACCGTTACATGTCTCCTATT
GAAGCTGTAGAATATGGGCTAATTGATGGTGTTATTGATAGAGATAGCATCATTCCACTT
GAACCAGTTCCTGAAAGGGTTAAGTCTACATTGAATTATCAGGAAATCAGCAAAGATCCT
AAGAAATTTTTGAACCCAGAAATCCCTGATGATGAAATATATTAA
>NtCLPP5a (894 bp)
ATGGCACATTCTTGCATAGCTACAACTTCCTCCGTCTCAAAATTCAAGTACCCAATTGAA
TCTTGCACTCTTCCCCAAGCCCACCCCGTTTCCCTCCCATTTAAACATCTTCCCTTCAGG
AAATTAAAGACTGTTGGGAAGGGGAAGAATAGGGGAAACAGCACCGTGAAAGCCGTATAT
TCCGGAGCTGAGTGGGATTCAGCTAAGGCTTCACCTACAGGAATTTGGTCTATAAGAGAT
GATTTGCAAGTTCCATCATCACCTTATTTTCCTACATATTCCCAAGCTCAAGGACCACCT
CCGATGGTACAAGAGAGATTTCAGAGTGTGATCAGCCAGCTCTTCCAATATAGGATCATA
CGATGTGGTGGAGCAGTTGATGATGATATGGCTAATATAATAGTTGCTCAGCTTCTTTAT
CTTGATGCTGTTGATCCCACAAAGGACATTGTCATGTATGTCAACTCTCCAGGAGGATCA
GTAACGGCAGGAATGGCCATATTTGATACCATGCGGCATATTCGACCTGATGTCTCCACT
GTCTGTGTTGGACTCGCTGCAAGTATGGGAGCTTTTCTTCTCAGCGCGGGCACCAAAGGA
AAGAGATATAGCTTGCCAAATTCAAGGATAATGATTCACCAGCCTCTTGGTGGTGCTCAA
GGTGGCCAAACTGATATAGATATACAGGCTAATGAGATGTTGCATCACAAAGCTAACCTG
AATGGGTACCTTGCCTACCACACTGGTCAAAGCCTTGAGAAGATTAACCAGGACACAGAT
CGTGATTTTTTCATGAGTGCAAAGGAAGCTAAAGAGTATGGGCTAATCGATGGTGTCATC
TTGAATCCCATGAAAGCCCTTCAACCTCTTGTAGCAGCTGCTGAACAATCATAG
>NtCLPP5Aa (882 bp)
ATGGCCCATTCGTGCGTGGCCACGACTTCCTCTATCTCTAAATTCAACTTCTCCTCTGAC
TCCTCTACTATTCGTGAAGCTTACCCTAATACCCTCTCCTTTAAGACCCTTCAGTTCAGG
AATTTAAAGGTGAAGAATCGGGGAAAGAGTACAGTGAAGGCCGTGTACTCTGGAGCCAAC
TGGGATTCTGGAATTACTTCACCAACTGGGATTTGGTCTATAAGAGATGACTTGCAAGTT
CCATCATCGCCCTATTTTCCTGCTTATGCCCAAGGTCAAGGACCACCTCCAATGGTGCAA
GAGCGGTTTATGAGTGTGATCAGCCAGCTCTTCCAATATAGGATCATACGATGTGGTGGG
GCAGTTGATGACGATATGGCAAATATTATAGTTGCTCAGCTTCTCTATCTTGACGCTGTT
GATCCAACAAAGGATATTGTCATGTATGTCAACTCTCCAGGAGGGTCAGTTACAGCAGGA
ATGGCCATTTTTGATACCATGCGTCATATTCGGCCTGATGTCTCCACCGTTTGTGTTGGA
CTCGCTGCGAGTATGGGGGCTTTTCTTCTTAGCGCTGGCACCAAAGGTAAGAGATATAGC
CTGCCAAACTCGAGGATAATGATTCACCAACCTCTTGGTGGTGCACAAGGTGGGCAAACT
GATATAGACATTCAGGCTAATGAGATGTTGCATCACAAAGCAAATCTGAATGGGTACCTT
GCCTACCATACTGGTCAAAGCCTTGAGAAGATCAACCAAGATACCGATCGTGATTTTTTC
ATGAGTGCAAAGGAAGCTAAAGACTATGGGCTCATTGATGGAGTCATCTTGAACCCCATG
AAAGCCCTTCAACCTCTTGTAGCAGCTGCTGAACAATCATAG
>NtCLPP5Ab (879 bp)
ATGGCCCATTCGTGCGTGGCCACGACTTCCTCTATCTCTAAATTCAACTTCTCTTCTGAT
TCCTCTACTATTCCTGAAGCTTACCCTAATACCCTCCCCTTTAAGACCCTTCAGTTCAGG
AATTTAAAGGTGAAGAATCGGGGAAAGAGTACAGTGAAGGCCGTGTACTCTGGAACCAAC
TGGGATTCTGGAATTACTTCACCAACTGGGATTTGGTCTATAAGAGATGACTTGCAAGTG
CCATCATCGCCCTATTTTCCTGCTTATGCCCAAGGTCAAGGACCACCTCCAATGGTGCAA
GAGCGGTTTATGAGTGTGATCAGCCAGCTCTTCCAATATAGGATCATACGATGTGGTGGG
GCAGTTGATGATGATATGGCAAATATTATAGTTGCGCAGCTTCTCTATCTTGACGCTGTT
GATCCAACAAAGGATATTGTCATGTATGTCAACTCTCCAGGAGGGTCAGTTACAGCAGGA
ATGGCCATTTTTGATACCATGCGGCATATTCGGCCTGATGTCTCCACCGTTTGTGTTGGA
CTCGCTGCGAGTATGGGGGCTTTTCTTCTTAGCGCTGGCACCAAAGGTAAGAGATATAGC
CTGCCAAACTCGAGGATAATGATTCACCAACCTCTTGGTGGTGCACAAGGCGGGCAAACT
GATATAGACATTCAGGCTAATGAGATGTTGCATCACAAAGCAAATCTGAATGGGTACCTT
GCCTACCATACTGGTCAAAGCCTTGAGAAGATCAACCAAGATACTGATCGTGATTTTTTC
ATGAGTGCAAAGGAAGCTAAAGAGTATGGGCTTATTGATGGAGTCATCTTGAACCCCATG
AAAGCCCTTCAACCATTAGCAGCAGCTGCTGAACAATAG
>NtCLPP5b (894 bp)

```
ATGGCACATTCTTGCATAGCCACAACTTCATCCGTCTCAAAATTCAAGTACCCGATTGAA
TCTTCCACTCTTCCCCTAGCCCACCCCGTTTCCCTCCCTCTTAAACATCTTCCCTTCAGG
AAATTAAAGACTGTTGGGAAAGTGAAGAATAGGGGAAATAGCACGGTGAAATCCGTGTAT
TCCGGAGCTGAGTGGGTTTCGGCTAAGCCTTCACCTACAGGAATTTGGTCTATAAGAGAT
GATTTGCAAGTTCCGTCTTCACCTTATTTTCCTACATATGCCCAAGGTCAAGGACCACCT
CCGATGGTACAAGAGAGATTTCAGAGTGTGATCAGCCAGCTCTTCCAATATAGGATCATA
CGATGCGGTGGAGCAGTTGATGATGATATGGCTAATATCATAGTTGCTCAGCTTCTTTAT
CTTGATGCTGTTGATCCCACAAAGGACATTGTCATGTATGTCAACTCTCCAGGAGGGTCA
GTAACGGCAGGAATGGCCATTTTCGACACCATGCGGCATATTCGACCTGATGTGTCTACT
GTCTGTGTTGGACTCGCTGCAAGTATGGGGGCTTTTCTTCTCAGCGCGGGCACCAAAGGA
AAGAGATATAGCTTGCCAAATTCAAGGATAATGATTCACCAGCCTCTTGGTGGTGCTCAA
GGTGGCCAAACTGATATAGATATACAGGCTAATGAGATGTTGCATCACAAAGCTAACCTG
AATGGGTACCTTGCCTACCACACTGGTCAAAGCCTTGAGAAGATTAACCAGGACACCGAT
CGTGATTTTTTCATGAGTGCAAAGGAAGCTAAAGAGTATGGGCTAATCGATGGTGTCATC
TTGAACCCCATGAAAGCCCTTCAACCACTTGCAGCAGCTGCTGAACAATCATAG
>NtCLPP6a (825 bp)
ATGGTAACGTCTTCCATCACCGGAACGTCAATTTTTCCAGTCTCTTTCCGGCAGCAAACG
TCTTCTTCGTCTCTGTTTTCATCTAGAAGCTTAAGGAAGCAGATAATTTCTGTTCTCCGA
AGTCCGTATTCTGATTCATCAGCTATTGGATTTTCTAACAAGACTCTGAGAACCCCGTTA
AAGCTCAATGATCACGAACCCAGTGGTCTTACCAATTCAAGCTATGGTGTTATTGAAGCA
AAAAAGGGGAATCCACCCATCATGCCTGCCGTGATAACACCAGGGGGCCCTTTGGATCTC
TCTACTGTGTTATTCAGGAATCGAATTATCTTCATTGGACAACCAATCAACTCCGCAGTT
GCTCAGAGAGTTATATCACAACTTGTGACCCTCGCAACTATCGATGAAAATGCAGATATT
TTGATCTATCTTAACTGTCCTGGTGGAAGTACCTACTCTGTCTTGGCAATATATGACTGC
ATGTCATGGATAAAGCCTAAGGTTGGTACAGTATGTTTTGGAGTAGCTGCAAGCCAAGGA
GCACTTCTTCTTGCCGGTGGAGAAAAGGGCATGAGGTACGCAATGCCAAATGCACGTATA
ATGATTCATCAACCTCAAAGTGGTTGTGGAGGTCATGTGGAGGATGTGCGGCGCCAAGTG
AATGAAGCGGTTCAATCTCGCCATAAAATCGACAAGATGTATGTTGCCTTTACTGGCCAA
TCAATTGAGAAGGTGCAACAGTACACTGAAAGGGATCGTTTTTTGTCTGTCTCTGAGGCC
ATGGAGTTTGGTCTCATAGATGGGGTGCTAGAAACAGAATACTAG
>NtCLPP6b (822 bp)
ATGGTAACGTCTGCCATCACCGGAACGTCAATTATTCCAGTCTCTTTCCGGCAGCAAACG
TCTTCTTCGTCTTTGTTTTCTTCTAGCTTAAGGAAGCAGATAGTTTCTGTTCTCCGAAGT
CCGTATTCTGATTCATCAGCTATTGGATTTTCTGGCAAGACTCTGAGAACCCCGTTAAAG
CTCAATGAGAACGAATCCAGTGGTCTTACCAATTCAAGCTATGGTGTTATCGAAGCAAAA
AAGGGGAATCCACCCATCATGCCTGCCGTGATGACACCAGGGGGACCTTTGGATCTCTCT
ACTGTGTTATTCAGGAATCGAATTATCTTCATTGGACAACCAATCAACTCCGCAGTTGCT
CAGAGAGTTATATCACAACTTGTGACCCTCGCAACTATCGATGAAAATGCAGATATTTTG
ATCTATCTTAACTGTCCTGGTGGAAGTACCTACTCTGTCTTGGCAATATATGACTGCATG
TCATGGATAAAGCCTAAGGTTGGTACAGTATGTTTCGGAGTAGCTGCAAGCCAAGGAGCA
CTTCTTCTTGCCGGTGGAGAAAAGGGCATGAGGTATGCAATGCCAAATGCACGCATAATG
ATTCATCAACCTCAAAGTGGTTGTGGAGGTCATGTGGAAGATGTGCGGCGCCAAGTGAAC
GAAGCGGTTCAATCTCGTCATAAAATCGACAAGATGTATGTCGCCTTTACTGGCCAACCA
ATTGAGAAGGTGCAACAGTACACTGAAAGGGATCGTTTTTTGTCTGTCTCAGAGGCCATG
GAGTTTGGTCTCATAGATGGGGTGCTAGAAACAGAATACTAG
>NtCLPR1a (1158 bp)
ATGGCTTCTTCTTTGCTTCTCTCTCCGCTTTCTAGCTCGACGGTTACTGAAAATCGCGAG
CTGGGTTCTGGTAAATCAACTTTCATATCCAGTCCCAATTTCTCCTTTGCAACTTCTGTT
CACAGTTGCAGGCCAAACGGCGTTCGAGGTTATTGTTACAGGTCTCCGGTAGCTAAGTCT
TTGGACCATATACCCCAAAAATTCAGACTGGAAAATCTCAAAGATGGACTACTGGACAAC
TATAAAAGTGCCCCTCAGTATCTTTACGGCCTTAGTCCTTCACAGATGGATATGTTCATG
ACAGAAGATAACCCAGTACGGCGACAGTCAGAAAGTGTCACTGAGGATAGTATATCTTCA
GCCAATAACTATCTGAGCAATGGTGGAATGTGGAGTATGTCCGGCATGAACGATCGGGGC
CCCTCGAAATACAGTATGAGTGTCAGCATGTACCGTGGAGGAGCAAGAGGATCTGGAAGA
CCTCGAACTGCGCCTCCTGATTTGCCATCTTTGCTTTTGGATGCTCGAATTGTCTATCTG
GGCATGCCTATTGTACCAGCTGTTACAGAGCTTCTTGTTGCTCAGTTTATGTGGTTGGAT
TATGACAATCCATCAAAGCCTATATACCTATATATAAACTCATCAGGCACACAGAATGAG
AAGATGGAGACTGTTGGGTCTGAAACAGAGGCATATGCCATCGCTGACACAATGGCATAC
TGCAAATCAGATATCTATACAGTGAACTGTGGCATGGCATATGGTCAAGCAGCAATGCTT
CTGTCACTGGGAAAGAAGGGGTTCCGTGCTATGCAGCCAAATTCATCTACAAAATTGTAT
TTACCTAAAGTCAGCAAATCCAGTGGAGCAGTGATAGATATGTGGATCAAGGCCAAAGAA
CTAGATGCAAACACTGAGTATTACCTTGAACTATTAGCGAAAGGAATTGGAAAACCAAAG
```

GAAGAAATCGAGAAAGATATTCAACGCCCTAAATATCTGCGGGCACAAGAAGCCATTGAC
TATGGCATTGCGGACAAGATAATCGATTCAAGAGACAATGCATTTGAGAAAAGGAACTAT
GATGAGATACTCGCCCAATCTAGAGCTATGAGGAAAGCCGGACCAGGTGCTCAGGCTGCT
CCATCTGGCTTCAGGTGA
>NtCLPR1b (1152 bp)
ATGGCTTCTTCTTTGCTTCTCTCTCCGCTTTCTAGCTCGCCGGTTACTCAAAATCGCGAG
CTGGGTTCTGGTAAATCAACTTTCATATCCAGTCCCAATTTCTCCTTTGCAATTTCTGTT
CACAGTCGCAGGCCAAACGGCGTTCGAGGTTATTGTTACAGGTCTCCGGTAGCTAAGTCT
TTGGACCATATACCCCAAAAATTCAGACAGGAAAATCTCAAAGATGGACTACAAGACAAC
TATAAAAGCGCCCCTCAGTATCTTTACGGCCTTAGTCCTTCACAGATGGATATGTTCATG
ACAGAAGATAACCCAGTCCGGCGACAGTCAGAAAGTGTCACTGAGGATAGTATATCTTCA
GCCAATAACTATCTGAGCAATGGTGGAATGTGGAGTATGTCTGGCATGAATGATCGGGGC
CCCTCGAAATACAGCATGAGCGTCAGCATGTACCGTGGAGGAGCAAGAGGATCTGGAAGA
CCTCGAACTGCGCCTCCTGATTTGCCATCTTTGCTTTTAGACGCTCGAATTGTCTATCTG
GGCATGCCTATTGTACCAGCTGTTACAGAGCTTCTTGTTGCTCAGTTTATGTGGTTGGAT
TATGACAATCCATCAAAGCCTATATACCTATATATAAACTCATCAGGCACACAGAATGAG
AAGATGGAGACTGTTGGTTCTGAAACAGAGGCATATGCCATCGCTGACACAATGGCATAC
TGCAAATCAGATATCTATACAGTGAACTGTGGCATGTCATATGGTCAAGCAGCAATGCTT
CTGTCACTGGGAAAGAAGGGGTTCCGTGCTATGCAGCCAAATTCATCTACAAAATTGTAT
TTACCTAAAGTCAGCAAATCCAGTGGAGCAGTGATAGATATGTTGGCCAAAGAACTAGAT
GCAAACACTGAGTATTACCTTGAACTATTAGCAAAAGGAATTGGAAAACCAAAGGAAGAA
ATCGAGAAAGATATTCAACGCCCTAAATATCTGCGGGCACAAGAAGCCATTGACTATGGC
ATTGCAGACAAGATAATCGATTCAAGAGACAATGCATTTGAGAAACGGAACTATGATGAG
ATACTCGCCCAATCTAGAGCTATGAGGAAATCCGGACCAGGTGCTCAGGCTGCTCCATCT
GGCTTCAGGTGA
>NtCLPR2a (870 bp)
ATGGCAGTCACTCTTCCGACCACTTCTTCCTCGTATCTAAACTCGAGAACTAGACTCCCT
CAGCCTTCTTTAAGCTGTGCCAGCAAAGTTTTTGTCGGATTAAGAGTTCAATCTCCAAAT
TCTTTTGGGATTGCAACGCCTAATGTTAATGTTGAATTTCACAATAGAGTTTACAGAAGT
ATTGAGTCCGGAACTAGAAACAGTAAACCAACACGTGCACGAGTTTCCATGATGCCCATT
GGGACACCAAGGGTGCCCTACAGAAATCCAACTGAGGGAACGTGGCAGTGGGTTGATTTG
TGGAATGCTCTTTACCGTGAACGTGTTATTTTCATCGGACAACACATAGATGAAGAATTT
AGCAACCAGATATTGGCAACAATGCTGTACCTTGACAGTATTGATGATTCCAAGAAGCTC
TACCTGTATATCAATGGCCCTGGCGGTGATCTAACTCCGAGCATGGCCATCTACGACACA
ATGCAAAGTCTGAAAAGTGCTGTTGGCACCCACTGTGTGGGCTATGCCTACAATCTTGCC
GGTTTTCTTCTTGCTGCTGGAGAAAAGGGCAATCGATTTGCGATGCCTCTTTCAAGGATT
GCACTACAATCTCCTGCTGGAGCTGCACGTGGACAGGCTGATGATATCCGTAATGAAGCA
GATGAACTTCTCAGAATTAGAGATTACCTTTTCAAGGAGTTGGCTGAGAAGACAGGCCAG
CCTGTTGAAAAGGTTCACAAGGATTTAAGTCGGATGAAGCGATTCAATGCTCAAGAAGCT
CTTGAATATGGTCTTATAGACCGTATAGTTAGGCCTCCCCGTATTAAGGCGGATGCTCCA
CGAAAGGATACCACAGCAGGTCTTGGTTAG
>NtCLPR2b (870 bp)
ATGGCAGTCACTTTTCCGACCACCTCTTCCTCGTATCTACACTCGAGAACTAAAGTCCCT
CAGCCTTCTTTAAGCTGCGCCAGCAAAGTTTTTGTCGGATTAAGAAGCCAATCTCCTAAT
TCTTATGGGATTGCAGCGTCTAATGTAAATGTTGAATTTCACAATAGAGTGTACAGAAGT
ATTGAATCCGGAACTAGAGACAGTAAACCAACACGTGCACGAGTTTCCATGATGCCCATT
GGGACACCAAGAGTACCCTACAGAAATCCAACTGAGGGAACATGGCAGTGGGTTGATTTG
TGGAATGCTCTTTACCGTGAACGTGTTATTTTCATCGGACAACACATAGATGAAGAATTT
AGCAACCAGATATTGGCAACAATGCTGTATCTTGACAGTATTGATGATTCCAAGAAGCTC
TACCTGTATATCAATGGCCCTGGTGGTGATCTAACTCCAAGCATGGCCATCTACGACACA
ATGCAAAGTCTTAAAAGTGCTGTTGGCACCCATTGTGTGGGCTATGCCTACAATCTTGCC
GGTTTTCTTCTTGCTGCTGGAGAAAAGGGCAATCGATTTGCAATGCCTCTTTCAAGGATT
GCACTACAATCTCCAGCTGGAGCTGCGCGCGGACAGGCTGATGATATTCGTAATGAAGCA
GATGAACTTCTCAGAATTAGAGATTACCTTTTCAAGGAGTTGGCTGAGAAGACAGGCCAG
CCTGTTGAAAAGGTTCACAAGGATTTAAGTCGGATGAAGCGATTCAATGCTCAAGAAGCT
CTTGAATATGGTCTTATAGACCGTATAGTTAGGCCTCCCCGTATTAAGGCAGATGCTCCA
CGAAAGGATACCACAGCAGGTCTTGGTTAG
>NtCLPR3a (975 bp)
ATGGCCACCTACTTACGGTTGCCCATGGCGTCCTCAGTTCCATGTACTTCATCATCATCG
TCTCCGCTAAAACGGCGTAGTTTTAGCGTTCCCTGTGCAGCCAATAGCAATAGCAGTACA
AAGATTCCGATGCCTCCTCTAAACCCTAAGGACCCATTTCTAAATAAGCTTGCCTCTGTT
GCTGCAAATAATCCAGAAGCTCTCTACACTCGGCCTCAAAATTCGGACATGCCGCCTTTT

```
TTGGATATTTACGACTCCCCTAAGCTCATGGCTACTCCTGCTCAGGTGGAGAGATCAGTA
TCATACAATGAGCACAGACCGAGGAGACCTCCACCAGACTTACCCTCACTGTTGCTTCAT
GGTAGAATAGTTTATATTGGCATGCCGTTGGTGCCAGCAGTCACAGAGTTAGTTGTTGCA
GAGTTGATGTACCTACAGTGGATGGATCCTAAAGAGCCAATTTACCTATACATAAACTCT
ACTGGGACTACCCGTGATGACGGTGAAACAGTTGGTATGGAAACAGAAGGTTTTGCAATT
TATGATGCCATGATGCAATTAAAAAACGAGATACACACTGTCGCAGTTGGTGCTGCCATC
GGTCAAGCGTGTCTCTTGCTTGCAGCTGGCAGTAAGGGCAAAAGGTTTATGATGCCACAT
GCCAAAGCCATGATTCAACAGCCCCGTGTGCCGTCATCTGGATTAATGCCGGCCAGCGAC
GTTTTCATCCGAGCAAAGGAGGTAATCATAAACAGAGACACCCTTGTCAAGCTTTTGGCA
AAACACACTGAAAATTCTGAAGAAACTGTTGCCAATGTGATGAGAAGACCATTTTACATG
GATGCTACCAGAGCAAGGGAATTTGGCGTCATTGATAAGATTCTTTGGCGTGGGCAAGAG
CAGATCATGGCAGATGTTTCTGCACCAGAGCAGTGGGACAAGAATGCGGGGATCAAAGTT
GCTGATGCTATTTAG
>NtCLPR3b (975 bp)
ATGGCCACCTACTTACAGTTGCCCATGGCGTCCTCAGTACCATGTACTTCAACATCATCG
TCTCCGCTAAAACGGCGTAGTTTTAGCGTTCTCTGTGCAGCCAATAGCAATAGCAGTACA
AAGATTCCGATGCCTCCGCTGAACCCTAAGGACCCATTTCTCAATAAGCTTGCATCTGTT
GCTGCAAATAATCCAGAAGCACTCTTCACTCGGCCTCAAAATTCTGATATGCCGCCTTTT
TTGGATATTTACGACTCCCCTAAGCTCATGGCTACTCCTGCTCAGGTGGAGAGATCAGTA
TCATACAATGAGCACAGACCGAGGAGACCTCCACCAGACTTACCCTCACTGTTGCTCCAT
GGTAGAATAGTTTATATTGGCATGCCGTTGGTGCCAGCAGTCACAGAGTTAGTTGTTGCA
GAGTTGATGTACCTACAGTGGATGGATCCTAAAGAGCCAATTTATCTATACATAAATTCT
ACTGGGACTACCCGTGATGATGGTGAAACAGTTGGTATGGAAACAGAAGGTTTTGCAATT
TATGATGCCATGATGCAATTAAAAAACGAGATACACACTGTCGCAGTTGGTGCTGCCATC
GGTCAGGCGTGTCTCTTGCTTGCAGCTGGTAGTAAAGGCAAAAGGTTTATGATGCCACAT
GCCAAAGCCATGATTCAACAGCCTCGCGTGCCGTCATCTGGATTAATGCCGGCCAGCGAT
GTTTTCATCCGAGCAAAGGAGGTAATCATAAACAGAGACACCCTTGTCAAGCTTTTGGCA
AAACACACTGAAAATTCTGAAGAAACTGTTGCCAATGTGATGAGAAGACCATTTTACATG
GATGCTACCAGAGCAAGAGAATTTGGCGTCATTGATAAGATTCTTTGGCGTGGGCAAGAG
CAGATCATGGCAGATGTTTCTGCACCAGAGCAGTGGGACAAGAATGCAGGGATCAAAGTT
GCTGATGCTATTTAG
>NtCLPR4a (909 bp)
ATGGAAGCTGTGACTATCGCTTCCCAATTTTCACCGGCAACGGGAGTCCGGCTATCATCA
CCGGCGAGCTGCCGTTCGTCAGCTCCCAAACGGACTCTGAGTTTTTCGCCGTCTCCGAAA
TCTTCTCTATCGACGAGCTTTATCTCCCCATTCGTCGGCGGTAGTGTACTCGCGGACTTC
TCGGGTCATAAAATTCGACCCGATTCTCTTCGCCCTTCTTCCTCTAGCTCTCGCCCCAAA
CGTGGCGTTGTCACTATGGTTATTCCTTTCTCAAGGGGAAGTGCATGGGAGCAACCTCCT
CCAGATTTAGCATCTTACTTGTATAAGAATCGAATCGTTTACTTGGGCATGTCTCTAGTT
CCATCAGTGACGGAATTGATACTAGCTGAATTTCTTTACCTTCAGTATGAGGATGAGGAA
AAGCCAATCTATCTTTATGTAAATTCTACTGGCACAACCAAGGGTGGTGAGAAGTTGGGT
TATGAGACAGAGGCTTTTGCTATATATGATGTTATGAGATATGTCAAGCCACCTATATTT
ACTCTCTGTGTTGGAAATGCATGGGGAGAAGCTGCGTTGCTTTTAGCAGCTGGTGCAAAA
GGAAATCGTGCTGCATTGCCCTCATCTACAATTATGATTAAGCAGCCAATTGCTCGGTTT
CAGGGTCAAGCAACAGATGTCGAGCTCATGAGGAAAGAAGTAAAGAATGTCAAAGCGGAA
TTGGTGAAATTGTATTCAAAGCATATTGGAAAATCACCTGAGGAGATTGAAGCGGACATA
AGACGTCCGAAGTACTTCAGTCCTAGTGAAGCAGTAGAATATGGAATTATTGATAAGGTT
CTCTACAACGAGAGGGGAAGAGAAGATAGAGGAGTTATATCTGATCTGAAGAAGGCCCAA
CTTATCTAG
>NtCLPR4b (909 bp)
ATGGAAGCTGTGACTATCGCTTCCCAATTTTCACCGGCGACGGGAGTCCGGCTATCATCA
CCGGCGAGCTGCCGTTCGTCAGCTCCCAAACGGACTCTGAGTTTTTCGCCGTCTCCGAAA
TCTTCTCTATCGACGAGCTTTATCTCCCCATTCGTCGGCGGTAGTGTACTCGCGGACTTC
TCGGGTCATAGAATTCGACCCGATTCTCTTCGACCTTCTTCCTCTAGCTCTCGCCCCAAA
CGTGGCGTTGTCACTATGGTTATTCCTTTCTCAAGGGGAAGTGCATGGGAGCAACCTCCT
CCAGATTTAGCATCTTACTTGTATAAGAATCGAATCGTTTACTTGGGCATGTCTCTAGTT
CCATCAGTGACGGAATTGATACTAGCTGAATTTCTTTACCTTCAGTATGAGGATGAGGAA
AAGCCAATCTATCTTTATGTAAATTCTACTGGCACAACCAAGGGTGGTGAGAAGTTGGGT
TATGAGACAGAGGCTTTTGCCATATATGATGTTATGAGATATGTCAAGCCACCTATATTT
ACTCTCTGTGTTGGAAATGCATGGGGAGAAGCTGCCTTGCTTTTAGCAGCTGGTGCAAAA
GGAAATCGTGCTGCATTGCCCTCATCTACAATTATGATTAAGCAGCCAATTGCTCGGTTT
CAGGGTCAAGCAACAGATGTCGAGCTCATGAGGAAAGAAGTAAAGAACGTCAAAGCGGAA
TTGGTGAAATTGTATTCAAAGCATATTGGAAAATCACCTGAGGAGATTGAAGCAGACATA
```

AGACGTCCGAAGTACTTTAGTCCTAGTGAAGCAGTAGAATATGGAATTATTGATAAGGTT
CTATACAATGAGAGGGGAAGAGAAGATAGAGGAGTTATATCCGATCTAAAGAAGGCCCAA
CTTATCTAA
>NtCLPSa (474 bp)
ATGGAGACAGCCATTTGTGGCCGAGTTGCTCTTTCTCCCCATCAAATCTTCCAACCAAAA
CCTGGAGATACAAAACCTCGTCAAAAACAATGGACAAATCGGAACACATTGATGGCAATG
CCTATAGCAGGAGTAGGCAAAGGTGGTGGGTTGTTGGAGAAGCCTGTTATCGAGAAAACA
ACTCCTGGGCGTGAATCTGAATTCGACTTGAGAAAATCAAGGAAGATGTCCCCGCCCTAT
CGTGTGATGCTGCACAATGACAACTACAACAAGAGGGAGTATGTAGTTCAAGTACTCATG
AAGGTTATACCGGGGATGACAGTCGACAATGCTGTTAATATCATGCAAGAGGCGCATTAC
AATGGTCTGGCGGTGGTGATAATCTGTGCTCAAGCTGATGCAGAAGAGCATTGCACGCAG
CTGAGAGGCAATGGTCTGCTAAGCTCCATTGAGCCTGCCAGTGGAGGTTGTTGA
>NtCLPSb (474 bp)
ATGGAGACAGCCATTTGCGGCCGAGTTGCTCTTTCTCCCCATCAAATCTTTCAACCAAAA
CCTGGAGATACGAAACCTCGTCAAAAACAATGGACAAATCGGAACACATTAATGGCAATG
CCTATAGCAGGAGTAGGCAAAGGTGGTGGGTTGTTGGAGAAGCCTGTTATAGAGAAAACA
ACTCCTGGGCGTGAATCTGAATTCGACTTGAGAAAATCAAGGAAGATTTCCCCGCCCTAT
CGTGTGATGCTGCACAATGACAACTTCAACAAGAGGGAGTATGTAGTTCAAGTACTCATG
AAGGTTATACCAGGGATGACGGTCGACAATGCTGTTAATATCATGCAAGAGGCGCATTAC
AATGGTCTTGCGGTGGTGATAATCTGTGCTCAAGTTGATGCAGAAGAGCATTGCACGCAG
CTAAGAGGCAATGGTCTGCTAAGCTCCATCGAGCCTGCCAGTGGAGGTTGTTGA
>NtCLPFa (1008 bp)
ATGGTGCAGAGTATGTCAATGAGCACTCTTGCTACTTCTAGATATTGCGGAGCTTGTGGT
TCCACCTACCTGAGGAGTCATTTTGGACAGATAAAAGAAACCGCAACTGGTTTTGGGGAA
AGATTTGTGTGGAATGATCGTCGGAAAAGCTTGTCTTTTTCTACCCACTTTGATATGCTA
AAGCCGAGAAATTTGAGAGCGCAAGCTGGATGGTTGTTTAAAGGGGGCGATCAAGCCTCA
GAAGCAAGTTGTGAGCGCAGCGAGAATGCCAATGAAGATATCTTGATGTTCTTTTTCCAG
CTGGACTTGGCTACACGTGTGCAGTATGCCTTGAACGTGGAGCAGTATGAAATTGCACAA
CAACTAAGAGAGAAGCTCACTGAGGTGGAAACAGAGGTTCTGAAGCAGCAGGAATCCCGA
AGGGGATCAGCCTCAAAGAGTGAAGCTCAAGATATGGCCATAAGCATCTTGCGTCTACGT
GCAGACCTGCAGAATGCAGTTCAGAGTGAAAACTATGATTTGGCGGCTAAATTACGAGAC
GAAATTTCCAAACTAGAGGCAGAGTCTCTAACTGCATCAATAAGAGCTCAAGCATACGTA
AATGCTCAATATGCATTTCGATTAGGCCAGAAAGTGAGGCACAAGAATTTTGGATATCGT
GGTGTAATATGTGGGATGGACCCAATATGTTGTGAATCAAGTTCATGGATGGAAACTGCT
CAAGTTGATAAGTTGAGCCGTGGTCCTGATCAGCCATTTTATCAGGTGCTGGTGGATGTA
CATACAGATCCCAATCTGTTAGTTGCATATGTTCCTGAGGAAAGTTTAGTGGCCCCCAGC
GAACCAGATAAGGATAGATTTGATCATCCCTACACCTCATTCTTATTTTTTGGGATGGAT
GCTGCTGGAGATTTCATACCAATCAAGCAGCTGCGCGAGAAATACAACAGGCCTCGACAT
GAGGTGCCATATGATCCAGAGGACGAGAAGAGTGGAGAAGGATCCTAG
>NtCLPFb (1011 bp)
ATGCTGCAAACTATGTCAATGAGTACTCTTGCTACTTCTAGATATTGCGGAGGTTGTGGA
TCCACCTATCTGAGGACGCATTTTGGACAGATAAAAGAAGCCGCAACTGGTTTTGGTGAA
AGTCATTTTGTTTGGAATGATTGTCGGAAAAGCTTGTCCTTTTATACCCACTTTGATATG
CTAAAGCCAAGAAATTTGAGAGCTCAAGCTGGATGGCTGTTTAAAGGAGGCGATCAAGGC
TCAGAAGCAGGTTGTGAGCGTAGTGAGAGTGCCAATGAAGATATCTTGATGTTCTTTTTC
CAGCTGGACCTGGCTACACGCGTGCAGTATGCTTTGAACGTGGAGCAGTATGAAATTGCA
CAACAACTAAGAGAGAAGCTTACTGAGGTGGAATCAGAGGTTTTGAAGCAGCAGGAGTCC
AGAAGGGGATCGGCCTCAAAGAGTGAAGCTCAAGATATGGCCATAAGCATCTTGCGTCTA
CGTGCAGACCTGCAAAATGCAGTTCAGAGTGAAAACTATGATTTGGCGGCTAAATTACGA
GACGAAATTTCCAAACTGGAGGCAGAATCTCTAACTGCATCAATAAGAGCTCAGGCTTAT
GTAAATGTGGAATATGCATTTCGATTAGGCCAGAAAGTGAGACACAAGAATTTTGGATAT
CGTGGTGTAATATGTGGGATGGACCCAATGTGTTGTGAATCAAGTTCATGGATGGAAACT
GCTCAAGTTGATAAGTTGAGCCGTGGTCCTGATCAGCCATTTTATCAGGTGCTGGTGGAT
GTACATACAGATCCCAATCTGTTAGTTGCATATGTTCCTGAGGAAAGTTTAGTGGCCCCC
AGCGAACCAGATAAGGATAGATTTGATCATCCCTACACCTCATTCTTATTCTTCGGGATG
GATGCTGCTGGAGATTTCATACCAATCAAGCAGCTGCGCGAGAAATACAACAGGCCTCGG
CATGAGGTGCCTTACGATCCAGAGGACGAGAAGGGTGGAGAAGGTTCCTAG
>NtCLPCAa (2772 bp)
ATGGCTAGAGCTTTAGTTCAGTCGACAAACATCCCATCGTCAGTTGCCGGTGAAAGGACG
ACAAAATTCAATGGATCTGGGAAAACGAACAGAACTGTTAAAATGCTATGCAGTGCACAA
TCTCCTTCGCTAAGGCTGCGGGATTTTTCAGGATTGCGAGGATGCAATGCAATAGATACA
CTAGTTAGATCTGAACAAACTCTCCAATCCAAGGTAGCAGCTGCAACTTCTGTGAGACGA

CCACGAGGTTGCCGATTTGTACCAAAAGCAATGTTTGAGCGCTTCACAGAGAAGGCAATA
AAAGTCATTATGCTTGCACAAGAAGAGGCCAGACGACTTGGTCACAATTTTGTTGGCACA
GAGCAGATTCTGTTAGGGCTTATTGGTGAGGGAACTGGTATTGCTGCTAAAGTTCTTAAA
TCCATGGGAATTAATTTGAAAGATGCTCGTGTGGAAGTGGAGAAGATAATTGGAAGGGGT
AGTGGATTTGTTGCCGTTGAGATCCCTTTTACGCCTCGTGCCAAGCGTGTTTTGGAACTC
TCTCTGGAGGAGGCCCGCCAGCTAGGGCATAACTATATTGGTTCGGAGCACTTGCTACTT
GGGTTGCTACGTGAAGGTGAAGGTGTGGCTGCCCGTGTCCTTGAAAATTTGGGCGCTGAC
CCCAGTAACATCCGCACTCAGGTGATCCGAATGGTTGGAGAGAGTAATGAGGCTGTTGGT
GCTAGCGTTGGAGGTGGAAGTTCTGGCCAAAAAATGCCTACACTGGAGGAGTACGGAACA
AATTTGACAAAGTTAGCTGAAGAGGGAAAATTAGATCCTGTTGTTGGAAGACAACTGCAA
ATTGAACGGGTCACTCAAATCTTGGGTCGGCGGACTAAGAACAACCCTTGTCTTATTGGA
GAACCAGGTGTTGGCAAAACAGCTATTGCTGAGGGTCTGGCACAAAGAATTGCAAACGGT
GATGTCCCTGAAACAATTGAGGGGAAGAAGGTGATAACTCTTGATATGGGTTTGCTTGTT
GCTGGGACAAAATACCGTGGAGAGTTTGAGGAAAGGCTAAAGAAGCTGATGGAGGAAATC
AAACAGAGTGATGAAATAATACTATTTATCGATGAAGTGCACACCTTGATCGGGGCTGGA
GCTGCAGAGGGGGCCATCGATGCTGCAAACATCTTGAAACCTGCCCTAGCTCGAGGTGAA
CTACAGTGTATTGGAGCTACCACACTAGATGAATACAGAAAGCATATTGAGAAAGATCCT
GCATTGGAGAGGCGATTCCAGCCAGTTAAGGTCCCTGAACCTACTGTTGATGAAACCATT
CAGATTCTGAAAGGGCTTCGTGAGAGATATGAGATTCATCACAAGCTTCGTTACACTGAC
GAGGCATTAGAGGCTGCTGCCCAGCTCTCATACCAGTACATCAGTGACCGTTTTCTGCCT
GATAAAGCAATTGACTTGATTGATGAAGCTGGTTCTCGTGTTCGACTTCGCCATGCACAG
CTCCCTGAGGAAGCAAAAGAGCTTGAGAAAGAGCTTCGTCAAATCACAAAGGAGAAGAAT
GAAGCTGTCCGCGGTCAGGATTTCGAAAAGGCTGGGGAATTACGTGATAGAGAAATGGAT
CTTAAGGCACAGATCACGGCCCTCATAGACAAAAACAAAGAGATGAGCAAGGCTGAAAGT
GAGGCTGGAGATACAGGTCCGCTTGTGACTGAAGCAGATATTCAGCACATTGTCTCTTCT
TGGACTGGCATCCCTGTTGAGAAGGTCTCGACTGACGAATCTGATCGCCTCCTAAAAATG
GAAGAAACACTTCACACTCGAATCATTGGCCAGGATGAAGCTGTCAAAGCCATTAGTCGT
GCCATTCGACGTGCGAGGGTTGGGCTCAAGAATCCCAACCGACCTATTGCTAGTTTCATC
TTTTCTGGTCCAACTGGTGTTGGGAAATCAGAACTGGCAAAGTCACTGGCTGCATATTAC
TTTGGTTCCGAAGAAGCAATGATCCGGCTTGATATGAGTGAGTTTATGGAGAGACACACA
GTCTCTAAGCTCATCGGATCACCCCCAGGTTATGTTGGTTACACAGAAGGTGGTCAATTG
ACTGAAGCCGTAAGGCGACGACCTTATACTGTTGTGCTCTTTGATGAAATTGAGAAGGCT
CATCCCGATGTCTTCAACATGATGCTTCAAATTCTTGAAGATGGAAGGCTGACAGACAGC
AAGGGGAGAACTGTCGATTTCAAGAACACACTTCTCATCATGACATCAAATGTTGGAAGT
AGTGTGATAGAGAAAGGTGGCCGTCGTATAGGTTTTGATCTCGATTATGATGAGAAGGAT
AGCAGTTACAATCGTATCAAGAGCTTAGTGACTGAAGAATTGAAACAGTACTTCAGGCCA
GAGTTTTTGAACAGATTGGATGAGATGATTGTATTCCGTCAGCTCACAAAGTTAGAGGTT
AAGGAGATAGCTGATATCATGCTCAAGGAGGTATTTGAGAGGCTGAAGGGTAAGGAGATA
GAACTTCAAGTGACAGAGAGGTTTAGAGATAGGGTGGTTGATGAAGGATATAACCCGAGC
TATGGAGCACGGCCTCTGAGAAGAGCTATCATGAGACTGTTAGAGGACAGCATGGCGGAG
AAAATGCTTGCAGGTGAGATCAAAGAAGGTGATTCAGTTATTGTGGACGTGGATTCAGAC
GGCAATGTGACTGTCCTCAATGGCAGTAGTGGTACTCCCTCAGACCCCGCTCCGGAGCCT
ATCCCTGTGTAG
>NtCLPCAb (2772 bp)
ATGGCTAGAGCTTTAGTTCAGTCGACAAACATCCCATCGTCAGTTGCTGGCGAAAGGACG
ACAAAATTCAATGGATCTGGGAAAACAAACAGAACTGTTAAAATGCTATGCAGTGCACAA
TCTCCTTCGCTAAGGCTGCGGGATTTTTCAGGATTGCGAGGATGCAATGCAATAGATACA
CTAGTTAGATCTGGACAAACTCTCCAATCCAAGGTAGCAGCTGCAACTTCTGTCAGACGA
CCACGAGGTTGCCGATTTGTACCAAAAGCAATGTTTGAGCGCTTCACAGAGAAGGCCATA
AAAGTCATTATGCTTGCACAAGAAGAGGCCAGACGACTTGGTCACAATTTTGTTGGCACA
GAGCAGATTCTGTTGGGTCTTATTGGTGAGGGAACTGGTATTGCTGCTAAGGTTCTTAAA
TCCATGGGAATTAATTTGAAAGATGCTCGTGTGGAAGTGGAAAAGATAATTGGACGGGGT
AGTGGATTTGTTGCTGTTGAGATCCCTTTTACGCCTCGTGCCAAGCGTGTTTTGGAACTC
TCTCTGGAGGAGGCCCGCCAACTAGGGCATAACTATATTGGTTCGGAACACTTGCTACTT
GGGTTGCTACGTGAAGGTGAAGGTGTGGCTGCCCGTGTCCTTGAAAATTTGGGCGCTGAC
CCCAGTAACATCCGCACTCAGGTGATCCGAATGGTTGGAGAGAGTAATGAGGCTGTTGGT
GCTAGCGTTGGAGGTGGAAGTTCTGGCCAAAAAATGCCTACACTGGAGGAGTACGGAACA
AATTTGACAAAGTTAGCTGAAGAGGGGAAATTAGATCCTGTTGTTGGAAGACAACTGCAA
ATTGAACGGGTCACTCAAATCTTGGGTCGGCGGACTAAGAACAACCCTTGTCTTATTGGA
GAACCAGGTGTTGGCAAAACAGCTATTGCTGAGGGTCTGGCACAAAGAATTGCAAACGGT
GATGTCCCTGAAACAATTGAGGGGAAGAAGGTGATAACTCTTGATATGGGTTTGCTTGTT
GCTGGGACAAAATACCGTGGAGAGTTTGAGGAAAGGCTAAAGAAGCTGATGGAGGAAATC

AAACAGAGTGATGAAATAATACTATTTATCGATGAAGTGCACACCTTGATCGGAGCTGGA
GCAGCGGAGGGGGCCATCGATGCTGCAAACATCTTGAAACCTGCCCTAGCTCGAGGTGAA
CTACAGTGTATTGGAGCTACCACACTAGATGAATACCGAAAGCATATTGAGAAAGATCCT
GCATTGGAGAGGCGATTCCAGCCAGTTAAGGTCCCTGAACCTACTGTTGATGAAACCATT
CAGATTCTGAAAGGGCTTCGTGAGAGATATGAGATTCATCACAAGCTTCGTTACACTGAC
GAGGCATTAGAGGCTGCTGCCCAGCTCTCATACCAGTACATCAGTGACCGTTTTCTGCCT
GATAAAGCAATTGACTTGATTGATGAAGCTGGTTCTCGTGTTCGACTTCGCCATGCACAG
CTCCCTGAGGAAGCAAAAGAGCTTGAGAAAGAGCTTCGTCAAATCACAAAGGAGAAGAAT
GAAGCTGTCCGCGGTCAAGATTTCGAAAAGGCTGGGGAATTACGTGATAGAGAAATGGAT
CTTAAGGCACAGATCACGGCCCTCATAGACAAAAACAAAGAGATGAGCAAGGCTGAAAGT
GAGGCTGGAGATACAGGTCCGCTTGTGACTGAAGCAGATATTCAGCACATTGTCTCTTCT
TGGACTGGCATCCCTGTTGAGAAGGTCTCGACTGACGAATCTGATCGCCTCCTAAAAATG
GAAGAAACACTTCACACTCGAATCATTGGCCAGGATGAAGCTGTCAAAGCCATTAGTCGT
GCCATTCGACGTGCGCGGGTTGGGCTCAAGAATCCCAACCGGCCTATTGCTAGTTTCATC
TTTTCTGGTCCAACTGGTGTTGGGAAGTCAGAACTGGCAAAGTCACTGGCTGCATATTAC
TTTGGTTCTGAAGAAGCAATGATCCGGCTTGATATGAGTGAGTTTATGGAGAGACACACA
GTCTCTAAGCTCATCGGATCACCCCCTGGTTATGTTGGTTACACAGAAGGTGGTCAACTG
ACTGAAGCCGTAAGGCGACGACCTTATACTGTTGTGCTCTTTGATGAAATTGAGAAGGCT
CATCCCGATGTCTTCAACATGATGCTTCAAATTCTTGAAGATGGAAGGTTGACAGACAGC
AAGGGGAGAACTGTCGATTTCAAGAACACACTTCTCATCATGACATCAAATGTTGGAAGT
AGTGTGATAGAGAAAGGTGGCCGTCGTATAGGTTTTGATCTCGATTATGATGAGAAGGAT
AGCAGTTACAATCGTATCAAGAGCTTAGTGACTGAAGAATTGAAACAGTACTTCAGGCCT
GAGTTTTTGAACAGATTGGATGAGATGATTGTATTCCGCCAGCTCACCAAGTTAGAGGTG
AAGGAGATAGCTGATATCATGCTTAAGGAGGTATTTGAGAGGTTGAAAGGTAAGGAGATA
GAACTTCAAGTGACAGAGAGGTTTAGAGATAGGGTGGTTGACGAAGGATACAACCCGAGC
TACGGAGCACGGCCTCTGAGAAGAGCTATTATGAGACTGTTAGAGGACAGCATGGCGGAG
AAAATGCTCGCAGGTGAGATCAAAGAAGGTGATTCGGTTATTGTGGACGTGGATTCAGAC
GGCAATGTGACTGTCCTCAATGGCAGTAGTGGTACTCCCTCAGACCCGGCTCCGGAGCCT
ATCCCTGTGTAG
>NtCLPCBa (2772 bp)
ATGGCTCGAGCTTTAGTTCAGTCAACTAACATCCCATCTTCAGTTGCCGGTGAAAGGACT
AGGCAATTTAGTGGATCTGGGAAAAACAAAAAAACTGTTAAAATGCTCTGTAATGTACAA
TCACCCTCCATAAGGTTGACCAATTTTACAGGACTGCGAGGGTGCAATGCAGTAGATACA
CTTGTAAAATCTGGACAAACTCTCCATTCAAAAGTGGCAGCTGCAACTTCTGTTAGGCGG
CCAAAAGGTTGCCGGTTTGTCCCAAAAGCAATGTTTGAGCGCTTCACTGAGAAAGCAATA
AAAGTCATTATGCTTGCACAAGAAGAGGCCAGACGACTAGGTCATAACTTCGTTGGCACA
GAGCAGATTTTGTTGGGTCTTATTGGTGAGGGAACTGGTATTGCAGCTAAAGTTCTTAAA
TCCATGGGAATCAATTTAAAAGATGCTCGTGTGGAGGTGGAGAAGATAATTGGAAGGGGT
AGTGGGTTTGTTGCTGTTGAGATTCCATTTACTCCTCGTGCAAAGCGTGTTCTTGAACTC
TCTCTGGAGGAAGCCCGCCAACTAGGGCATAACTACATTGGCTCGGAACACTTGCTACTT
GGATTGCTGCGTGAAGGTGAAGGTGTGGCTGCCCGTGTTCTTGAGAACTTGGGTGCTGAC
CCCAGTAACATTCGCACACAGGTCATTCGAATGGTTGGCGAGAGTAATGAGGCTGTCGGT
GCTAGTGTTGGTGGTGGAACTTCTGGACAAAAGATGCCTACATTGGAGGAGTACGGCACC
AATTTGACAAAGTTAGCTGAAGAGGGGAAACTGGATCCTGTTGTTGGAAGACAACCGCAA
ATTGAGCGCGTTACTCAAATCTTGGGTCGGCGTACAAAAAACAACCCTTGCCTTATTGGA
GAACCAGGTGTTGGCAAAACTGCTATTGCTGAAGGTTTAGCTCAAAGAATTGCTAATGGC
GATGTCCCTGAAACAATAGAGGGAAAGAAGGTCATAACACTAGATATGGGTTTGCTTGTT
GCTGGGACAAAATACCGTGGAGAGTTTGAGGAAAGACTGAAGAAACTAATGGAAGAAATT
AAACAAAGTGATGAAATAATACTCTTTATTGATGAAGTGCACACATTGATTGGAGCTGGA
GCTGCAGAGGGGGCAATTGATGCTGCAAACATCTTGAAACCTGCCCTGGCTAGAGGGGAA
CTACAGTGTATTGGAGCCACAACCCTGGATGAGTACAGAAAGCACATTGAGAAAGATCCT
GCGTTGGAGAGGAGGTTCCAGCCAGTTAAAGTCCCTGAACCTACTGTGGATGAAACCATA
CAGATCTTGAAAGGGCTTCGGGAGAGATATGAGATTCATCACAAGCTCCGTTACACCGAT
GAGGCATTAGAAGCTGCTGCCCAGCTTTCTTATCAGTACATCAGTGACCGTTTTCTGCCT
GATAAAGCAATTGATTTGATTGATGAAGCTGGTTCTCGTGTTAGACTTCGCCATGCACAG
CTCCCTGAGGAAGCAAGAGAGCTCGAGAAAGAACTTCGTCAGATTACAAAGGAGAAAAAT
GAAGCTGTGCGCGGTCAAGATTTTGAAAAGGCTGGGGAACTGCGTGATAGAGAAATGGAT
CTTAAGGCACAGATCTCAGCCCTGATAGACAAAAACAAAGAGATGAGCAAGGCTGAATCC
GAGGCTGGAGATACAGGTCCACTCGTTACAGAGGCAGATATTCAGCACATTGTGTCTTCA
TGGACTGGCATCCCTGTTGAGAAGGTTTCTACAGACGAGTCTGATCGCCTCTTGAAAATG
GAAGAAACACTTCACACCAGAATCATTGGCCAGGATGAAGCTGTTAAAGCCATTAGTCGT
GCTATCCGACGTGCTCGTGTTGGGCTCAAGAATCCCAACCGACCTATTGCCAGTTTCATC

TTTTCTGGTCCAACTGGTGTTGGGAAATCAGAACTGGCCAAGGCTTTAGCAGCATACTAC
TTTGGTTCTGAAGAAGCAATGATCCGGCTTGATATGAGTGAGTTTATGGAGAGACACACC
GTCTCTAAACTCATTGGTTCACCCCCTGGTTATGTTGGTTATACTGAAGGTGGTCAACTG
ACTGAAGCTGTGAGGCGTCGACCTTACACTGTTGTGCTCTTTGATGAGATTGAGAAGGCT
CATCCTGATGTCTTCAACATGATGCTTCAAATTCTTGAAGATGGAAGATTGACAGACAGC
AAGGGCAGAACTGTCGACTTCAAGAATACACTTCTCATCATGACATCGAATGTCGGAAGC
AGTGTGATAGAGAAAGGAGGCCGTCGTATAGGTTTTGATCTAGATTATGACGAGAAGGAT
AGCAGTTACAACCGTATCAAGAGCTTGGTGACTGAGGAGTTGAAACAGTACTTCAGGCCA
GAGTTCTTGAACAGATTGGATGAGATGATTGTATTCCGTCAGCTAACTAAGTTAGAGGTG
AAGGAGATAGCTGATATCATGCTTAAGGAGGTCTTTGAGAGGTTGAAAAATAAGGAGATA
GAACTTCAAGTGACAGAGAGGTTTAGAGACAGGGTGGTTGATGAAGGGTACAACCCAAGC
TACGGAGCAAGACCGTTGAGGAGAGCTATTATGAGACTGCTGGAAGACAGCATGGCTGAG
AAGATGCTTGCAGGTGAGATCAAAGAAGGTGATTCAGTAATTGTGGACGTGGACTCTGAT
GGCAATGTGACCGTCCTCAATGGCACTAGCGGAACTCCCTCAGATCCAGCTCCTGAGCCT
ATCCCTGTGTAG
>NtCLPCBb (2772 bp)
ATGGCTAGAGCTTTAGTTCAGTCAACCAACATCCCATCTTCAGTTGCCGGTGAAAGGACT
AGGCAATTTAGTGGATCTGGGAAAAACAAAAAAACTGTTAAAATGCTATGTAATGTACAA
TCACCCTCCATAAGGTTGACCAATTTTACAGGACTGCGAGGGTGCAATGCAATAGATACA
CTTGTAAAATCTGGACAAACTCTCCATTCAAAAGTGGCAGCTGCAACTTCTGTCAGACGG
CCAAGAGGTTGCCGGTTTGTCCCAAAAGCAATGTTTGAGCGCTTCACTGAGAAAGCAATA
AAAGTCATTATGCTTGCACAAGAAGAGGCGAGACGACTAGGTCATAACTTCGTTGGCACA
GAGCAGATTTTGTTGGGTCTTATTGGTGAGGGAACTGGTATTGCAGCTAAAGTTCTTAAA
TCCATGGGAATCAATTTAAAAGATGCCCGTGTGGAGGTGGAGAAGATAATTGGAAGGGGT
AGTGGGTTTGTTGCTGTTGAGATTCCATTTACTCCTCGTGCAAAGCGTGTTCTTGAACTC
TCTCTGGAGGAAGCCCGCCAACTAGGGCATAACTACATTGGCTCGGAACACTTGCTGCTT
GGACTGCTGCGTGAAGGTGAAGGTGTGGCTGCCCGTGTTCTTGAGAACTTGGGTGCTGAC
CCCAGTAACATTCGCACACAGGTCATTCGAATGGTTGGCGAGAGTAATGAGGCTGTTGGT
GCTAGTGTTGGTGGTGGAACTTCTGGACAAAAGATGCCTACATTGGAGGAGTACGGCACC
AATTTGACAAAGTTGGCTGAAGAGGGGAAACTGGATCCTGTTGTTGGAAGACAGCCGCAA
ATTGAGCGCGTTACTCAAATCTTGGGTCGGCGTACAAAAAACAACCCTTGCCTTATTGGA
GAACCAGGTGTTGGCAAAACTGCTATTGCTGAAGGTTTAGCTCAAAGAATTGCTAATGGC
GATGTCCCCGAAACAATAGAGGGAAAGAAGGTCATAACACTAGATATGGGTTTGCTTGTT
GCTGGGACAAAATACCGTGGAGAGTTTGAGGAAAGACTGAAGAAACTAATGGAAGAAATT
AAACAAAGTGATGAAATAATACTCTTTATTGATGAAGTGCACACATTGATTGGAGCTGGA
GCTGCAGAGGGGGCAATTGATGCTGCAAACATCTTGAAACCTGCCCTAGCTAGAGGGGAA
CTACAGTGTATTGGAGCCACAACCCTGGATGAGTACAGAAAGCACATTGAGAAAGATCCT
GCATTGGAGAGGAGATTCCAACCAGTTAAAGTCCCTGAACCTACTGTGGATGAAACCATA
CAGATCTTGAAAGGGCTTCGGGAGAGATATGAGATTCATCACAAGCTCCGTTACACCGAT
GAGGCATTAGAAGCTGCTGCCCAGCTTTCTTATCAGTACATCAGTGACCGTTTTCTGCCT
GATAAGGCAATTGATTTGATTGATGAAGCTGGTTCCCGTGTTAGACTACGCCATGCACAG
CTCCCTGAGGAAGCAAGAGAGCTCGAGAAAGAACTCCGTCAGATTACTAAGGAGAAAAAT
GAAGCTGTGCGAGGTCAAGATTTTGAAAAGGCGGGGGAGCTGCGTGATAGAGAAATGGAT
CTTAAGGCACAGATCTCAGCCCTGATAGACAAAAACAAAGAGATGAGCAAGGCTGAATCT
GAGGCTGGAGATACAGGTCCGCTCGTTACAGAGGCAGATATTCAGCACATTGTGTCTTCA
TGGACTGGCATCCCTGTCGAGAAGGTTTCAACAGATGAATCTGATCGCCTCTTAAAAATG
GAAGAAACACTTCACACCAGAATCATTGGCCAGGATGAAGCTGTGAAAGCCATTAGTCGC
GCTATCCGACGTGCTCGTGTTGGGCTCAAGAATCCCAACCGACCTATTGCCAGTTTCATC
TTTTCTGGTCCAACTGGTGTTGGGAAATCAGAACTGGCCAAGGCTTTAGCAGCGTACTAC
TTTGGTTCTGAAGAAGCAATGATCCGGCTTGATATGAGTGAGTTTATGGAGAGACACACT
GTCTCTAAACTCATTGGTTCACCCCCTGGTTATGTTGGTTACACTGAAGGTGGTCAACTG
ACTGAAGCTGTGAGGCGTCGACCTTACACTGTTGTGCTCTTTGATGAGATTGAGAAGGCT
CATCCTGATGTCTTCAACATGATGCTTCAAATTCTTGAAGATGGAAGATTGACAGACAGC
AAGGGCAGAACTGTCGACTTCAAGAATACACTTCTCATCATGACATCGAATGTCGGAAGC
AGTGTGATAGAGAAAGGAGGCCGTCGTATAGGTTTTGATCTAGATTATGACGAGAAGGAT
AGCAGTTACAACCGTATCAAGAGCTTGGTGACTGAGGAGTTGAAACAGTACTTTAGGCCA
GAGTTCTTGAACAGATTGGATGAGATGATTGTATTCCGTCAGCTCACTAAGTTAGAGGTG
AAGGAGATAGCTGATATCATGCTTAAGGAGGTCTTTGAGAGGTTGAAAAATAAGGAGATA
GAACTTCAAGTGACGGAGAGGTTTAGAGACAGGGTGGTTGACGAAGGGTACAACCCAAGC
TACGGAGCAAGACCGTTGAGGAGAGCTATTATGAGACTGCTGGAAGACAGCATGGCCGAG
AAGATGCTTGCAGGTGAGATCAAAGAAGGTGATTCAGTAATTGTGGACGTGGACTCTGAT
GGTAATGTGACCGTCCTCAATGGCACTAGCGGAACTCCCTCAGATCCAGCTCCTGAGCCT

ATCCCTGTGTAG
>NtCLPDa (2907 bp)
ATGGAATTAACGTGTTCTTCTCCACTTTCTGTGAACTCAACAATCAGCTTCAATCCTCAG
CTTCGTCGCTATGGCTCTGTGTACCCTCACAAGAGATGTCAAACCGTGTTTTCGCTTTTC
CCATATTGCCCTTCTTCCTCCTCTCATATTACTATTACTACCGCCACTACCGCTGCTTGT
AGTACTAGCAGTTCTACTTCGTCCCTTTTTGGAATTTCCCTTTCTCATAGGCCCTGCAGC
TCAATTCCTCGTAAAATCAAGCGCTCTTTGTATATTGTCTCTGGAGTGTTCGAGAGATTT
ACTGAGAGATCAATCAAAGCTGTGATGTTTTCTCAAAAAGAAGCAAAGGCTTTGGGCAAA
GATATGGTGTATACACAGCATCTTTTGCTGGGTCTGATCGCAGAGGATCGTAGCCCTGGT
GGATTCCTTGGTTCTCGAATAACAATTGATAAAGCCCGCGAAGCTGTTCGGAGCATATGG
CATGATGACGTGGAGGATGATAAAGAAAAATTGGCTTCTCAGGACTCCGGTTCTGCTACG
TCGGCTACTGATGTGGCGTTTTCTTCAAGTACGAAGCGCGTTTTTGAGGCTGCGGTTGAG
TATTCAAGGACCATGGGGCATAATTTTATTGCTCCCGAGCATATGGCCTTTGGTTTGTTT
ACTGTTGATGATGGTAACGCCACTCGTGTGCTCAAGAGGTTAGGAGTAAATGTAAATCGT
TTGGCAGCTGAGGCAGTTTCCAGGCTTCAAGGAGAGCTTGCTAAAGATGGTAGAGAGCCA
ATTTCATTCAAAAGGTCGCGTGAGAAATCCTTTCCTGGAAAAATAACTATCGACAGATCC
GCTGAGAAAGCAAAAGAGAAAAATGCGCTGGAGCAATTCTGTGTAGATCTTACTGCCCGT
GTGAGTGAGGGCCTTATAGACCCAGTAATTGGCAGAGAGATTGAAGTTCAGCGAATTATC
GAGATTCTCTGCCGTCGAACCAAAAACAATCCTATTCTGCTTGGTCAAGCTGGGGTTGGG
AAAACAGCGATAGCCGAAGGGCTGGCGATAAACATTGCTGAGGGAAATATTCCTGCATTT
TTAATGAAAAAGCGGGTAATGTCTTTAGACATTGGCCTACTCATTTCAGGTGCAAAGGAG
AGGGGCGAACTAGAGGGGCGTGTGACTACATTAATTAAGGAGGTCAAAAAGTCAGGCAAT
ATCATTCTATTCATAGATGAGGTCCACATCCTTGTTGGTGCTGGTACAGTTGGAAGGGGA
AATAAGGGTTCTGGTCTTGACATTGCTAATTTGCTAAAGCCAGCACTTGGGCGGGGCGAA
CTGCAGTGTATTGCATCTACCACCATGGATGAGTTCAGATTGCATATTGAGAAGGACAAG
GCCTTTGCCCGAAGATTCCAGCCTGTCTTGATTAATGAACCAAGTCAGGCGGATGCTGTC
CAGATACTGTTGGGATTGCGTGAGAAATATGAGTCACATCATAAGTGTATATACAGTTTG
GAAGCCATAAATGCTGCTGTGCAACTGTCAGCAAGATATATACCGGATAGGTATCTTCCT
GACAAAGCTATTGATCTTATTGATGAGGCTGGTAGTAAATCTCGTATGCAAGCTCACAAA
AGAAGAAAGGAACAGCAGATATCTGTACTCTCACAATCACCAAGTGATTATTGGCAGGAG
ATTAGAGCTGTTCAAGCCATGCATGAAGTGATCTTGGCAAGCAAGCTGACAGAAAATGAT
GATGCATCTCGTTTGAATGATGGCAGTGAACTTCATTTACAGCCAGCATCACCTTCTACA
TCTGATGAAGATGAACCTCCGGTAGTTGGACCTGAGGAAATAGCAGCAGTTGCTTCACTC
TGGACAGGCATTCCCCTTAAGCAGCTTACTGTTGATGAAAGAATGCTTCTGGTTGGTCTT
GATGAGCAGCTTAAAAAAAGGGTTGTTGGTCAGGATGAGGCTGTTGCAGCCATTTGTCGG
GCTGTTAAGAGATCTAGAACTGGTCTTAAGGACCCAAATAGACCAATTTCGGCAATGCTC
TTCTGTGGTCCTACTGGAGTTGGAAAATCTGAACTAGCTAAAGCTTTGGCAGCGTCTTAT
TTTGGTTCTGAATCTGCCATGCTAAGATTGGATATGAGTGAATACATGGAGCGGCATACT
GTGAGCAAGTTAATTGGATCGCCTCCTGGTTATGTAGGCTATGGAGAAGGAGGAACACTT
ACTGAAGCTATCAGAAGAAAGCCCTTCACTGTAGTGCTGCTAGATGAGATTGAAAAGGCT
CATCCTGACATATTCAATATTCTCCTTCAGTTGTTTGAAGATGGTCACCTAACAGACTCT
CAGGGAAGAAGAGTGTCATTTAAGAATGCCCTGATAGTGATGACTTCTAATGTGGGTTCT
ACAGCCATAGTAAAGGGTAGACAGAATACTATTGGCTTCTTGCTTGCTGATGATGAATCA
GCAGCCTCCTATGCTGGTATGAAAGCAATAGTGATGGAAGAGCTCAAGACATATTTCCGC
CCTGAGTTGATGAATAGGCTAGACGAAGTAGTAGTATTCCGTCCTCTAGAGAAGCCCCAG
ATGCTCCAGATACTAGACCTGATGCTGCAGGAGGTGAGGGCTAGGCTTGTTTCATTGGAA
ATAAGCTTGGAGGTGTCAGAAGCAGTAATGGAGCTTATATGCCAACAAGGATTTGACAGA
AACTACGGTGCACGCCCTCTTAGGAGGGCTGTTACTCAAATGGTTGAAGATCTCCTGAGT
GAATCTTTCCTTTCTGGGGATCTCAAGCCTGGTGATGTTGCTATAATTAATTTAGATGAG
TCTGGAAATCCTGTTGTCGCTAACAAGTCAACCCAGAGTATCCATTTGTCTGATGCGAAC
GGAAATCCAGTTGTCACAAACCGGTGA
>NtCLPDb (2907 bp)
ATGGAATTAACGTGCTCGTCTCCACTTTCTGTGAACTCAACAATCAGCTTCAATTCTCAG
CTTCGTCGCTATGTTTCTGCTTACCCTCACAAGAGATGTCAAACCGTGTTTTCGCTTTTC
CCATATTGCCCTTCTTCCTCCTCTCATATTACTACTACTACTACTACTACCGCCACCACC
GCTACTAGCAGTTCTACTTCGTCCCTTTTTGGAATTTCCCTTTCTCATCGACCCTGTAAT
TCAATTCCTCGTAAAATCAAGCGCTCTTTGTATATTGTCTCTGGAGTGTTCGAGAGATTT
ACTGAGAGATCGATCAAAGCTGTGATGTTTTCACAGAAAGAAGCAAAGGCTTTGGGCAAA
GATATGGTGTATACACAGCATCTTTTGCTGGGTCTGATCGCAGAGGATCGTAGCCCTGGT
GGATTCCTTGGTTCTCGGATAACTATTGATAAAGCCCGCGAAGCTGTTCGGAGCATATGG
CATGATGACGTGGAGGATGATAAAGCGAAACTGGCTTCTCAGGACTCCGGTTCTGCTACG
TCGGCTACTGATGTAGCGTTTTCTTCAAGTACAAAGCGCGTATTTGAGGCGGCGGTTGAG

TATTCAAGGACCATGGGACATAATTTTATTGCTCCTGAGCATATTGCTTTTGGTTTGTTC
ACCGTTGATGATGGTAACGCTACTCGTGTGCTCAAGAGGTTAGGAGTAAATGTAAATCGG
TTGGCAGCTGAGGCAGTTTCCAGGCTTCAAGGAGAGCTTGCTAAAGATGGTAGAGAGCCA
ATTTCATTCAAAAGGTCGCGCGATAAATCTTTTCCTGGAAAAATAACTATCGACAGATCC
GCTGAGAAAGCAAATGAGAAAAATGCTCTGGAGCAATTTTGTGTGGATCTTACTGCCCGT
GCAAGTGAGGGCCTTATAGACCCAGTAATTGGCAGAGAGACTGAAGTTCAGAGAATTATC
GAGATTCTCTGCCGTCGAACCAAAAACAATCCTATTCTGCTTGGTCAAGCTGGGGTTGGG
AAGACAGCAATAGCCGAAGGGCTGGCGATAAACATTGCTGAGGGAAATATCCCTGCATTT
TTAATGAAAAAGCGGGTAATGTCTTTAGACATTGGACTACTGATTTCAGGTGCAAAGGAG
AGGGGTGAACTAGAGGGGCGCGTGACTACATTAATTAAGGAGGTAAAAAAGTCAGGCAAT
ATCATTCTATTCATAGATGAGGTCCACACCCTTGTTGGTGCTGGCACAGTTGGACGGGGA
AATAAGGGTTCTGGTCTTGACATTGCTAATTTGCTAAAGCCAGCACTTGGGCGGGGTGAA
CTGCAGTGTATTGCATCTACCACCATGGATGAGTTCAGATTGCACATTGAGAAGGACAAG
GCCTTTGCCCGAAGGTTCCAGCCCGTCTTGATTAATGAACCAAGTCAGGCGGATGCTGTC
CAGATACTGTTGGGATTGTGTGAGAAATATGAGTCACATCATAAGTGTAGATACAGTTTG
GATGCCATAAATGCTGCTGTGCAACTGTCAGCAAGATATATACCGGATAGGTATCTTCCC
GACAAAGCTATTGATCTTATTGATGAGGCTGGTAGTAAATCTCGTATGCAAGCTCACAAA
AGAAGAAAGGAACAGCAGGTATCTGTACTCTCACAATCACCTAGTGATTATTGGCAGGAG
ATTAGAGCTGTCCAAGCCATGCATGAAGTGATCCTGGCAAGCAAGCTGACAGAAAATGAT
GATGCATCTTGTTTGAATGATGACAGTGAACTTCATTTACAGCCAGCATCGTCTTCTACA
TCTGATGAAGATGAACCTCCGGTAGTTGGACCTGAGGAAATAGCAGCAGTTGCTTCACTC
TGGACAGGCATTCCCCTTAAGCAGCTTACTGTTGATGAAAGAATGCTTCTGGTTGGTCTT
GATGAGCAGCTTAGAAAAAGGGTTGTTGGTCAGGATGAGGCTGTTACAGCCATTTGTCGG
GCTGTTAAGAGATCCAGAACTGGCCTTAAGGACCCAAATAGACCAATTTCGGCAATGCTC
TTCTGTGGTCCTACTGGAGTTGGAAAATCTGAACTAGCTAAAGCTTTGGCAGCGTCTTAT
TTTGGTTCTGAATCTGCCATGCTAAGATTGGATATGAGTGAATACATGGAGCGGCATACT
GTGAGCAAGTTAATTGGATCGCCTCCTGGTTATGTAGGCTATGGAGAAGGAGGAACACTT
ACTGAAGCTATCAGAAGAAAGCCTTTCACTGTAGTGCTGCTAGATGAGATTGAAAAGGCT
CATCCTGACATATTCAATATTCTCCTTCAGTTGTTTGAAGATGGTCACCTAACAGACTCT
CAGGGAAGAAGAGTATCATTTAAGAATGCCCTGATAGTGATGACTTCTAATGTGGGTTCT
ACAGCCATAGTAAAGGGTAGACAGAATACTATTGGCTTCTTGCTTGCTGATGACGAATCA
GCAGCCTCCTATGCTGGTATGAAAGCAATAGTGATGGAAGAGCTAAAGACATATTTCCGC
CCAGAGTTGATGAATAGGCTAGACGAAGTAGTAGTATTCCGTCCTCTAGAGAAGCCCCAG
ATGCTCCAGATACTAGACCTGATGCTGCAGGAGGTAAGGGCTAGGCTTGTTTCATTGGAA
ATAAGCTTGGAGGTGTCAGAAGCAGTAATGGAGCTTATATGCCAACAAGGATTTGACAGA
AACTACGGTGCACGCCCTCTTAGGAGGGCTGTTACTCAAATGGTTGAAGATCTCCTGAGT
GAATCTTTACTTTCTGGGGATCTCAAGCCTGGTGATGTAGCTATAATTAATTTAGATGAG
TCTGGAAATCCTGTTGTCGCTAACAAGTCAACCCAGAGTATCCATTTGTCTGATGCGAAC
GGAAATCCAGTTGTCACAAACAGGTGA
>NtCLPT1a (708 bp)
ATGGCGACTCACAGTTTTTCATTACTTTCAATCCCGTCTTCAACTTCGACTTCTTACAAT
AGACAAAATGATAATAATACTCTAACTCAAAAGTATTGTAAGATTCTAGCTTCTTCTTTT
ACTGGCGGAATGGTCTCTATTCGGCCGCAGAATTTGAACGTTTTTGCTCTCAAACGGCGT
CGTTCGACCGTGGCAACGGTCTCCTTCAGCCTCCCAACTGCAAAACCAGAGAGAGCTCCT
TCTGATATACAGCCCAGATGGTCGGCAAGAGCAATAAAGGCATTTGCAATGGCTGAATTA
GAAGCAAGGAAGCTCAAGTACCCAAATACTGGCACCGAAGCTCTTTTAATGGGAATCTTG
GTCGAAGGAACCAGTTTGGCTGCCAAGTTTTTGAGAGCAAACGGTGTAACCCTTCTCAAG
GCGAGGGAAGAAACTGTGAAGTTGCTTGGAAAATCTGATATGTATTTTTTCAGTCCAGAG
CATCCTCCCCTTACTGAACCAGCTCAAAGGGCTCTTGACTGGGCAGTTGATGAGAAATTG
AAATCAGGTGAAAGTGGGGAGATAACCATAGCATATCTGGCTCTTGGTATTTGGTCAGAA
AAGGAATCAGCTGGGCATAAAATAATGGCTACACTTGGTTTTGATGACGAGAAGGCTAAA
GAGCTAGCCAGATCTATGGACAAGGACATTGAGATGAGCTATAAATAA
>NtCLPT1b (708 bp)
ATGGCGACTCACAGTTTTTCATTACTTTCAATCCCGTCTTCAACTTCGACTTCTTACAAT
AGACAAAGTGATAATAATACTTTAACCCAGAAGTATTGCAAGATTCTAGCTACTTCTTTT
ACTGGCGGAATGCTCTCTATTCGACCGCAGAATTTGAACGTTTTTACTCTCAAACGGCGT
CGTTCGACCGTAGCAACGGTCTCCTTCAGCCTCCCAACTGCAAAACCAGAGAGAGCTCCT
TCTGAGATACAGCCCAAATGGTCGGCAAGAGCAATAAAGGCATTTGCAATGGCTGAATTA
GAAGCAAGGAAGCTCAAGTACCCAAATACTGGCACTGAAGCTCTTTTAATGGGAATCTTG
GTTGAAGGAACCAGTTTGGCTGCCAAGTTTCTGAGAGCAAACGGTGTAACCCTTCTCAAG
GCGAGGGATGAAACTGTAAAGTTGCTTGGAAAATCCGATATGTATTTTTTCAGTCCAGAG
CATCCTCCCCTTACTGAACCAGCTCAAAGGGCTCTTGACTGGGCAGTCGATGAGAAATTG

AAATCAGGTGAAAGTGGGGAGATAACCATAGCACATCTGGCTCTTGGTATTTGGTCAGAA
AAGGAATCAGCTGGGCATAAAATAATGGCTACACTTGGTTTTGATGACGAGAAGGCTAAA
GAGCTAGCCAGATCTATGGACAAGGACATTGAGATGAGCTATAAATAA
>NtCLPT2a (708 bp)
ATGGCTGCTCTCTCCACAGTGGCAGCCACCTCAAAGGGCGTCAACCAGCTCAATTATGGA
GTAGGACCCAATTCGTCGTTCTGGACAACAAACCCACAAATTTTGCATAGTCAATGGCTT
GGTACTCCAATCAAATTCTCTCTTCAGTCCAAGACTTTCAAACCATTTCTTACAAAACAT
TGCCCCATTAAAGCCGCCATCTCCTTTAGTCTTCCTTATGGAAAGTCGGAGGCTGCAGTA
TCTATAGAGAAAATTCCTAAATGGTCGTCAAAGGCGATAAAATCATTTGCCATGGGTGAA
TTGGAAGCAAGAAAACTTAAATATCCCACTACTGGAACAGAAGCTCTTCTCATGGGAATT
TTGATTGAAGGGACAAATTTTGCTTCAAAATATTTGAGGGCGAACAGCATTACTCTCTTT
AAAGTACGTGAAGAAACTGTCAAAATACTTGGAAAAGCTGACATGTGGTTTTTTAGTCCT
GAGCATCCTCCTTTGACTGAAGATGCACAAAAGGCTCTTGATTGGGCGGTCGATGAAAAA
CTCAAATCCAGTGACAATGGGGAGATAACAACTACTCATTTGCTCCTTGGGGTGTGGTCA
CAAGTAGGATCACCAGGTTATAAGATATTGTCTGCTTTGGGCTTCAATGATGAAAAAGCT
CAAGAGTTAAAGAAGGTAATTTCAGAACCTGGATTTGTGGATGATTAA
>NtCLPT2b (708 bp)
ATGGCTGCTCTCTCCACAGTGGCAGCCACTTCAAATGGCATCAACCAGCTCAATTACAGA
GTAGGGCCCAATTCATCTGTCTGTACAACAAACCCACAAAATTTGCATAGTCAATGGCTT
GGTACTCAAATCAAAATCTCTCTTCAGTCCAACAATTTCAAACCATTTCTTACAAAACAT
TGCCCCATTAAAGCCACCATCTCCTTTAGCCTTCCTTATGGAAAGTCAGAGGCTGCAGTA
TCTATAGAGAAAATTCCCAAATGGTCGTCAAAGGCGATAAAATCGTTTGCCATGGGTGAA
CTGGAAGCAAGAAAACTTAAATATCCCACTACTGGAACGGAAGCTCTTCTCATGGGGATT
TTGATTGAAGGGACAAATTTTGCGTCAAAATATTTAAGGGCGAACAGCATTACTCTCTTT
AAAGTACGTGAAGAAACTATCAAAGTTCTTGGAAAAGCTGACATGTGGTTTTTCAGTCCC
GAGCATCCTCCTTTGACTGAAGATGCACAAAAGGCTCTTGATTGGGCACTTGATGAAAAA
CTCAAATCCAGTGACAATGGGGAGATTACAACTACTCATTTGCTCCTTGGGGTGTGGTCA
CAAGTAGGATCACCAGGTTATAAGATATTGTCTGCTTTGGGCTTTAATGATGAAAAAGCT
CAAGAGTTGAAGAATGTAATTTCAGAACCTGGTTTTGTGGATGATTAA
>NtFTSH1-5a (2124 bp)
ATGGCCAATTCTCTCCTCTCTTCCAACTTCATGGGTTCTCAAATCTTTGTCTCTCCTCCC
ACCCCTAAAACAACAAAGTATTTCCATTTTCACTCCAAAAGAAAGTCTTTAATCCCTCAA
TCAATTCTCAACAAAAAACCCAATTCAGATAATTCAAAGAATATTCCTTCAAAAGCTGCT
TTAGCTGCTTTACTCTTTTCTTCAATCACTCCACATGCCTATGCTCTTGATAATACTACC
CCTACAGTACCAACCCCTCAAGTGATTCAAGCTGAAGCAGCCAATCCCACCACTTCAAAT
CCATTCTCTCAAAATATAATCTTGAATGCTCCAAAGCCTCAAGCACAGACCAATCCTGAA
CTTCCAGAAGTTTCTCAATGGAGATACAGTGAGTTCTTGAATGCTGTAAAGAAGGGTAAA
GTTGAAAGAGTCAGATTCAGTAAAGACGGAACTACCCTTCAGCTTAATGCTGTTGATGGC
CGTAGAGCTAGTGTAATTGTGCCTAATGACCCGGATTTAATTGACATTTTGGCTATGAAT
GGTGTTGATATATCAGTTTCTGAAGGTGATTCTGGTGGTAATGGGTTGTTTAATTTAATT
GGAAGTTTATTCCCTTTTATTGCTTTTGCTGGATTGTTCTATCTTTTCCAGAGATCTCAA
GGTGGGCCTGGTGGGCCTGGTGGGCTTGGTGGGCCCATGGATTTTGGTAGATCAAAGTCC
AAGTTTCAAGAAGTTCCTGAAACTGGAGTGTCTTTTGCTGATGTTGCTGGTGCTGATCAA
GCTAAATTGGAGTTACAAGAAGTAGTTGATTTTTTAAAGAATCCTGATAAGTATACAGCT
TTAGGTGCTAAAATACCAAAAGGGTGTCTTTTGGTGGGACCACCTGGTACAGGAAAGACA
CTTTTGGCTAGAGCAGTTGCTGGTGAAGCTGGTGTACCATTTTTCTCATGTGCACCATCA
GAGTTTGTTGAGTTGTTTGTTGGTGTTGGAGCTTCTAGAGTGAGGGATTTGTTCGAGAAG
GCGAAGTCGAAAGCGCCTTGCATTGTGTTTATTGATGAGATTGATGCTGTGGGAAGGCAG
AGAGGTGCAGGAATGGGAGGTGGGAATGATGAGAGAGAGCAGACTATTAATCAACTCTTG
ACTGAAATGGATGGTTTTTCTGGAAATAGTGGAGTAATTGTTTTGGCTGCAACCAATAGG
CCTGATGTTCTTGATTCTGCATTGTTGAGACCTGGGAGGTTCGATCGACAAGTGACTGTC
GACAGGCCTGATGTTGCTGGTAGAATCAAGATTCTTCAGGTGCATTCTAGAGGAAAGGCC
CTTGCAAAGGATGTGGACTTTGAGAAGATTGCCAGGAGAACACCGGGTTTCACTGGTGCA
GATTTGCAAAACTTGATGAATGAAGCAGCGATCCTTGCAGCTAGGCGTGAACTAAAGGAA
ATAAGTAAAGATGAGATATCTGATGCTTTGGAGAGGATAATTGCTGGACCGGAGAAGAAA
AATGCTGTTGTCTCAGAGGAGAAGAAGAAGCTGGTAGCTTATCATGAGGCCGGCCATGCC
TTGGTTGGTGCACTTATGCCCGAGTATGATCCTGTTGCCAAGATATCTATTATTCCTCGG
GGCCAAGCTGGTGGTCTTACCTTCTTTGCCCCTAGCGAAGAAAGACTTGAGTCGGGCTTG
TACAGCAGGAGCTACCTAGAGAATCAAATGGCAGTTGCACTTGGTGGAAGGGTTGCTGAG
GAGGTTATTTTTGGACAAGACAACGTAACAACTGGGGCATCTAACGATTTCATGCAAGTT
TCACGAGTGGCAAGGCAGATGGTTGAGAGATTAGGGTTCAGCAAAAAGATCGGACAGGTT
GCCATTGGAGGAGGTGGAGGAAATCCTTTCCTAGGTCAACAGATGTCAACCCAGAAAGAC

TACTCCATGGCAACAGCCGATGTGGTTGATGCTGAAGTAAGGGAATTGGTTGAAAGAGCA
TATGAAAGGGCAACACAGATTATCACAACACACATTGACATCCTACACAAGCTTGCTCAG
CTGTTGATAGAGAAAGAAACTGTTGATGGTGAAGAGTTCATGAGCCTTTTCATCGATGGC
AAGGCCGAGCTATACATTTCATGA
>NtFTSH1-5b (2124 bp)
ATGGCCAATTCTCTGCTCTCTTCCAACTTCTTTGGTTCTCAAATCTTTGTCTCTCCTCCC
ACACCTAAAACCACAAAATATTTCCATTTTCACTCCAAAAGAAAGTCTTTAATCCCTCAA
TCAATTCTCAACAAAAAACCCAATTCAGATAATTTAAAGAATATTCCTTCAAAAGCTGCT
TTAGCTGCTTTACTATTTTCTTCAATCACTCCACATGCCTTTGCTCTTGACAATACTACC
CCTACAGTACCAACTCCTCAAGTGATTCAAGCTGAAGCAGCCAATCCCAGCACTTCAAAT
CCATTCTCTCAAAATATAATCTTGAATGCTCCAAAGCCTCAAGCACAGACCAATCCTGAA
CTTCCAGAAGTTTCTCAATGGAGATACAGTGAATTCTTGAATGCTGTGAAAAAGGGTAAA
GTTGAAAGGGTCCGATTCAGTAAAGACGGATCTGCCCTCCAGCTTACTGCTGTTGATGGC
CGTAGAGCTACTGTAACTGTGCCTAATGACCCGGATTTAATTGACATTTTGGCTATGAAT
GGTGTTGATATATCAGTTTCTGAAGGTGATTCTGCTGGTAATGGGTTGTTTAATTTAATT
GGAAATTTATTCCCTTTTATTGCTTTTGCTGGATTGTTCTATCTTTTCCAGAGATCTCAA
GGTGGGCCTGGTGGGCCAGGTGGGCTTGGTGGCCCCATGGATTTTGGTAGGTCAAAGTCA
AAGTTTCAAGAAGTTCCTGAAACTGGAGTGACTTTTGCTGATGTTGCTGGTGCTGATCAA
GCTAAATTGGAGTTACAAGAAGTGGTTGATTTTTTAAAGAATCCTGATAAGTATACAGCT
TTAGGTGCTAAAATACCAAAAGGGTGTCTTTTGGTGGGACCACCTGGTACAGGAAAGACA
CTTTTGGCTAGAGCAGTTGCTGGTGAAGCTGGTGTACCATTTTTCTCATGTGCAGCATCA
GAGTTTGTTGAGTTGTTTGTTGGTGTTGGAGCTTCTAGAGTGAGGGATTTGTTCGAGAAG
GCGAAGTCGAAAGCGCCTTGCATTGTGTTTATTGATGAGATTGATGCTGTGGGGAGGCAG
AGAGGTGCAGGAATGGGAGGTGGGAATGATGAGAGAGAGCAGACTATTAATCAACTCTTG
ACTGAAATGGATGGGTTTTCTGGAAATAGTGGAGTCATTGTTTTGGCTGCAACTAATAGG
CCTGATGTTCTTGATTCTGCATTGTTGAGACCTGGGAGGTTCGATCGACAAGTGACTGTC
GATAGGCCTGATGTTGCTGGTAGAATCAAGATTCTTCAGGTGCATTCTAGAGGAAAGGCC
CTTGCAAAGGATGTGGACTTTGAGAAGATTGCCAGGAGAACACCGGGTTTCACTGGTGCA
GATTTGCAAAACTTGATGAACGAAGCAGCAATCCTTGCAGCTAGGCGTGAACTAAAGGAA
ATAAGTAAAGATGAGATATCTGATGCTCTGGAGAGGATAATTGCCGGCCCAGAGAAGAAA
AATGCTGTTGTCTCAGATGAGAAGAAGAAGCTGGTAGCTTATCATGAGGCCGGCCATGCC
TTGGTTGGTGCACTTATGCCCGAGTATGATCCTGTTGCCAAGATATCTATAATTCCTCGA
GGCCAAGCTGGTGGTCTTACCTTCTTTGCCCCCAGCGAAGAAAGACTTGAGTCGGGCTTG
TACAGCAGGAGCTACCTAGAGAATCAAATGGCAGTTGCACTTGGTGGAAGGGTTGCTGAG
GAGGTTATTTTTGGACAAGATAACGTAACAACTGGGGCATCTAACGATTTCATGCAAGTT
TCACGAGTGGCAAGGCAGATGGTTGAGAGATTAGGGTTCAGCAAAAAGATTGGACAAGTT
GCCATTGGAGGAGGTGGAGGAAATCCTTTCCTAGGTCAACAGATGTCAACCCAGAAAGAC
TACTCCATGGCTACAGCCGATGTGGTTGATGCTGAAGTAAGGGAATTGGTTGAAAGAGCA
TATGAAAGGGCAACAGAGATTATCACAACACACATTGACATCCTACACAAGCTTGCTCAG
CTGTTGATAGAGAAAGAAACTGTTGATGGTGAAGAGTTCATGAGCCTTTTCATCGATGGC
AAGGCCGAGCTATACATTTCATGA
>NtFTSH2-8a (2082 bp)
ATGTCTACTTCATCAGTATGCATAGCAGGAAATAGTTTGTCCACTCATAGAAAGCAGAAA
GTTTTCAGGAAGGAGATTTATGGCAGGAAAATTTTATTCTCCCCAAATCTTCCATCCTCT
AGTAAAACATCGAGAATAGCTGTAAAATCATCTCTTCAGCAAAGGCCAGATGAAGGAAGA
AGAGGCTTTCTCAAATTATTGCTTGGAAATGTTGGGCTTGGAGTACCTGCTTTGTTAGGT
GATGGAAAAGCCTACGCTGATGAGCAAGGTGTTTCTAACTCAAGGATGTCTTATTCTAGA
TTTTTGGAGTATTTGGACAAGGATAGGGTGCAAAAAGTAGATTTGTTCGAAAACGGGACC
ATAGCTATTGTTGAGGCTGTATCTCCAGAATTAGGAAACCGGGTTCAGAGGGTTCGGGTA
CAACTACCTGGGCTCAGCCAGGAACTCCTTCAGAAGTTGCGGGAAAAGAACATCGACTTT
GCTGCTCACAATGCCCAAGAGGACTCGGGTTCTTTCCTATTCAACTTGATTGGGAATCTG
GCATTCCCGCTTATTTTGATTGGTGGTCTTTTCCTGCTATCAAGGCGGTCTCCCGGAGGA
ATGGGAGGTCCTGGTGGGCCTGGTAACCCATTAGCGTTTGGTCAATCAAAGGCTAAGTTC
CAAATGGAGCCAAACACTGGTGTAACGTTTGATGATGTTGCTGGTGTAGATGAGGCAAAA
CAAGATTTTATGGAGGTAGTAGAATTTTTGAAGAAGCCCGAGAGGTTTACCGCAGTGGGG
GCTCGTATTCCAAAAGGTGTTCTTCTTGTTGGTCCTCCTGGTACTGGGAAAACCCTGCTA
GCAAAGGCAATTGCTGGTGAAGCGGGTGTTCCATTTTTCTCAATTTCAGGTTCAGAATTT
GTTGAGATGTTTGTTGGTGTAGGAGCCTCTCGAGTCCGCGATCTTTTCAAGAAGGCCAAG
GAAAATGCTCCCTGCATTGTATTTGTTGATGAAATTGATGCTGTTGGGCGGCAAAGAGGG
ACTGGAATTGGAGGAGGGAATGATGAAAGGGAACAGACCCTGAACCAACTATTGACGGAA
ATGGATGGTTTCGAAGGAAATACTGGTATAATAGTTGTTGCGGCAACCAATCGTGCAGAT
ATTCTTGACTCTGCTTTGCTGAGGCCAGGGCGATTTGATAGACAAGTAAGTGTGGATGTT

CCAGATATCAAGGGAAGAACAGAGATCTTAAACGTTCACGCCGGCAACAAGAAGTTCGAT
TCCGATGTTTCTCTTGAAGTTATAGCCATGAGGACTCCCGGTTTCAGTGGTGCAGACCTT
GCTAACCTCTTAAATGAAGCAGCCATTCTTGCCGGTCGACGTGGTAAGACGGCAATCGCG
TCCAAAGAGATTGATGATTCAATTGATAGGATAGTGGCTGGAATGGAAGGAACAGTCATG
ACTGATGGCAAGAGCAAGAGTCTGGTGGCATACCACGAAGTTGGACATGCCATCTGTGGA
ACTCTTACTCCAGGGCATGATGCTGTTCAAAAGGTCACACTAATCCCACGTGGTCAGGCA
AAAGGTTTGACCTGGTTCATTCCTGCAGATGATCCAACCTTAATATCCAAGCAGCAACTC
TTTGCTAGAATTGTCGGAGGACTTGGGGGAAGAGCTGCAGAGGAAGTTATCTTTGGTGAA
CCTGAGGTGACCACTGGTGCTGCAGGCGATTTGCAGCAGATCACCGGTTTGGCAAAACAG
ATGGTTGTCACTTTTGGGATGTCTGAACTTGGCCCGTGGTCACTCATGGATTCTTCAGCC
CAAAGTGGTGATGTAATCATGAGAATGATGGCCAGGAATTCTATGTCAGAAAAGCTAGCT
GAAGACATCGATGGTGCTGTGAAGAGGCTTTCAGACAGCGCATATGAGATTGCATTGACC
CACATCCGCAACAACCGTGAAGCAATTGATAAGATTGTGGAAGTCCTCCTTGAAAAGGAG
ACGATGACTGGAGATGAATTCCGCGCTATTCTCTCAGAATTTGTTGAAATTCCTGCTGAA
AACCGAGTTGCTCCTGTTGTACCTACCCCAGCAACTGTATAA
>NtFTSH2-8b (2082 bp)
ATGGCTACTTCATCAGTATGCATAGCAGGAAATAGTTTGTCTACTCATAGAAGGCAGAAA
GTTTTCAGGAAGGAGATTTATGGCAGGAAAATTTTATTTTCCTCAAATCTTCCATCGTCT
AGTAAAACATCGAGAATAGCTGTAAAAGCATCCCTTCAGCAAAGGCCAGATGAAGGAAGA
AGAGGTTTTCTCAAATTATTGCTTGGAAATGTTGGGCTTGGAGTACCGGCTTTGTTAGGT
GATGGAAAAGCCTACGCTGATGAGCAAGGTGTGTCTAACTCAAGGATGTCGTATTCTAGA
TTTTTGGAGTATTTGGATAAGGATAGGGTGCAAAAAGTAGATTTGTTTGAAAACGGAACC
ATAGCTATTGTTGAGGCTATATCTCCAGAATTAGGAAACCGGGTTCAGAGGGTTCGGGTA
CAACTACCTGGGCTCAGCCAGGAACTCCTTCAGAAGTTGCGAGAAAAGAACATTGACTTT
GCTGCTCACAATGCCCAAGAGGACTCGGGTTCTTTCCTATTCAACTTGATTGGGAATCTG
GCATTCCCGCTTATTTTGATTGGTGGTCTTTTCCTGCTATCAAGGCGGTCTCCCGGAGGA
ATGGGAGGTCCTGGTGGGCCTGGTAACCCATTAGCATTTGGTCAATCAAAGGCTAAGTTC
CAAATGGAGCCAAACACTGGTGTAACATTTGATGATGTTGCTGGTGTAGATGAAGCAAAA
CAAGATTTTATGGAGGTCGTAGAATTTTTGAAGAAGCCCGAGAGGTTTACCGCAGTGGGG
GCTCGTATTCCAAAAGGTGTTCTTCTTGTTGGTCCTCCTGGTACTGGGAAGACCCTGCTA
GCAAAGGCAATTGCTGGTGAAGCGGGTGTTCCATTTTTCTCAATTTCAGGTTCAGAATTT
GTTGAGATGTTTGTTGGTGTAGGAGCCTCTCGAGTCCGTGATCTTTTCAAGAAGGCCAAG
GAAAATGCTCCCTGCATTGTATTTGTTGATGAAATTGATGCTGTTGGGCGGCAAAGAGGG
ACTGGAATTGGAGGAGGGAATGATGAAAGGGAACAGACCCTGAACCAACTATTGACAGAA
ATGGACGGTTTCGAAGGAAATACTGGTATAATAGTTGTTGCGGCAACCAATCGTGCAGAT
ATTCTTGACTCTGCTTTGCTGAGACCAGGGCGATTTGATAGACAAGTAAGTGTGGATGTT
CCAGATATCAAGGGAAGAACAGAGATCTTAAAGGTTCACGCGGGCAACAAGAAGTTCGAT
TCTGATGTTTCTCTTGAAGTTATAGCCATGAGGACACCCGGTTTTAGTGGTGCAGATCTT
GCTAACCTCTTAAATGAAGCAGCCATTCTTGCTGGTCGGCGTGGTAAGACAGCAATCGCA
TCCAAAGAGATTGATGATTCAATTGATAGGATAGTGGCTGGAATGGAAGGAACAGTCATG
ACTGATGGCAAGAGCAAGAGTCTGGTGGCATACCACGAAGTTGGACATGCCATCTGTGGA
ACTCTCACTCCAGGGCATGATGCTGTTCAAAAGGTCACATTAATCCCACGTGGTCAGGCA
AAAGGTTTGACCTGGTTCATTCCTGCAGATGATCCAACCTTAATATCCAAGCAGCAACTC
TTTGCTAGAATTGTCGGAGGACTTGGGGGAAGAGCTGCAGAGGAAGTTATCTTTGGTGAA
CCTGAGGTGACCACTGGTGCTGCAGGCGATTTGCAGCAGATCACCGGTTTGGCAAAACAG
ATGGTTGTCACTTTTGGGATGTCTGAACTTGGCCCATGGTCACTCATGGATTCTTCTGCC
CAAAGTGGTGATGTAATCATGAGAATGATGGCTAGGAATTCTATGTCAGAAAAGCTAGCT
GAAGACATTGATGGTGCTGTGAAGAGGCTTTCAGACAGCGCATATGAGATTGCATTGACC
CATATCCGCAACAACCGTGAAGCAATTGATAAGATTGTGGAAGTCCTCCTTGAAAAGGAG
ACGATGACCGGAGATGAATTCCGCGCTATTCTCTCAGAATTTGTTGAAATTCCTGCTGAA
AACCGAGTTGCTCCTGTTGTACCTACCCCAGCAACTGTATAA

Supplementary Table S3. Nucleotide sequences of the regions targeted by RNAi to trigger gene silencing for Clp and FtsH subunits.

Protease subunit	Sequence of the gene region targeted by RNAi
NtCLPP6	GGATAAAGCCTAAGGTTGGTACAGTATGTTTCGGAGTAGCTGCAAGCCAAGGAGC ACTTCTTCTTGCCGGTGGAGAAAAGGGCATGAGGTATGCAATGCCAAATGCACGC ATAATGATTCATCAACCTCAAAGTGGTTGTGGAGGTCATGTGGAAGATGTGCGGC GCCAAGTGAACGAAGCGGTTCAATCTCGTCATAAAATCGACAAGATGTATGTCGC CTTTACTGGCCAACCAATTGAGAAGGTGCAACAGTACACTGAAAGGGATCGTTTTT TGTCTGTCTCAGAGGCCATGGAGT
NtCLPR2	ATTTTCATCGGACAACACATAGATGAAGAATTTAGCAACCAGATATTGGCAACAA TGCTGTATCTTGACAGTATTGATGATTCCAAGAAGCTCTACCTGTATATCAATGGC CCTGGTGGTGATCTAACTCCAAGCATGGCCATCTACGACACAATGCAAAGTCTGA AAAGTGCTGTTGGCACCCATTGTGTGGGCTATGCCTACAATCTTGCCGGTTTTCTTC TTGCTGCTGGAGAAAAGGGCAATCGATTTGCAATGCCTCTTTCAAGGATTGCACTA CAATCTC
NtCLPS	CCCCGCCCTATCGTGTGATGCTGCACAATGACAACTACAACAAGAGGGAGTATGT AGTTCAAGTACTCATGAAGGTTATACCGGGGATGACAGTCGACAATGCTGTTAAT ATCATGCAAGAGGCGCATTACAATGGTCTGGCGGTGGTGATAATCTGTGCTCAAG CTGATGCAGAAGAGCATTGCACGCAGCTGAGAGGCAATGGTCTGCTAAGCTCCAT TGAGCCTGCCAGTGGAGGTTGT
NtCLPC	AGTGTGATAGAGAAAGGAGGCCGTCGTATAGGTTTTGATCTAGATTATGATGAGA AGGATAGCAGTTACAACCGTATCAAGAGCTTGGTGACTGAGGAGTTGAAACAGTA CTTCAGGCCAGAGTTCTTGAACAGATTGGATGAGATGATTGTATTCCGTCAGCTCA CTAAGTTAGAGGTGAAGGAGATAGCTGATATCATGCTTAAGGAGGTCTTTGAGAG GTTGAAAAATAAGGAGATAGAACTTCAAGTGAC
NtCLPD	CCCTGATAGTGATGACTTCTAATGTGGGTTCTACAGCCATAGTAAAGGGTAGACA GAATACTATTGGCTTCTTGCTTGCTGATGATGAATCAGCAGCCTCCTATGCTGGTA TGAAAGCAATAGTGATGGAAGAGCTAAAGACATATTTCCGCCCAGAGTTGATGAA TAGGCTAGACGAAGTAGTAGTATTCCGTCCTCTAGAGAAGCCCCAGATGCTCCAG ATACTAGACCTGATGCTGCA
NtCLPT1-T2	AAGCTCTTTTAATGGGAATCTTGGT**C**GAAGGAACCAGTTTGGCTGCCAAGTTT**T**TG AGAGCAAACGGTGTAACCCTTCTCAAGGCGAGGGATGAAACTGTAAAGTTGCTTG GAAAATCCGATATGTATTTTTTCAGTCCAGAGCATCCTCCCCTTACTGAACCAGCT CAAAGGGCTCTTGACTGGGCAGTCGATGAGAAATACTTGGAAAAGCTGACATGTG GTTTTTTAGTCCTGAGCATCCTCCTTTGACTGAAGATGCACAAAAGGCTCTTGATT GGGCGGTCGATGAAAAACTCAAATCCAGTGACAATGGGGAGATAACAACTACTCA TTTGCTCCTTGGGGTGTGGTCACAAGTAGGATCACCAGGTTATAAGATATTGTCTG CTTTGGGCTT
NtFTSH1-5	TATATGATTGCCTTTTACCCCACTGTTTTAGAAAGAAAAAAATCTCCATATTCCCC AAAATTTAAGAACAAGAATTGATCAGTAGTACAACTAAGACCAAATCAAACAAAC ACCTTCCAAATTTAGTTATGGCCAATTCTCTGCTCTCTTCCAACTTCTTTGGTTCTC AAATCTTTGTCTCTCCTCCCACCCCTAAAACCACAAAGTATTTCCATTTTCACTCCA AAAGAAAGTCTTTAATCCCTCAATCAATTCTCAACAAAAAACCCAATTCAGATAA

Supplementary Table S4. List of oligonucleotides used for construction of RNAi vectors and RT-PCR experiments.

**Name**	**Sequence 5'-3'**
ClpP6gateF'	CACCGGATAAAGCCTAAGGTTGGTA
ClpP6gateR'	ACTCCATGGCCTCTGAGACAG
ClpR2gateF'	CACCATTTTCATCGGACAACACAT
ClpR2gateR'	GAGATTGTAGTGCAATCCTTG
ClpSgateF'	CACCCCCCGCCCTATCGTGTGATG
ClpSgateR'	ACAACCTCCACTGGCAGGCTC
ClpCgateF'	CACCAGTGTGATAGAGAAAGGAGG
ClpCgateR'	GTCACTTGAAGTTCTATCTCC
ClpT1gateF'	CACCAAGCTCTTTTAATGGGAATC
ClpT2gateR'	AAGCCCAAAGCAGACAATATC
ClpDgateF'	CACCCCCTGATAGTGATGACTTCT
ClpDgateR'	TGCAGCATCAGGTCTAGTATCTGG
FtsH15gateF'	CACCTATATGATTGCCTTTTACCCC
FtsH15gateR'	TTATCTGAATTGGGTTTTTTGTTGAG
ClpP3_1 for	TCTCGGAACTGGGATGTTTC
ClpP3_2 rev	GATTCCCATCCCAGCAGTTA
ClpP4_1 for	CCTCACTGTTCCTCCCTCAA
ClpP4_1 rev	GAGAAGGGGGTTTTTGAAGC
ClpP5_2 for	TGTTGATCCCACAAAGGACA
ClpP5_2 rev	CCAAGAGGCTGGTGAATCAT
ClpP6_1 for	GAGAACCCCGTTAAAGCTCA
ClpP6_1 rev	AGTTGCGAGGGTCACAAGTT

ClpR1_1 for	ATAACCCAGTACGGCGACAG
ClpR1_1 rev	GGCATGCCCAGATAGACAAT
ClpR2_1 for	GGGATTGCAGCGTCTAATGT
ClpR2_1 rev	CACGTTCACGGTAAAGAGCA
ClpR3_2 for	GTGCCAGCAGTCACAGAGTT
ClpR3_2 rev	CAGCTGCAAGCAAGAGACAC
ClpR4_1 for	CTCTTCGCCCTTCTTCCTCT
ClpR4_1 rev	TGGCTTTTCCTCATCCTCAT
ClpS_2 for	TCCCCATCAAATCTTCCAAC
ClpS_rev	GAATTCAGATTCACGCCCAG
ClpT1_for	TCAACTTCGACTTCTTACAA
ClpT1_rev	GAATTCAGATTCACGCCCAG
ClpT2_1_2 for	TAGGACCCAATTCGTCGTTC
ClpT2_1 rev	ATTCACCCATGGCAAATGAT
ClpC_3 for	GGCGTCGACCTTACACTGTT
ClpC_3 rev	TCACACTGCTTCCGACATTC
ClpD_2 for	AATGCTGCTGTGCAACTGTC
ClpD_2 rev	TGCTTGCCAAGATCACTTCA
clpP_GTG_for	GGAACACCAATAGGCACTAAATGAAAGAAAG
clpP_GTG_rev	CTTTCTTTCATTTAGTGCCTATTGGTGTTCC
clpP_TTG_for	GGAACACCAATAGGCAATAAATGAAAGAAAG
clpP_TTG_rev	CTTTCTTTCATTTATTGCCTATTGGTGTTCC
Pseq_clpP_start	GAAACGGGGGATATCTCGATCGGG
PaadA136	TCGATGACGCCAACTACC
PaadA25a	AGATCACCAAGGTAGTCGGCAA

PpsbB_for	ACTCGTTTAGGAATAACCAATT
PpsbB_rev	GCAATCCTTTGTATAGACGT
PpsaB_for	CCCAGAAAGAGGCTGGCCC
PpsaB_rev	CCCAAGGGGCGGGAACTGC

Supplementary Table S5. Targeting prediction for tobacco Clp and FtsH subunits. The known location of the homologous proteins from *Arabidopsis* is also indicated.

**Gene**	***Arabidopsis* homologs**	**Location in *Arabidopsis***	**Tobacco homolog**	**Targeting prediction**
CLPP6	*AtCLPP6*	Stroma	Nt*CLPP6*	Chloroplast
CLPR2	*AtCLPR2*	Stroma	Nt*CLPR2*	Chloroplast
CLPC	*AtCLPC1*	Stroma	Nt*CLPC*	Chloroplast
CLPS	*AtCLPS*	Stroma	Nt*CLPS*	Chloroplast
CLPD	*AtCLPD*	Stroma	Nt*CLPD*	Chloroplast
CLPT1	*AtCLPT1*	Stroma	Nt*CLPT1*	Chloroplast
CLPT2	*AtCLPT2*	Stroma	Nt*CLPT2*	Chloroplast
FTSH1-5	*AtFTSH1*<br>*AtFTSH5*	Thylakoid membrane	Nt*FTSH1-5*	Chloroplast
