# Supplementary material for: Generation and characterization of a collection of knock-down lines for the chloroplast Clp protease complex in tobacco
Source: J Exp Bot. 2017 Mar 28;68(9):2199–218. doi: 10.1093/jxb/erx066 (PMC5447895; doi:10.1093/jxb/erx066)
Supplement: Supplementary_Protocols [file erx066_suppl_Supplementary_Protocols.pdf]

Supplementary Protocols

Assembly of tobacco sequences for chloroplast protease subunits

High-scoring sequences from the EST and GSS databases were collected and used as queries in subsequent BLASTx searches against TAIR10 and ITAG2.3 proteins and only sequences best-matching to the candidates were kept. These sequences were assembled into initial contigs with DNASTAR SeqMan Pro using default parameters. Subsequently, the initial contigs were supplemented with the sequence hits from the SRR database and the resulting final contigs were manually curated. For most candidates, this yielded contigs covering the expected full-length coding sequence and containing both parental gene versions in the allotetraploid tobacco genome. The latter is reflected by the presence of single nucleotide polymorphisms (SNPs) between the parental alleles.
